# GMM-Demux: sample demultiplexing, multiplet detection, experiment planning, and novel cell-type verification in single cell sequencing

**DOI:** 10.1186/s13059-020-02084-2

**Published:** 2020-07-30

**Authors:** Hongyi Xin, Qiuyu Lian, Yale Jiang, Jiadi Luo, Xinjun Wang, Carla Erb, Zhongli Xu, Xiaoyi Zhang, Elisa Heidrich-O’Hare, Qi Yan, Richard H. Duerr, Kong Chen, Wei Chen

**Affiliations:** 1 University of Michigan-Shanghai Jiao Tong University Joint Institute, Shanghai Jiao Tong University, Shanghai, 200240 China; 2 Department of Pediatrics, School of Medicine, University of Pittsburgh, Pittsburgh, 15260 USA; 3 Department of Automation, Tsinghua University, Beijing, 100086 China; 4 School of Medicine, Tsinghua University, Beijing, 100086 China; 5 Department of Medicine, School of Medicine, University of Pittsburgh, Pittsburgh, 15260 USA; 6 Department of Biostatistics, School of Public Health, University of Pittsburgh, Pittsburgh, 15260 USA

**Keywords:** Single cell RNA, Multiplets, Rare cell type, Phony cell type, Demultiplex, Sample barcoding

## Abstract

Identifying and removing multiplets are essential to improving the scalability and the reliability of single cell RNA sequencing (scRNA-seq). Multiplets create artificial cell types in the dataset. We propose a Gaussian mixture model-based multiplet identification method, GMM-Demux. GMM-Demux accurately identifies and removes multiplets through sample barcoding, including cell hashing and MULTI-seq. GMM-Demux uses a droplet formation model to authenticate putative cell types discovered from a scRNA-seq dataset. We generate two in-house cell-hashing datasets and compared GMM-Demux against three state-of-the-art sample barcoding classifiers. We show that GMM-Demux is stable and highly accurate and recognizes 9 multiplet-induced fake cell types in a PBMC dataset.

## Background

Droplet-based single cell RNA sequencing (scRNA-seq) [13, 18, 48] has provided many valuable insights into complex biological systems, such as rare cell-type identification [26, 32, 39, 41], differential expression analysis at the single cell level [2, 5, 9], and cell lineage studies [9, 15, 24, 30]. While the per-cell cost of library preparation has decreased over the years, the scalability of droplet-based scRNA-seq remains limited, mostly due to rapidly increasing, yet hard to anticipate, multiplet rates as more cells are loaded during single sequencing cell library preparation [17]. Multiplets significantly confound the analysis of single cell experiments and can lead to false discoveries [10, 17], such as false lineages in cell lineage tracing [14, 20, 29], incorrect categorizations in cell-type classification [27, 43, 49], or false findings in rare cell-type discovery [22, 44]. Large cell populations are especially required for rare cell-type discovery, but loading large cell populations during scRNA-seq library preparation leads to high multiplet rates. As a result, researchers are challenged with identifying real rare-type cells in a multiplet-filled scRNA-seq dataset. Overall, the scalability of scRNA-seq can be significantly improved, greatly reducing the per-cell library preparation cost, if multiplets can be identified and removed from downstream analysis. To achieve greater adoption of single cell sequencing technology, it is crucial to (1) identify and remove multiplets from downstream analysis, (2) anticipate the multiplet rate prior to conducting an experiment, and (3) verify whether rare cell types identified from a single cell dataset are authentic and are not multiplets.

Recently, emerging sample barcoding technologies, such as cell hashing [36] or MULTI-seq [21], enable identification of multiplets arising from more than one uniquely labeled sample and facilitate their subsequent removal from downstream analysis. Both methods use oligonucleotide-labeled reagents that conjugate on the cell surface to produce sample-specific markings on cells: cell hashing, an extension of the cellular indexing of transcriptomes and epitopes by sequencing (CITE-seq) technology [35], uses barcoded oligo-conjugated antibodies that target ubiquitously expressed surface markers, such as CD298 and beta2-microglobulin, while MULTI-seq uses lipid- and cholesterol-modified oligonucleotides that attach to the cell surface membrane and the cell nuclei membrane. For simplicity, we refer to the oligonucleotide-labeled reagents used in both methods as sample-hashtag oligonucleotides (HTOs). Sample barcoding involves labeling cells from each sample with sample-specific HTO conjugates and then pooling the HTO-labeled cells from different samples for droplet-based scRNA-seq sequencing library preparation. During library preparation, the pooled cell assay is driven through a microfluidic chip to form cell-assay droplets. A fraction of cell-assay droplets are combined with barcode-enclosing gel beads and form Gel Beads in Emulsion, or GEMs. Inside each GEM, HTO barcodes are combined with GEM barcodes. Subsequent sequencing simultaneously recovers the HTO barcode(s) and the GEM barcode for each GEM. An abstract workflow of a 3-sample sample barcoding experiment is provided in Additional file 1: Fig. S2. Finally, the count of the HTO unique molecular identifiers (UMIs) for each sample, which translates to the number of cell-attached, sample-specific HTO antibodies of each GEM, is summarized in a matrix, called the HTO matrix. Table 1 depicts an example 3-sample HTO matrix.

**Table 1:** An example HTO matrix. Each row is a GEM with its unique GEM barcode as index. Each column is a HTO sample ID. The *i*th row and *j*th column of the matrix store the number of HTO antibodies (in the form of UMI counts) of the *j*th HTO sample (HTO-*j*) attached to cells in the *i*th GEM

GEM barcode	HTO 1	HTO 2	HTO 3
ACTAGGACCA	20	45	723
TCGGACTCGG	561	23	15
GCAGTAGGCA	742	593	14
CCAGACATGA	31	747	39
CCTAGACTTA	21	15	33

**Fig. 1:**
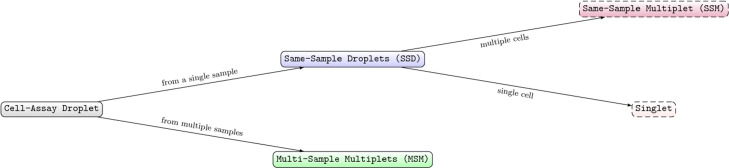
Relationship between MSM, SSM, SSD, and singlet. SSD and MSM are differentiated based on whether the droplet contains cells from multiple HTO samples. SSM and singlet are further differentiated by the number of cells in the droplet

There are three types of droplets in a sample barcoding scRNA-seq dataset: (1) *multi-sample multiplets* (*MSMs*), droplets that contain more than one cell from more than one HTO sample; (2) *single-sample multiplets* (*SSMs*), droplets that contain more than one cell from a single HTO sample; and (3) *singlets*, droplets that contain a single cell. We combine singlets and SSMs into a single category called *single-sample droplets* (*SSDs*) to differentiate them from MSMs. The relationship between MSM, SSM, singlet, and SSD is summarized in Fig. 1. MSMs can be distinguished from SSDs in the HTO matrix: MSMs typically have high HTO UMI counts from more than one HTO barcode, while SSDs typically have high UMI counts from a single HTO barcode and low HTO UMI counts from all other HTO barcodes. However, sample barcoding cannot separate singlets from SSMs, as these two droplet types are indistinguishable in the HTO matrix. As a result, SSMs cannot be removed by sample barcoding and will remain in the dataset as noise.

**Fig. 2:**
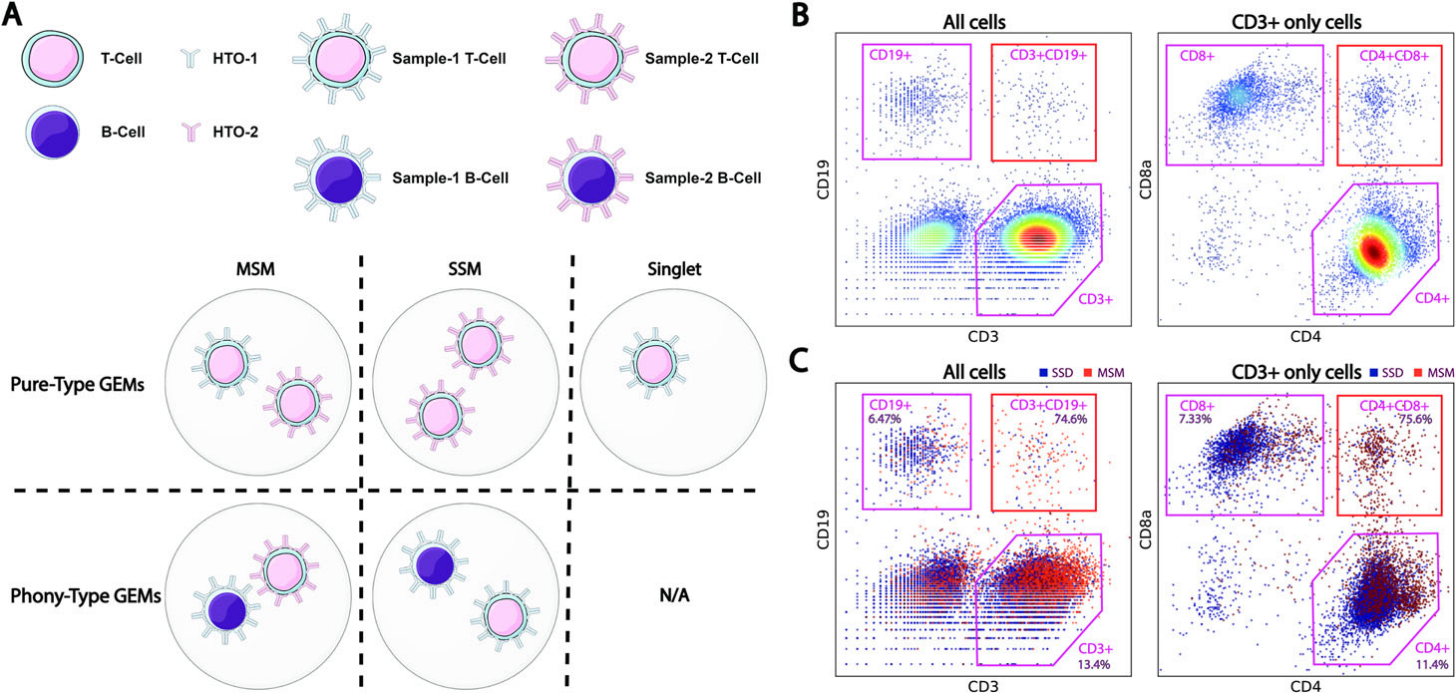
Examples of pure-type and phony-type GEMs in scRNA-seq data. **a** Example compositions of pure-type and phony-type GEMs in a cell-hashing dataset. Note that phony-type GEMs cannot be singlets. **b** The gating results of a 4-sample cell-hashing CITE-seq PBMC dataset. Notice the unconventional cell types in **b** (highlighted in red bounding boxes). **c** The MSM ratio of each cell type in **b**. The alleged novel cell types in B are all phony-type cells, highlighted by their high MSM ratios

GEMs can also be classified based on the number of cell types enclosed in them. GEMs that contain a single cell type are named *pure-type GEMs* whereas GEMs that contain multiple cell types are named *phony-type GEMs*. An illustration of phony-type GEMs and pure-type GEMs is provided in Fig. 2a. Pure-type GEMs are not necessarily singlets—a pure-type GEM can still be a multiplet, but contains cells of exactly the same cell type. Hence, a pure-type GEM could be a singlet, a MSM, or a SSM. Phony-type GEMs, on the other hand, are all multiplets. Hence, they must be either MSMs or SSMs.

Phony-type GEMs can be misclassified as novel rare cell types. Figure 2b depicts the gating results of a 16K-GEM PBMC cell-hashing and CITE-seq dataset. The CD3-CD19 scatter plot shows GEMs comprised of CD19^+^ B cells, CD3^+^ T cells, and also a CD3^+^CD19^+^ double-positive T-B cell GEM cluster. Similarly, in the CD4-CD8 scatter plot of all CD3^+^ T cells, besides the CD4^+^ helper T cells and CD8^+^ cytotoxic T cells, there also exists a CD4^+^CD8^+^ helper-cytotoxic T cell GEM cluster. Both clusters are highlighted in red circles. Existence of such PBMC types at the observed frequencies is unlikely, as CD3^+^CD19^+^ cells and CD4^+^CD8^+^ T cells are believed to be extremely rare [1, 33]. In fact, as revealed by cell hashing, both cell types, along with many other alleged novel rare cell types discovered in this dataset, are all phony cell types. Most, if not all, GEMs in these phony-cell-type clusters are phony GEMs: instead of containing real T-B cell(s), each CD3^+^CD19^+^ GEM is a multiplet that contains individual CD3^+^ T and CD19^+^ B cell(s). When compared against true cell types, such as CD19^+^ B cells or CD4^+^ helper T cells, phony-type GEMs are most likely to be MSMs. Figure 2c displays the MSM ratios of the CD19^+^ B cell, the CD4^+^ helper T cell, and the CD8^+^ cytotoxic T cell true-cell-type GEM clusters (also referred to as pure-type GEM clusters), as well as the MSM ratios of the CD3^+^CD19^+^ and the CD4^+^CD8^+^ phony-cell-type GEM clusters (or simply phony-type GEM clusters). From the figure, we observe that phony-cell-type GEM clusters have much higher MSM ratios than true-cell-type GEM clusters (∼75*%* vs. <14*%*).

Existing MSM classifiers, including the *heuristic classifier* from Seurat [4, 36], the *heuristic classifier* from MULTI-seq [23], and the *model-based classifier* demuxEM [8], suffer from one or multiple shortcomings, including low classification accuracy, non-deterministic output, unreliable heuristics, and inaccurate model assumptions. Additionally, existing classifiers do not model SSM. Therefore, they cannot estimate the percentage of singlets and SSMs in the dataset and they cannot predict the percentages of MSMs, singlets, and SSMs of the conceived output of a planned sample barcoding experiment. Most importantly, without a droplet formation model, they cannot determine whether an alleged novel cell type-defining GEM cluster consists of mainly pure-type GEMs. Hence, they are not able to (and are not designed to) use the sample barcoding information to authenticate the legitimacy of putative novel cell types in a scRNA-seq dataset.

In this work, we propose a model-based Bayesian framework, GMM-Demux, for sample barcoding data processing. GMM-Demux consistently and accurately separates MSMs from SSDs; estimates the percentage of SSMs and singlets among SSDs; anticipates the MSM, SSM, and singlet rates of planned future sample barcoding experiments; and verifies the legitimacy of putative novel cell types discovered in sample-barcoded scRNA-seq datasets. Specifically, GMM-Demux independently fits the HTO UMI counts of each sample into a Gaussian mixture model [34]. From each Gaussian mixture model, GMM-Demux computes the posterior probability of a GEM containing cells from the corresponding sample. From the posterior probabilities, GMM-Demux computes the probabilities of a GEM being a MSM or a SSD. Among SSDs, GMM-Demux estimates the proportion of SSMs and singlets in each sample using an augmented binomial probabilistic model. Using the probabilistic model, GMM-Demux checks if a proposed putative cell type-defining GEM cluster is a pure-type GEM cluster or a phony-type GEM cluster, and based on the classification of the GEM cluster, GMM-Demux proves or rejects the novel cell-type proposition.

To benchmark the performance of GMM-Demux, we conducted two in-house cell-hashing and CITE-seq experiments; collected a public cell-hashing dataset; and simulated 9 in silico cell-hashing datasets. We compare GMM-Demux against three existing, state-of-the-art MSM classifiers and show that GMM-Demux is highly accurate and has the most consistent performance among the batch. From the cell-hashing and CITE-seq PBMC dataset, we extracted 9 putative novel type GEM clusters through in silico gating, Further analysis by GMM-Demux shows that all 9 putative novel-type GEM clusters are phony-type GEM clusters and are removed from the dataset. Out of the 15.8K GEMs of the PBMC dataset, GMM-Demux identifies and removes 2.8K multiplets, reducing the multiplet rate from 23.9 to 6.45%. After removing all phony-type GEM clusters, GMM-Demux further reduces the multiplet rate to 3.29%.

## Results

### Datasets

#### Real datasets

We benchmark GMM-Demux on three separate HTO datasets from three independent sources. In addition to a public dataset from Stoeckius et al. [36] (PBMC-2), we conducted two additional in-house cell-hashing experiments independently in two separate labs (PBMC-1, Memory T). A summary of the three datasets is provided in Table 2.

**Table 2:** Summary of cell-hashing datasets

Name	Est. no. of cells	No. of GEMs	No. of samples	Tissue	Source
PBMC-1	35,685	15,841	4	PBMC	In-house
Memory T	25,000	9715	5	CD4^+^ Memory T cells	In-house
PBMC-2	28,000	15,455	8	PBMC	Stoeckius et al. [36]

Cells in the PBMC-1 dataset are drawn from a healthy donor following the same protocol described in a previous study [38]. These cells are divided into four samples. Each sample is subjected to the Totalseq-A and cell-hashing protocol [36], targeting a recovery of ∼ 5000 cells per sample. All HTO-tagged cells are pooled together and are prepared using the 10X Genomics platform with Gel Bead Kit V2. The prepared assay is subsequently sequenced on an Illumina Hiseq platform with a depth of 50K reads per cell. In addition to cell hashing, cells in this dataset are simultaneously measured for their surface marker abundance through CITE-seq [35]. Eight surface markers are measured for every cell: CD3, CD4, CD8, CD11, CD14, CD16, CD19, and CD56.

Cells in the CD4^+^ Memory T dataset were enriched from the peripheral blood of a healthy adult human volunteer using the MACSxpress® Whole Blood CD4 Memory T Cell Isolation Kit, human (Miltenyi Biotec). The cells were then incubated for 12 h at 37 ∘C, 5% CO_2_, and at a concentration of 1×10^6^ cells/mL in serum-free, X-VIVO-20 medium (Lonza BioWhittaker) with T cell activation beads coated with anti-CD2/CD3/CD28 antibodies (Miltenyi Biotec) alone or in combination with four different sets of recombinant human inflammatory mediators (i.e., five different culture conditions). The cells were then harvested from the culture medium for cell-hashing [36] and CITE-seq [35] single cell sequencing library preparation following the CITE-seq and hashing protocol available at https://cite-seq.com. The mRNA-, HTO-, and ADT-derived libraries were then pooled at approximately 85%, 5%, and 10% proportions, respectively, and the pool of these sequencing libraries was sent for 150-bp paired-end sequencing in two lanes of an Illumina HiSeq sequencer (MedGenome, Inc.).

All subjects were given informed consent, and the study is approved by the University of Pittsburgh IRB.

#### Simulation dataset

We also generated a simulated dataset by augmenting the PBMC-1 dataset. Specifically, we classify GEMs in the PBMC-1 dataset using both GMM-Demux and the heuristic classifier of Seurat. Then, we extract GEMs that are classified as SSDs by both classifiers. We recovered SSDs from all four samples. We assume these GEMs are SSDs in truth. A summary of SSDs from the four samples is provided in Table 3. Notice that the mean of the sample-labeling HTO count of sample 1 (HTO 1) is significantly larger than the other three samples (HTO 2 in sample 2, HTO 3 in sample 3, and HTO 4 in sample 4). This shows that the sample barcoding could be susceptible to experimental inconsistencies and may include inconsistent levels of HTO counts among samples.

**Table 3:** Per-sample HTO antibody means and standard deviations of SSDs in the PBMC-1 dataset

		HTO 1	HTO 2	HTO 3	HTO 4
Sample 1	Mean	2789.10	20.94	38.99	17.34
	Std	1637.15	11.85	18.33	9.94
Sample 2	Mean	76.91	831.75	36.06	15.86
	Std	43.92	680.13	17.56	10.09
Sample 3	Mean	77.66	19.92	1117.05	16.12
	Std	43.16	12.04	783.55	10.23
Sample 4	Mean	75.56	19.33	36.25	717.48
	Std	43.34	11.66	18.22	457.40

We used the extracted SSDs to generate a batch of simulated datasets covering a wide range of possible sample barcoding scenarios, including varying number of samples for barcoding, varying MSM percentages, and varying degrees of population imbalances between samples. For each dataset, we randomly distribute the SSDs into droplets. If a droplet is assigned with a single SSD, then it inherits the HTO counts of that SSD. If a droplet is assigned with more than one SSD, then the new HTO counts of the droplet are computed by adding the HTO counts of its assigned SSDs together. Let *j* denote a simulated multi-SSD droplet and $\mathbb {SSD}_{j}$ denote the set of SSDs assigned to *j*, we compute the new HTO counts of *j* as ${\bar {x}}_{j} = \sum _{i \in \mathbb {SSD}_{j}} w_{i} \cdot {x}_{i}$, where *w*_*i*_ is a random weight generated from $\mathcal {N}(\mu = 1,\,\sigma ^{2}=0.04)$ and *x*_*i*_ is the HTO count vector of SSD *i*. Simulated multi-SSD droplets that contain SSDs from multiple samples are marked as MSMs in ground truth.

We generated three sets of simulated datasets. In the first set, we generated datasets using different numbers of samples (2, 3, and 4 samples) while maintaining a fixed MSM percentage at 10% and equal SSD populations among samples. In the second set, we used all four samples with equal populations and generated simulated datasets with different MSM percentages (5%, 10%, and 15%). In the third set, we selected three samples (sample 1, sample 2, and sample 3), fixed the MSM percentage at 10%, and downsized sample populations into geometric sequences. We generated three datasets with common ratios of 1, $\frac {1}{2}$, and $\frac {1}{3}$, respectively. A summary of all nine simulation datasets is provided in Table 4.

**Table 4:** Simulation configurations

Dataset names	MSM percentage (%)	Input SSD samples	Sample cell ratio
2 samples	10	Sample 1, sample 2	1:1
3 samples	10	Sample 1, sample 2, sample 3	1:1:1
4 samples	10	Sample 1, sample 2, sample 3, sample 4	1:1:1:1
5% MSM	5	Sample 1, sample 2, sample 3, sample 4	1:1:1:1
10% MSM	10	Sample 1, sample 2, sample 3, sample 4	1:1:1:1
15% MSM	15	Sample 1, sample 2, sample 3, sample 4	1:1:1:1
1 × scale	10	Sample 1, sample 2, sample 3	1:1:1
2 ×	10	Sample 1, sample 2, sample 3	4:2:1
3 ×	10	Sample 1, sample 2, sample 3	9:3:1

### Multi-sample multiplet classification results

For each cell-hashing dataset, we compare the MSM classification results of five MSM classifiers: the GMM-Demux classifier, the heuristic classifier of Seurat, the heuristic classifier of MULTI-seq, the model-based classifier demuxEM, and a human-supervised classifier. For the human-supervised classifier, a trained laboratory technician classifies GEMs based on the CLR-transformed HTO matrix.

The classification results are visualized in 2D tSNE plots [16]. The tSNE plots are generated directly from the HTO matrix. Note that the tSNE transformation is probabilistic and non-deterministic: GEMs with similar HTO UMI count profiles are likely to be grouped together, but there is no guarantee [42]. Sometimes, a small fraction of GEMs are incorrectly clustered with dissimilar neighbors, due to inaccuracies of the tSNE transformation. We use tSNE plots only for visualization and do not expect it to 100% reflect the truth.

#### Classification results on real datasets

**Fig. 3:**
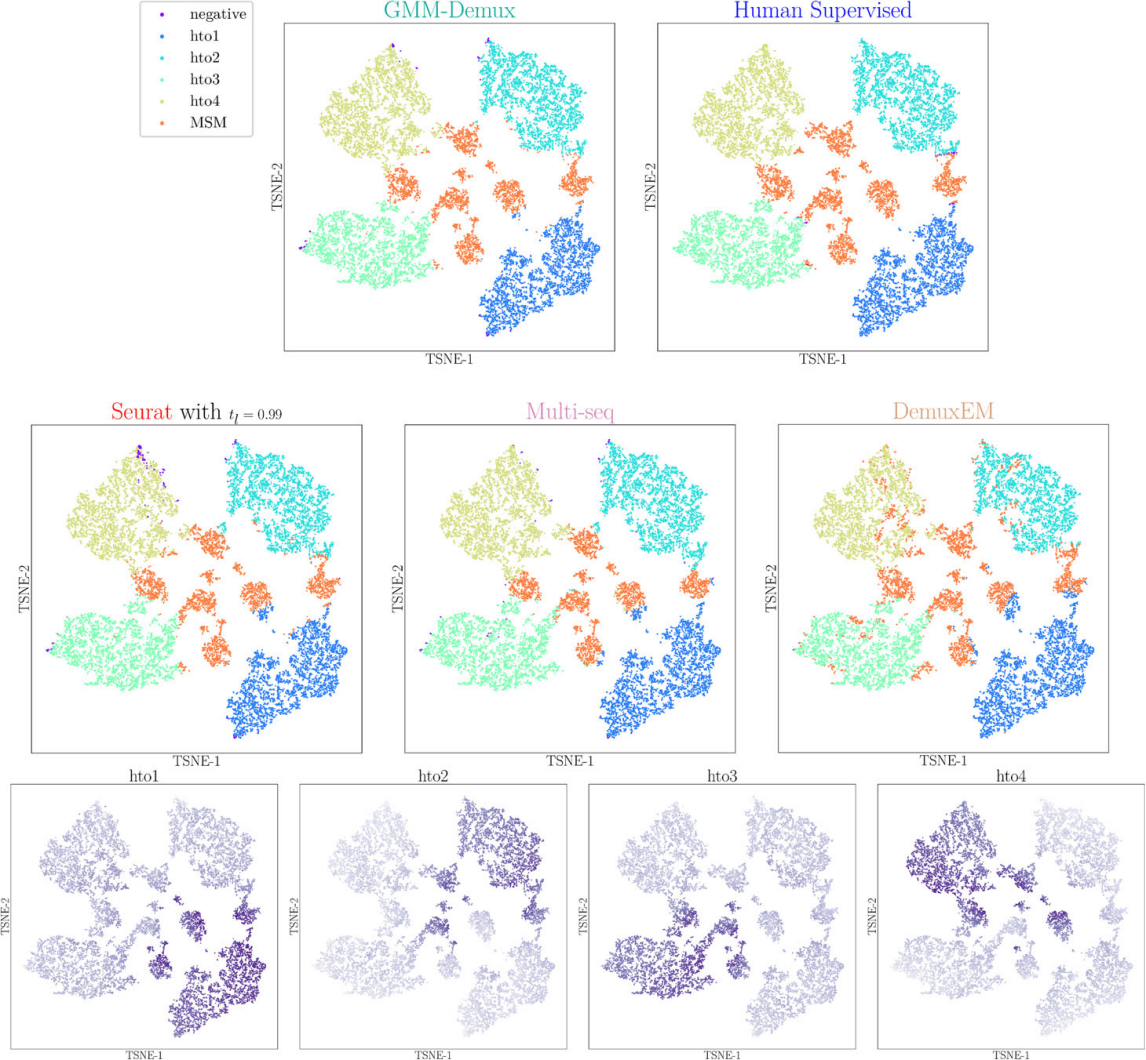
Classification results of the PBMC-1 dataset. Each dot represents a cell. The upper panels present classification results produced by the five classifiers. The lower panel stores the heat maps of HTO UMI counts of individual samples

The classification results of the PBMC-1 dataset are shown in Fig. 3. Shown in the top panel are the GMM-Demux classification result, the human-supervised classification result, the Seurat classification result, the MULTI-seq classification result, and the demuxEM classification result, and a set of HTO UMI count heat maps of individual samples in the bottom panel. In each heat map, GEMs with higher HTO UMI counts of the sample have darker colors. For simplicity, we lump all MSMs together as a single class—the MSM class, while maintaining SSDs of different samples as separate classes. Additional classification results for the PBMC-2 and the Memory T datasets are provided in Additional file 1: Fig. S3. If needed, GMM-Demux is able to subdivide MSMs into sub-classes where each sample combination is given a distinct class. Distinct MSM classification results are provided in Additional file 1: Fig. S4.

Figure 3 shows that the classification results from all five classifiers are mostly consistent. We compare the classification results against the HTO UMI count heat maps: a correct SSD classification should have a dark color in a single heat map and light colors in the rest of the heat maps; a correct MSM classification should have dark colors in more than one heat map. As evident in Fig. 3, the heat maps reinforce the MSM classifications by GMM-Demux.

**Fig. 4:**
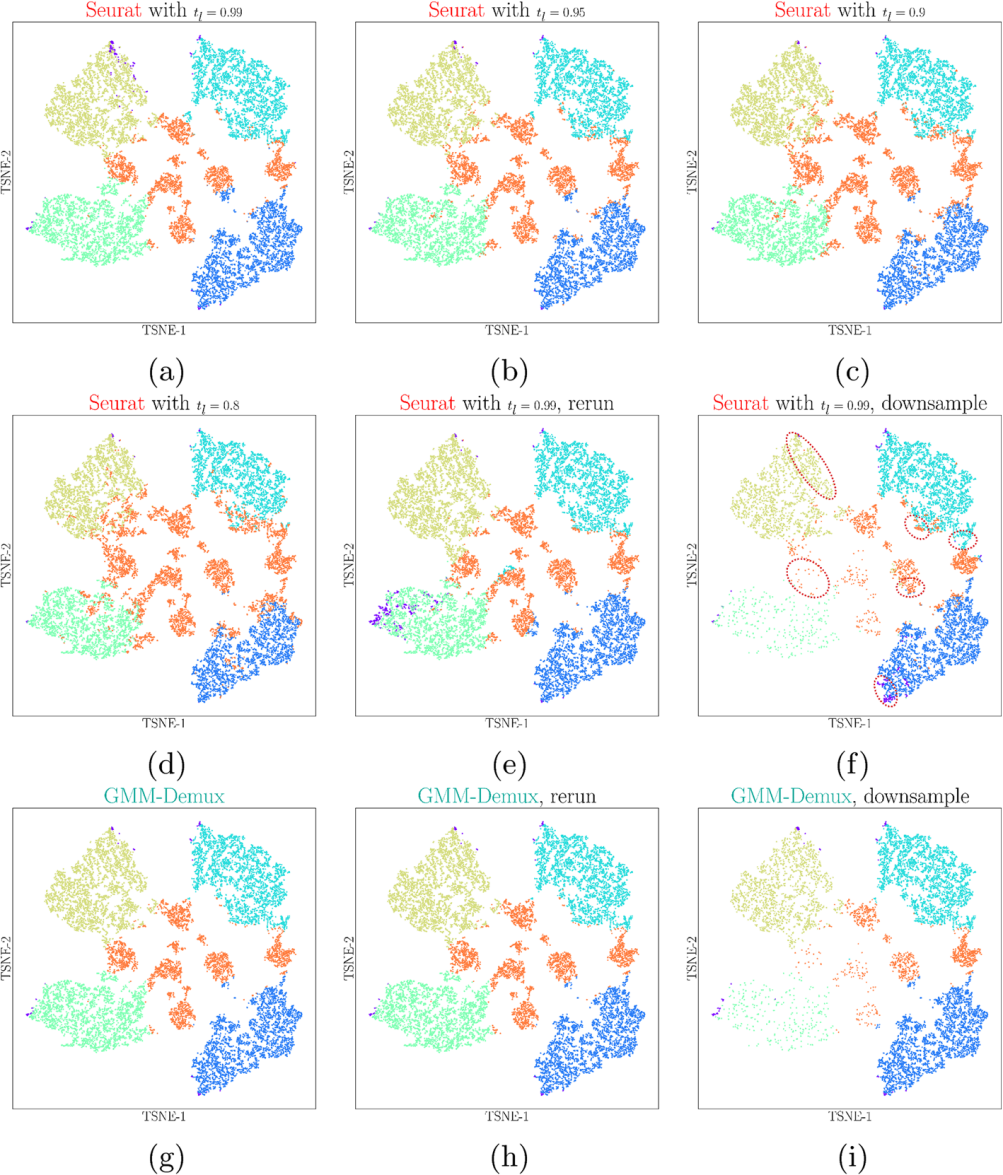
Stability test results. The Seurat classifier produces different classification results with regard to varying Seurat parameters, *t*_*l*_. It also generates inconsistent classification results during repetitive executions and is susceptible to data augmentation (sub-sampling). Classification differences of the Seurat classifier are highlighted in red-dotted circles. GMM-Demux, on the contrary, generates consistent classification results

Even though Seurat generates classification results similar to those produced with the GMM-Demux classifier, it is heuristic-based and unstable. Figure 4 illustrates the heuristic and unstable nature of the Seurat classifier. Results in this figure are generated from the PBMC-1 dataset. Since the heuristic classifier relies on the HTO UMI count threshold for classification, which is indirectly controlled by *t*_*l*_, it generates different classification results with different *t*_*l*_ values, as shown in Fig. 4a–d. From the figures, we observe that while a smaller *t*_*l*_ produces fewer negative classifications, it generates more MSM classifications. This is expected as a smaller *t*_*l*_ reduces the HTO UMI count threshold, which in turn increases the number of cell-enclosing GEMs in each sample. Without ground truth, however, it is not obvious which *t*_*l*_ provides the most accurate classification result. Such high variations in the classification results, as well as the heavy reliance on heuristic parameters, reduce the reliability of the Seurat classifier. In practice, it is difficult to select the appropriate *t*_*l*_ for the best accuracy.

On top of its heuristic nature, because it uses the non-deterministic *K*-medoid clustering algorithm, the Seurat classifier generates different results between two runs even with the same heuristic parameter. This is visualized by comparing Fig. 4a against e. Both figures are generated under *t*_*l*_=0.99. Differences between them (highlighted in red-dotted circles) stem solely from the non-determinism of the *K*-medoid algorithm.

Finally, the Seurat classifier is highly sensitive to changes in the dataset. In Fig. 4f, we randomly sub-sample GEMs from samples 3 and 4 (by 10% and 50%, respectively). When compared against Fig. 4a, we observe substantial changes in the classification result, highlighted in red-dotted circles. This is because as the sample composition changes, the HTO count threshold of each sample also changes, even without updating *t*_*l*_. As a result, previously classified MSMs now become SSDs and vice versa.

The GMM-Demux classifier, on the other hand, is model-based, stable, and far more deterministic. The GMM-Demux classifier does not require heuristic parameters for MSM classification and generates consistent classification results across repetitive runs. Despite of uncertainties introduced by the EM algorithm, because GMM-Demux is model-based and the HTO UMI count distributions possess obvious features of a 2-component Gaussian mixture, the EM algorithm always converges. Hence, GMM-Demux generates consistent results. Figure 4g and 4h show the classification results of two repetitive runs of GMM-Demux. There exist little differences between the two figures. Similarly, the GMM-Demux classifier is much less susceptible to sub-sampling, as shown in Fig. 4i, where we sub-sampled GEMs from samples 3 and 4, as we did in Fig. 4f. By comparing Fig. 4i against g, we observe minimal changes in GEM classifications. A more detailed stability analysis across all four sample barcoding classifiers is included in Additional file 1: Fig. S5.

Not all GEMs can be confidently classified by GMM-Demux. Some GEMs have low HTO UMI counts across all samples, while other GEMs have similar probabilities between multiple classes (such as between a *l*_1_ SSD and a *l*_1_∩*l*_2_ MSM). Neither type of GEMs can be well classified: the former are classified as *negative* GEMs, which should be experimental errors, while the latter are classified as *unclear* GEMs, which are too ambiguous to be included in the final result. GMM-Demux lets the user specify the confidence threshold, *c*, such that the user can customize the removal of unclear GEMs: a low confidence threshold salvages more unclear GEMs in the final result at the expense of decreased MSM classification quality. Across all three cell-hashing datasets, over 99% of GEMs have confidence scores above 0.8. Therefore, we set the default confidence threshold of GMM-Demux at 0.8 (*c*=0.8). Detailed distributions of confidence scores are provided in Additional file 1: Fig. S14.

#### Classification results on the simulation datasets

**Fig. 5:**
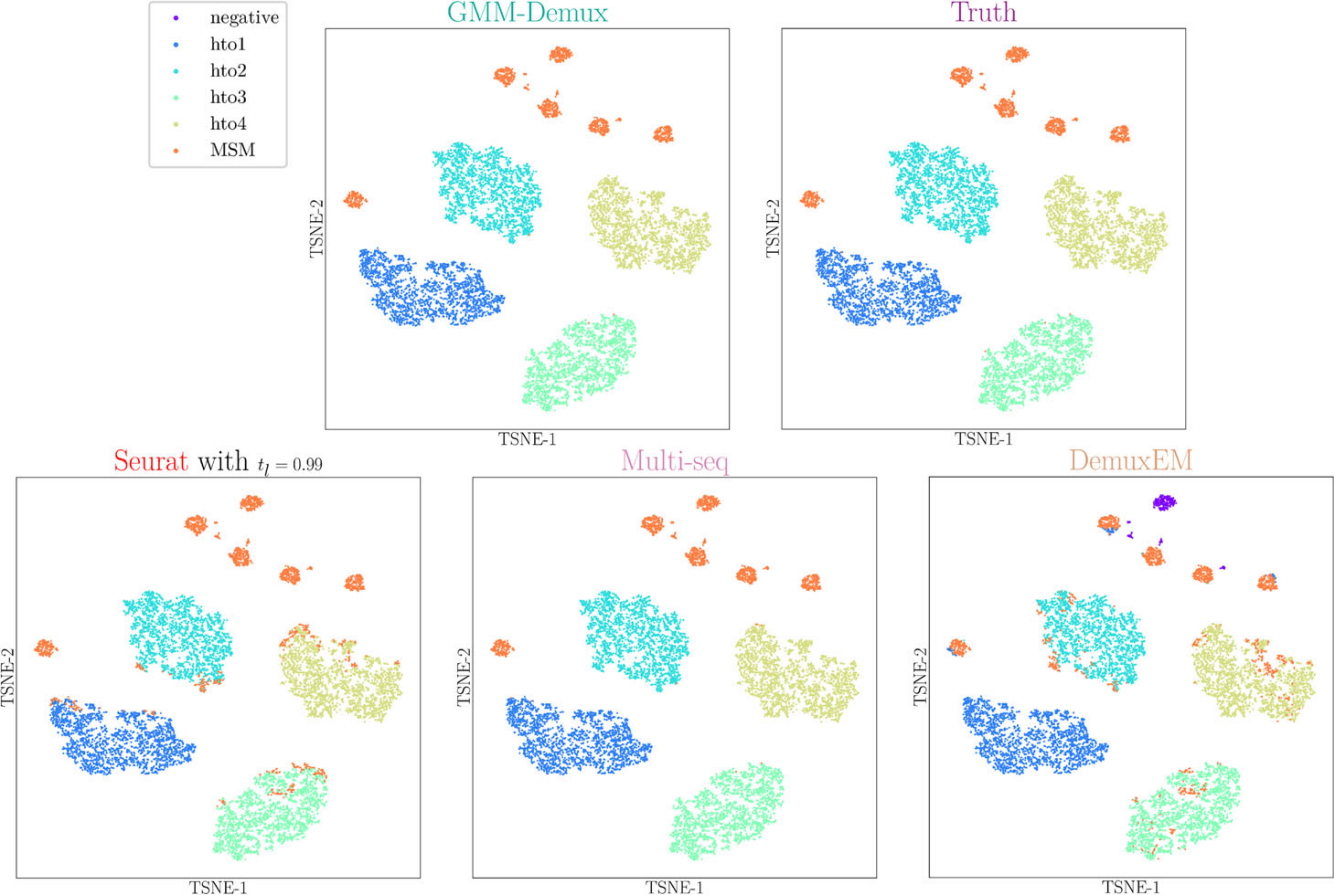
Classification results of the 4-sample simulated dataset. GMM-Demux and MULTI-seq produce classifications results that are most in accordance with the ground truth

We benchmark the accuracy of GMM-Demux against the other three classifiers (Seurat, MULTI-seq, and demuxEM) by applying all four methods to the 9 simulation datasets and compare their classification results against the ground truth. All classifiers are benchmarked with their default parameters and are repeated 20 times for each dataset. An example set of classification results of the 4-sample simulation dataset is visualized in Fig. 5.

**Fig. 6:**
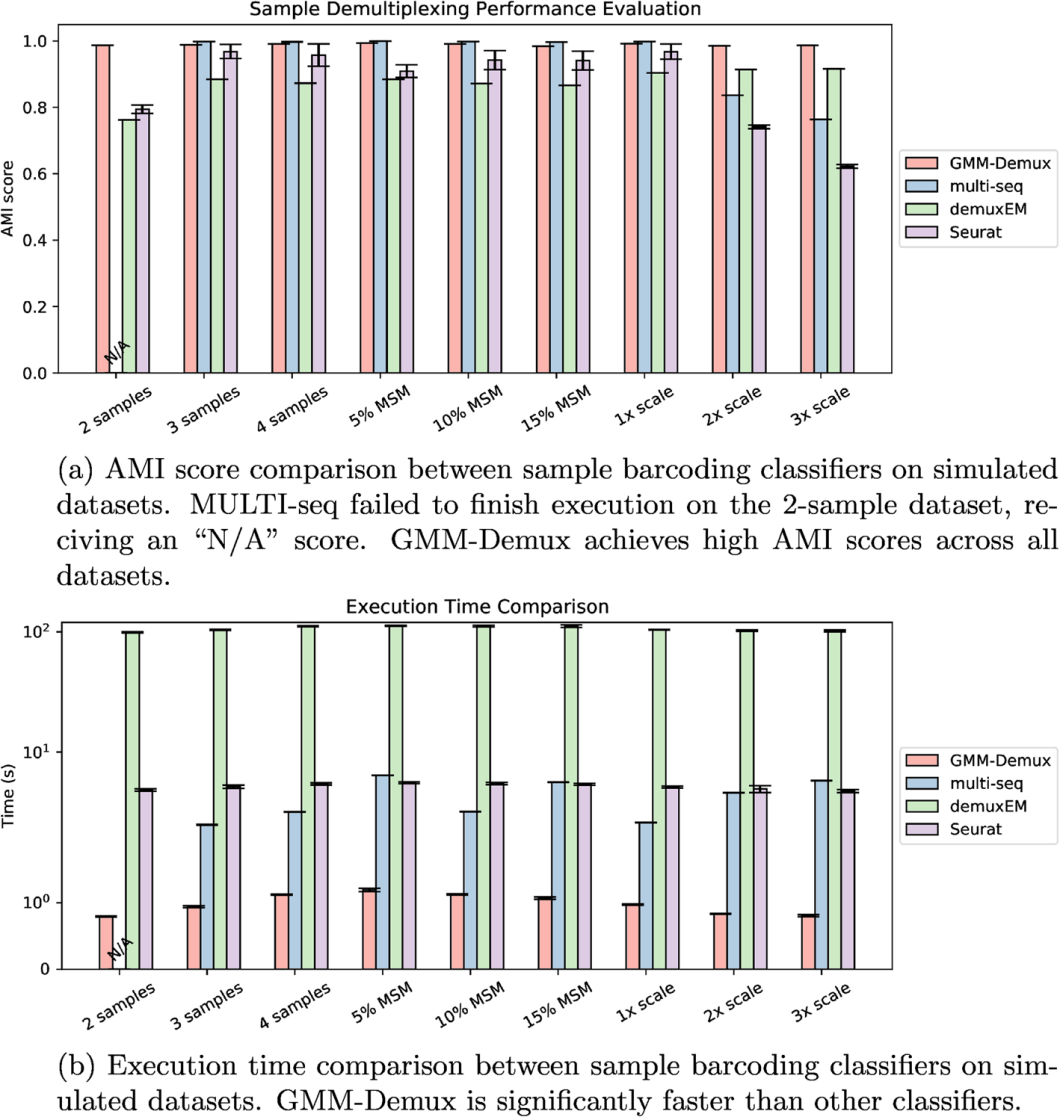
Comparison of MSM classifiers on simulated datasets

For each classification, we compute the Adjusted Mutual Information (AMI) score between itself and the ground truth. The AMI score comparison across all simulation datasets is provided in Fig. 6a. As shown in the figure, GMM-Demux achieves high classification accuracies across all scenarios, whereas other sample barcoding classifiers have faltered accuracy under low sample numbers (2 samples) or high sample imbalances (2 × scale and 3 × scale). In particular, MULTI-seq failed to derive a stable quantile HTO count cutoff for the 2-sample dataset and cannot converge to a stable classification solution. A detailed explanation of why MULTI-seq fails is provided in the “Related works” section. Figure 6a proves that GMM-Demux is highly accurate and is the most stable sample barcoding classifier.

Figure 6b records the execution time of each classifier over all simulated datasets. As shown in the figure, GMM-Demux is significantly faster than other sample barcoding classifiers.

### Same-sample multiplet rate estimation results

We prove the correctness of the SSM estimator indirectly by validating the GEM formation model. Even though the SSM rate truth is not directly observable, if the underlying probabilistic model is accurate, then the SSM rates derived from the model should also be trustworthy. For this purpose, we compare the model-derived MSM rates against the GMM-Demux classifier-observed MSM rates. If the numbers match, then we claim the GEM formation model must accurately characterize the GEM formation process.

**Fig. 7:**
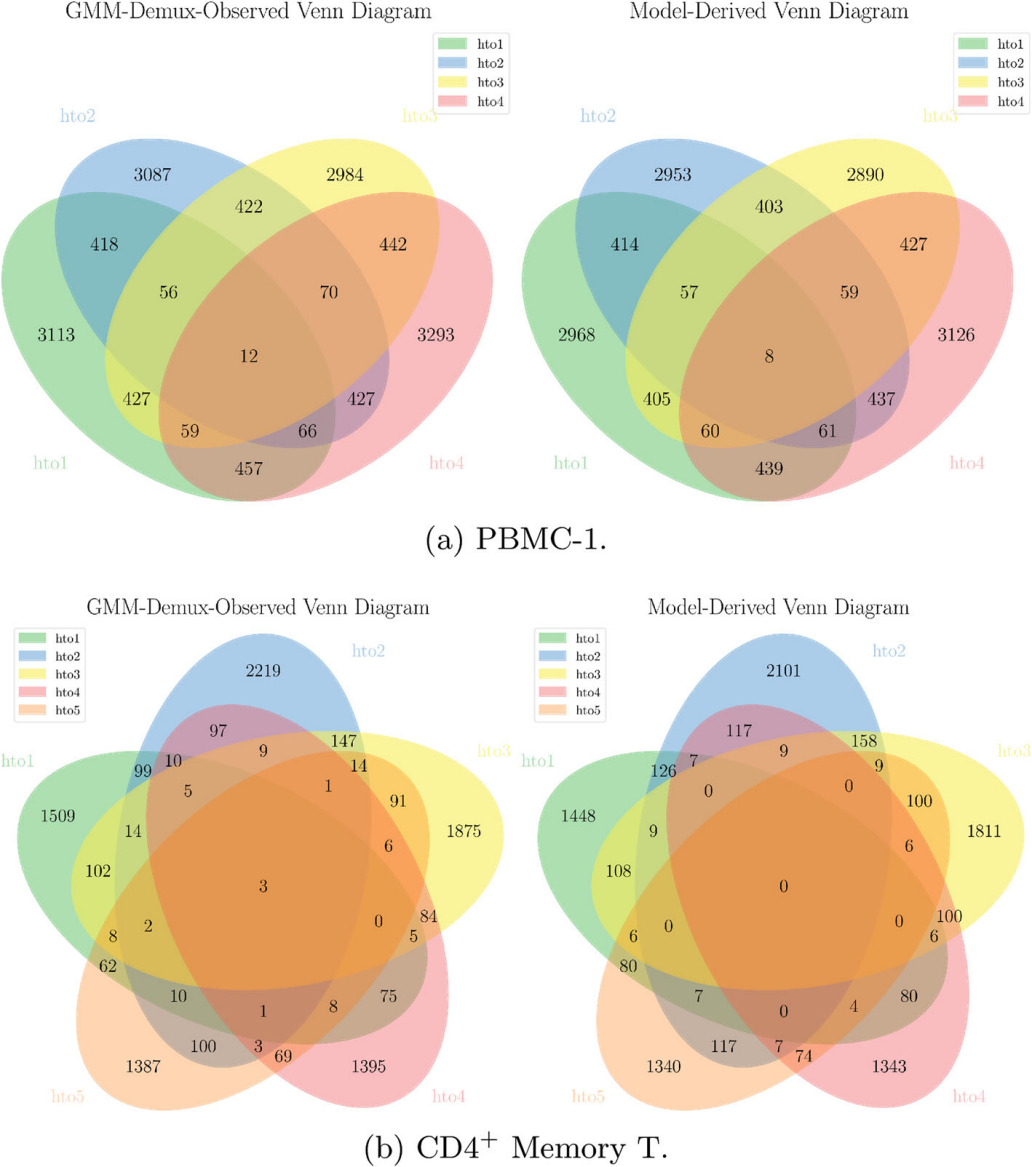
Comparison of model-derived Venn diagrams against GMM-Demux-observed Venn diagrams. Values in the model-derived Venn diagrams are consistent with values in the GMM-Demux-observed Venn diagrams, thus proving the correctness of the GEM formation model

For comprehensiveness, we compare not only the overall MSM rates of a dataset, but also the MSM rates of individual sample combinations. For each sample combination, we compare the model-derived MSM UMI count against the MSM classifier-observed UMI count. The comparison results are summarized into Venn diagrams, which illustrate the number of SSDs of each sample as well as the number of MSMs of each sample combination. We compare the model-derived Venn diagram against the MSM classifier-observed Venn diagram. Figure 7 includes the Venn diagram comparisons of the PBMC-1 and the CD4^+^ Memory T datasets. Comparison of the PBMC-2 dataset is included in the table of Additional file 2 (its per-sample combination classification result cannot be visualized in a Venn diagram due to a large number of sample combinations).

From Fig. 7, we observe that the model-derived MSM counts are consistent with the observed values from the MSM classifier. Therefore, we prove that the droplet formation model is accurate.

The estimated number of droplets (*X*) and the model-estimated singlet, MSM (Est. MSM), SSM, and relative SSM (RSSM) rates of each sample are summarized in Table 5. Also included in Table 5 are the GMM-Demux classifier-observed MSM rates (Obs. MSM) and the proportions of unclear GEMs (GEMs with confidence scores below *c*=0.8) and negative GEMs in each dataset. Except the number of droplets (*X*), all rates are presented as percentiles (%). As shown in the table, the model-derived MSM rates are generally consistent with the classifier-observed MSM rates.

**Table 5:** Summary of classification results across all datasets. All values except the number of droplets (*X*) are presented in percentages (%)

Dataset	No of droplets (*X*)	*r*_*cap*_	Singlet	Est. MSM	Obs. MSM	SSM	RSSM	Negative	Unclear
PBMC-1	68,480	56	76.11	18.64	18.05	5.25	6.45	0.47	2.71
Memory T	78,413	44	86.17	10.89	10.57	2.93	3.29	0.67	2.31
PBMC-2	77,663	63.5	82.96	15.11	14.55	1.93	2.28	0.79	2.88

A detailed introduction of the droplet formation model-based online experiment planner is provided in Additional file 1: Section S8. A suite of profiling results produced by the online experiment planner under varying experimental settings is provided in Additional file 1: Section S9.

### Cell-type authentication results

#### Cell-type authentication via joint analysis with surface marker data

**Fig. 8:**
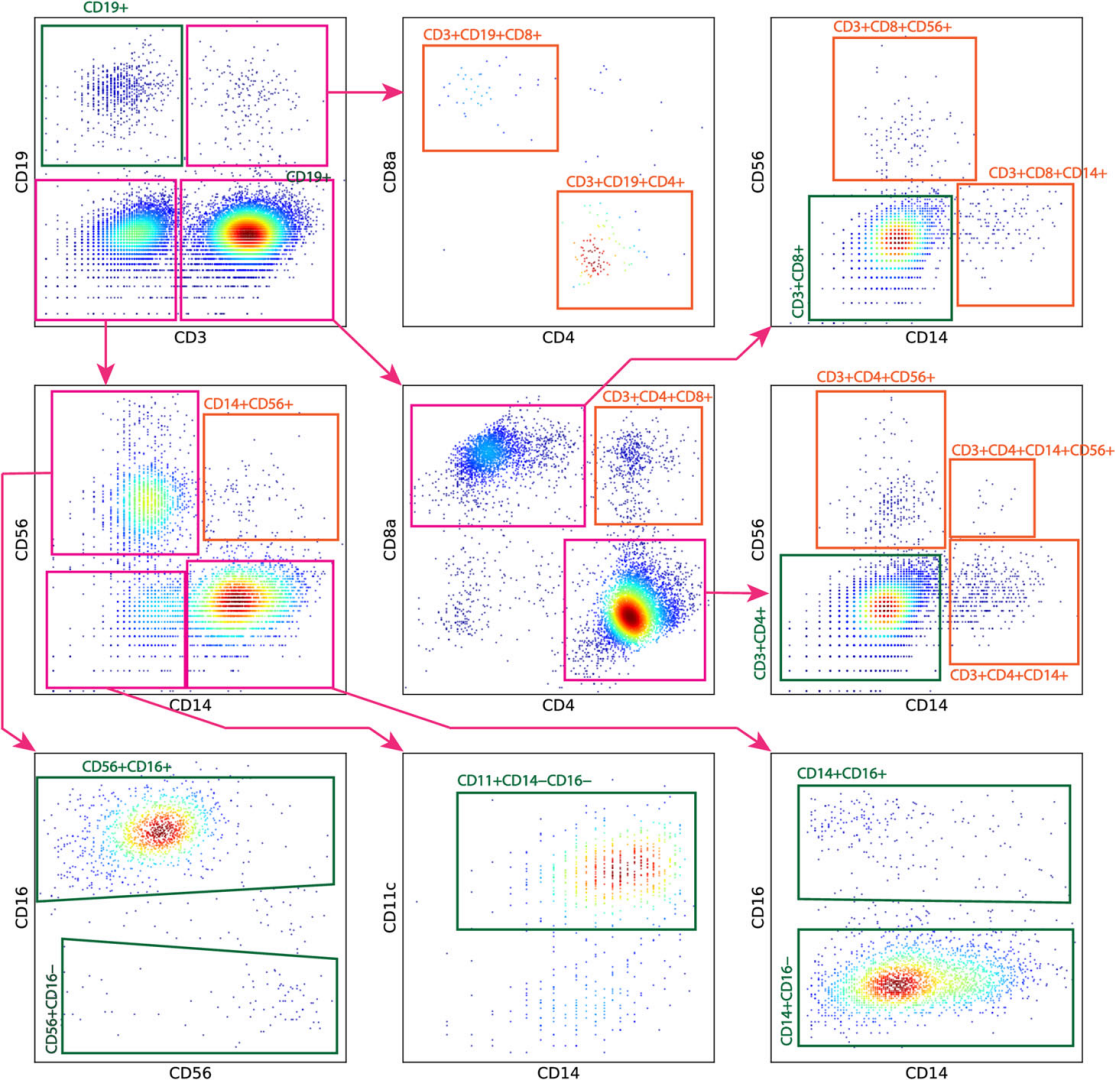
The manual gating strategy applied for cell-type annotation in PBMC-1, using its surface marker expression data. In general, we follow the gating strategy outlined in Maecker et al. [19]. We first gate GEMs on CD3 and CD19. CD3^+^ GEMs are further gated over CD4 and CD8. CD3^-^CD19^-^ GEMs are further gated over CD14 and CD56. For GEM clusters gated from the CD14-CD56 penal, the CD14^+^ and CD56^+^ GEMs are further gated over CD16. The CD14^-^CD56^-^ GEMs are gated over CD11c to extract CD11c^+^ DC GEMs. The 8 cell types commonly observed in PBMCs are highlighted in green bounding boxes. Some GEM clusters, such as the CD3^+^CD4^+^ GEM cluster, go through additional gating, in order to reveal non-conventional sub-clusters in PBMCs (which are later classified as phony-type GEM clusters), such as the CD3^+^CD4^+^CD14^+^ GEM cluster or the CD3^+^CD4^+^CD56^+^ GEM cluster. Non-conventional GEM clusters are highlighted in orange bounding boxes

GEMs in the PBMC-1 dataset are manually assigned into 17 distinctive clusters following the gating strategy detailed in Maecker et al. [19], which is visualized in Fig. 8. Among the 17 GEM clusters, 8 of them represent well-characterized cell types found in PBMCs (highlighted in green bounding boxes); 9 of them are rarely observed in PBMCs and are labeled as putative novel cell-type candidates (highlighted in orange bounding boxes). All GEM clusters, annotated by their defining surface markers and their inferred cell types, if available, are summarized in Table 6.

**Table 6:** Summary of the 17 GEM clusters manually gated from PBMC-1. Given the cell-hashing configuration, the minimum MSM percentage of a phony-type GEM cluster in PBMC-1 is 74.98%. Pure-type GEM clusters have variate MSM rates depending on their size. Among the 17 manually gated GEM clusters, 9 have MSM percentages approaching and exceeding 74.98% and are classified as phony-type GEM clusters, 6 have MSM percentages of pure-type GEM clusters and are classified as pure-type GEM clusters, and 2 have MSM percentages of neither pure-type nor phony-type GEM clusters and are classified as mixture clusters

Cell type	MSM %	MSM %	MSM %	*p* value	*p* value	Cluster
	(observed)	(phony)	(pure)	(phony)	(pure)	classification
CD19^+^ (B cells)	6.47	74.98	5.93	0	0.29	Pure
CD3^+^CD4^+^ (helper T cells)	11.38	74.98	11.36	0	0.49	Pure
CD3^+^CD8^+^ (cytotoxic T cells)	7.33	74.98	7.52	0	0.68	Pure
CD14^+^CD16^-^ (classical monocytes)	7.00	74.98	7.49	0	0.88	Pure
CD14^+^CD16^+^ (non-classical monocytes)	14.91	74.98	5.82	1.75e−175	2.26e−13	Mixture
CD56^+^CD16^-^ (CD16^-^ NK cells)	6.20	74.98	6.41	8.70e−113	0.64	Pure
CD56^+^CD16^+^ (CD16^+^ NK cells)	9.62	74.98	6.72	0	1.00e−07	Mixture
CD11^+^CD14^-^CD16^-^ (DCs)	7.30	74.98	5.86	1.01e−149	0.20	Pure
CD14^+^CD56^+^	76.56	74.98	6.90	0.62	5.24e−77	Phony
CD3^+^CD4^+^CD14^+^	74.67	74.98	6.22	0.42	0	Phony
CD3^+^CD4^+^CD19^+^	74.04	74.98	6.88	0.41	3.44e−119	Phony
CD3^+^CD4^+^CD56^+^	73.38	74.98	5.73	0.21	0	Phony
CD3^+^CD4^+^CD8^+^	75.55	74.98	6.02	0.66	0	Phony
CD3^+^CD8^+^CD14^+^	73.24	74.98	6.23	0.27	1.18e−165	Phony
CD3^+^CD8^+^CD19^+^	73.81	74.98	9.21	0.44	1.64e−41	Phony
CD3^+^CD8^+^CD56^+^	75.47	74.98	8.33	0.54	6.64e−57	Phony
CD3^+^CD4^+^CD14^+^CD56^+^	84.62	74.98	13.04	0.86	8.13e−14	Phony

For each GEM cluster, GMM-Demux computes the MSM percentage of the cluster and compares it against the anticipated pure-type MSM percentage as well as the anticipated phony-type MSM percentage of the cluster. The anticipated pure-type MSM percentage of the cluster is a hypothetical value derived from the GEM formation model by assuming that the GEM cluster represents a real cell type. Similarly, the anticipated phony-type GEM percentage is computed by assuming the GEM cluster is a phony-type GEM cluster. Based on the observed and anticipated MSM percentages, GMM-Demux performs pure-type and phony-type hypothesis testings and classifies the GEM cluster according to the *p* values of both tests. The classification results, as well as the intermediate results in classifying each GEM cluster, are also included in Table 6. As summarized in Table 6, the PBMC-1 dataset contains 9 cell types rarely observed in PBMCs. Named after their defining surface markers, these are as follows:

- CD14^+^CD56^+^
- CD3^+^CD4^+^CD14^+^
- CD3^+^CD4^+^CD19^+^
- CD3^+^CD4^+^CD56^+^
- CD3^+^CD4^+^CD8^+^
- CD3^+^CD8^+^CD14^+^
- CD3^+^CD8^+^CD19^+^
- CD3^+^CD8^+^CD56^+^
- CD3^+^CD4^+^CD14^+^CD56^+^

Upon further investigation, we observe that all 9 putative novel-cell-type-defining GEM clusters have very high MSM percentages, approaching and exceeding their anticipated phony-type MSM percentages. When tested with pure-type hypothesis, all 9 clusters have extremely small *p* values; and large *p* values from phony-type hypothesis tests. Consequently, GMM-Demux designates all 9 GEM clusters as phony-type clusters.

Such result suggests that all 9 GEM clusters contain multiplets of different cell types. For instance, the CD14^+^CD56^+^ GEM cluster contains multiplets that include both monocyte cells (CD14^+^) and NK cells (CD56^+^). Among the 9 phony-type GEM clusters, the CD3^+^CD4^+^CD14^+^CD56^+^ GEM cluster has the largest MSM percentage, significantly larger than the rest. With further examination of its defining surface markers, we conclude that it contains triple-type GEMs—GEMs that include CD3^+^CD4^+^ T cells, CD14^+^ monocytes, and CD56^+^ NK cells. According to the GEM formation model for phony-type hypothesis testing, detailed in Additional file 1: Section S3, triple-type phony GEM clusters have higher MSM percentages than double-type phony GEM clusters. This explains the larger MSM percentage of the CD3^+^CD4^+^CD14^+^CD56^+^ GEM cluster.

For the remaining 8 GEM clusters, which represent well-characterized cell types in PBMCs, 6 of them are classified as pure-type GEM clusters, with the exception of the CD14^+^CD16^+^ non-classical monocyte GEM cluster and the CD56^+^CD16^+^ NK GEM cluster. Both clusters are classified as mixture GEM clusters, suggesting that they contain both pure-type and phony-type GEMs. This classification result is reasonable, as both GEM clusters contain fractions of indistinguishable multiplets. For instance, inside the CD14^+^CD16^+^ GEM cluster, there could be a small fraction of CD14^+^CD16^+^-and-CD14^+^CD16^-^ phony-type GEMs. These phony-type GEMs are CD14^+^CD16^+^-and-CD14^+^CD16^-^ the CD14^+^CD16^+^ pure-type GEMs in gating. In gating, boundaries between cell types are drawn in a log-transformed surface marker space. After log transformation, the surface marker expression profile of a CD14^+^CD16^+^-and-CD14^+^CD16^-^ phony-type GEM is almost identical to a CD14^+^CD16^+^ pure-type GEM, even if they contain the same CD14^+^CD16^+^ non-classical monocyte cell. The only difference: the CD14^+^CD16^+^-and-CD14^+^CD16^-^ phony-type GEM is likely to have a slightly larger log-transformed CD14 expression value. Such subtle differences do not warrant the separation of CD14^+^CD16^+^-and-CD14^+^CD16^-^ phony-type GEMs from CD14^+^CD16^+^ pure-type GEMs. Due to intrinsic variations in surface marker expression levels, the two types of GEMs intermix with each other into a single, indivisible GEM cluster. Similarly, CD56^+^CD16^+^-and-CD56^+^CD16^-^ phony-type GEMs are also indistinguishable from CD56^+^CD16^+^ pure-type GEMs. This explains the slightly-higher-than-expected MSM percentages in the CD14^+^CD16^+^ monocyte GEM cluster and the CD56^+^CD16^+^ NK GEM cluster, which resulted in designating them as mixture GEM clusters. Nonetheless, these should be the only phony-type GEMs they contain. Therefore, the MSM percentages of both clusters are only moderately above their corresponding pure-type MSM percentages, remaining significantly smaller than their corresponding phony-type-qualifying MSM percentage thresholds, reflecting that both clusters still have a pure-type GEM majority. Overall, we conclude that the 8 GEM clusters with low MSM percentages represent real cell types in PBMC, in concordance with previous knowledge on PBMCs [19].

To validate the classification results of GMM-Demux, we conducted an additional CITE-seq sequencing experiment over a PBMC sample from the same donor of PBMC-1. The additional CITE-seq experiment measures the same set of surface markers as in PBMC-1. To control the percentage of multiplets, we loaded only 3.2K cells while harvesting ∼ 1.6K GEMs. The online experiment planner estimated percentage of multiplets of this dataset is 1.9%, compared to 23.9% in PBMC-1. We sorted GEMs following the same gating strategy illustrated in Fig. 8. Table 7 records the percentages of the 17 manually gated cell types in both PBMC-1 and the validation dataset. We observe that all 9 phony-type GEM clusters identified in PBMC-1 have much-reduced, close-to-zero presence in the validation dataset, while the 8 pure-type GEM clusters have similar footprints. This confirms the classification results of GMM-Demux.

**Table 7:** Percentages of the 17 cell types in both PBMC-1 and the validation dataset. All phony-type GEM clusters identified in PBMC-1 have close-to-zero presence in the validation dataset, suggesting that these cell types do not really exist and are artifacts of multiplets

Cell type	PBMC-1 (%)	Validation PBMC (%)
CD19^+^ (B cells)	2.72	5.31
CD3^+^CD4^+^ (helper T cells)	37.84	40.03
CD3^+^CD8^+^ (cytotoxic T cells)	12.81	16.22
CD14^+^CD16^-^ (classical monocytes)	12.85	8.79
CD14^+^CD16^+^ (non-classical monocytes)	1.79	0.76
CD56^+^CD16^-^ (CD16^-^ NK cells)	0.84	1.06
CD56^+^CD16^+^ (CD16^+^ NK cells)	8.41	14.60
CD11^+^CD14^-^CD16^-^ (DCs)	1.16	1.06
CD14^+^CD56^+^	0.42	0.08
CD3^+^CD4^+^CD14^+^	2.93	0.00
CD3^+^CD4^+^CD19^+^	0.68	0.08
CD3^+^CD4^+^CD56^+^	1.81	0.00
CD3^+^CD4^+^CD8^+^	2.99	0.42
CD3^+^CD8^+^CD14^+^	0.93	0.15
CD3^+^CD8^+^CD19^+^	0.27	0.12
CD3^+^CD8^+^CD56^+^	0.35	0.08
CD3^+^CD4^+^CD14^+^CD56^+^	0.08	0.00

Table 7 proves that removing MSMs alone does not eliminate all multiplets. None of the phony GEM clusters has a MSM percentage of 100%. All phony GEM clusters have non-negligible fractions of SSMs, which cannot be revealed or removed through sample barcoding alone. After removing all phony-type GEM clusters, we estimate the RSSM rate of PBMC-1 is further reduced to 3.29%, from 6.45%.

#### Gating refinement and joint cell-type authentication with transcriptomic data

**Fig. 9:**
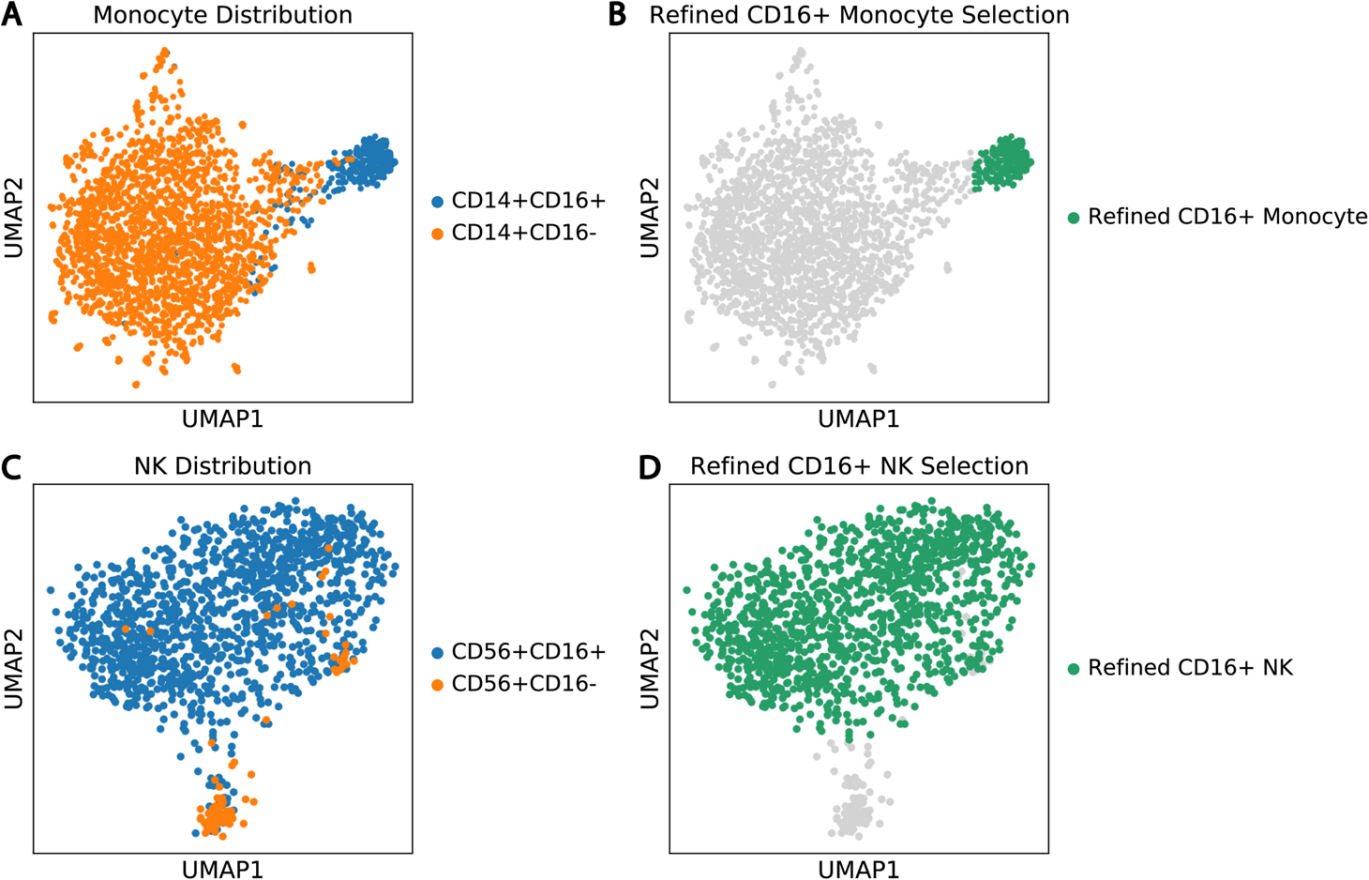
Refinement of the selection of CD16^+^ NK and monocyte GEMs using RNA expression data

The selection of the CD14^+^CD16^+^ non-classical monocyte cell and the CD56^+^CD16^+^ NK cells can be further refined with transcriptomic data. Figure 9a and 9b depict the previous surface marker-based classifications of monocyte and NK cells visualized in RNA UMAP plots, respectively. In both figures, we observe fractions of CD16^+^ cells disperse into the CD16^-^ cell groups. Following the assumption that phony-type GEMs inherit RNA profiles from both member cell types, we refine the selection of both CD16^+^ GEM groups by manually removing GEMs that disperse into the CD16^-^ GEM cluster. The refined cell selections are highlighted in Fig. 9c and 9d. After refinement, the MSM percentages of CD14^+^CD16^+^ and CD56^+^CD16^+^ GEM clusters reduce to 9.32% and 6.77%, respectively.

**Fig. 10:**
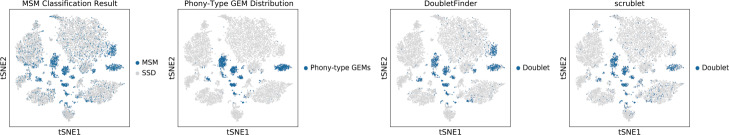
Comparison among the MSM distribution, the phony-type GEM distribution, and the doublet distributions identified by DoubletFinder and Scrublet, in surface marker-based tSNE plots

We also applied DoubletFinder and Scrublet to the PBMC-1 dataset. Figure 10 displays the GMM-Demux MSM classification result, the distribution of phony-type GEMs in Table 7, and the cross-cell-type multiplet identification results of DoubletFinder and Scrublet. Comparing the four plots, we observe that phony-type GEM clusters (Fig. 10b) have higher MSM concentrations (Fig. 10a) and house the majority of the cross-cell-type multiplets identified by DoubletFinder and Scrublet (Fig. 10c, d).

The DoubletFinder and Scrublet cross-cell-type multiplet identification results reinforce the putative cell-type authentication result of GMM-Demux. A detailed comparison of the MSM percentages and the DoubletFinder-identified doublet percentages of individual putative cell types is provided in Table 8. Putative cell types that have high MSM percentages also have high DoubletFinder-identified cross-cell-type multiplet percentages and vice versa. The concordance between the GMM-Demux authentication result and the RNA-based cross-cell-type multiplet identification results provides support for the correctness of GMM-Demux. Parameter selections for both DoubletFinder and Scrublet are included in Additional file 1: Section S12.

**Table 8:** GMM-Demux-identified MSM percentages and DoubletFinder-identified (DBF) doublet percentages of GEM clusters in Table 7

GEM type	MSM percentage (%)	DBF doublet percentage (%)
CD3+CD4+	11.39	4.67
CD3+CD8+	7.33	2.44
CD19+	6.47	0.00
CD14+CD16+	14.91	0.00
CD14+CD16-	7.00	0.05
CD56+CD16+	9.62	2.64
CD56+CD16-	6.20	0.00
CD11+CD14-CD16-	7.30	0.00
CD3+CD4+CD8+	75.55	52.84
CD3+CD4+CD14+	74.67	42.89
CD3+CD4+CD56+	73.38	47.48
CD3+CD4+CD19+	74.04	48.08
CD3+CD4+CD14+CD56+	84.62	84.62
CD3+CD8+CD19+	73.81	52.38
CD3+CD8+CD56+	75.47	41.51
CD3+CD8+CD14+	73.24	46.48
CD14+CD56+	76.56	48.44
CD56+CD16+ refined	9.32	2.76
CD14+CD16+ refined	6.77	0.00

Additional analysis on the impact of phony-type GEMs in downstream scRNA-seq analysis is provided in Additional file 1: Section S13. We show that phony-type GEMs can confound downstream analysis and degrade RNA clustering accuracy, as well as generating low-quality clusters with high MSM concentrations.

## Discussion

### Related works

Currently, there are three analytical methods for processing sample barcoding data: the heuristic classifier provided by Seurat (or simply Seurat), the heuristic classifier provided by MULTI-seq (or simply MULTI-seq), and the model-based classifier demuxEM. Seurat relies on the *K*-medoid clustering algorithm [11], a probabilistic method [31], to classify MSMs. Assuming there are a total of *M* samples, for each sample, it clusters all GEMs into *M* groups using the *K*-medoid clustering algorithm. Then, it removes the group with the highest mean, combines the remaining groups, fits the combined data with a negative binomial distribution, excludes the top 5% values as outliers, computes the *q*=*t*_*l*_ quantile (*t*_*l*_ is set to 99% by default) of the fitted distribution, and finally tags GEMs with HTO UMI values that are greater than *q* as sample-specific GEMs. If a GEM is classified as cell-enclosing in multiple samples, then Seurat brands it as a MSM.

While Seurat has sufficiently demonstrated the benefit of sample barcoding, it is heuristic-based and is unstable. It includes a number of arbitrary parameters. It does not explain why it fits the data with a negative binomial distribution as opposed to other distributions, nor does it explain why it removes the top 5% values as outliers or sets *t*_*l*_=99*%* as the default value. As we will see in the “Results” section, by setting *t*_*l*_ differently, it generates conflicting results and it is not evident which *t*_*l*_ provides the best result. Furthermore, because it relies on the *K*-medoid clustering algorithm, which generates inconsistent results over repetitive runs, Seurat also generates inconsistent classification results over repetitive executions.

MULTI-seq uses simple quantile cutoffs to classify GEMs. It assumes that the HTO antibody distributions across all samples have similar shapes. By design, MULTI-seq first finds the two maximums that correspond to the two peaks of the two Gaussian components in each HTO distribution (CLR-transformed), termed the on-target ($\mathcal {N}_{high}$) and the off-target ($\mathcal {N}_{low}$) maximums. It then sets a universal quantile HTO count cutoff between the two maximums across all barcodes: GEMs with HTO counts of a sample that exceed the quantile cutoff are classified as containing cells from that sample. GEMs which have HTO counts from a single sample exceeding the quantile cutoff are SSDs, GEMs that have HTO counts from multiple samples exceeding the quantile cutoff are MSMs, and GEMs that do not have any HTO count exceeding the quantile cutoff are negative GEMs. MULTI-seq sets the quantile cutoff in an iterative and heuristic manner: it finds a cutoff that yields the highest count of SSDs across all samples. Then, it classifies all droplets accordingly and removes all negative GEMs. It repeats the process until there is no negative droplet left. MULTI-seq performs a final, reclassification step which uses *K*-means to update the classification of some of the previously classified negative GEMs into SSDs.

The implementation of MULTI-seq, however, depends on an unreliable heuristic. Instead of finding the HTO values that correspond to the two peaks of the two Gaussian components in each HTO distribution, MULTI-seq generates an array of local maximums in each distribution and designates the maxima with the largest HTO count as the on-target maxima of the sample and the maxima that produces the highest peak in the distribution as the off-target maxima of the sample. In doing so, MULTI-seq implicitly assumes that there are always more off-target GEMs than on-target GEMs in each HTO distribution. In reality, when there are only two samples in a sample barcoding experiment, or when one sample has a larger population than the rest combined, then the above assumption of MULTI-seq no longer holds. In those cases, MULTI-seq is not applicable as we show in the “Results” section.

DemuxEM is similar to GMM-Demux in principle: it assumes that HTO antibodies in a GEM come from two separate sources—antibodies from the background and antibodies from sample staining. However, it differs from GMM-Demux in modeling the background antibodies. GMM-Demux models the background antibodies as free-floating antibodies that re-bind to cells in pooling. demuxEM models the background antibodies as free-floating antibodies that never bind to any cell but are encapsulated in the GEM emulsion. As a result, demuxEM derives the background antibody distribution by examining empty droplets—droplets that do not contain any cell, instead of examining the antibody distributions of the cell-enclosing droplets. Through our experiments, we observe that this core assumption of demuxEM is flawed. Most empty droplets have close-to-zero antibody counts in all samples while most cell-enclosing droplets (GEMs) have decent antibody counts in all samples. As a result, demuxEM underestimates the background antibody distribution, which reduces its classification accuracy, as our simulation shows. A more detailed analysis of background antibodies is provided in Additional file 1: Section S11.

Finally, none of the above methods proposes a model for the GEM formation process and none of them models SSMs. As a result, they are incapable of estimating the post-MSM-removal multiplet percentages and they cannot authenticate putative cell types.

Prior to sample barcoding, multiplets can be identified experimentally by mixing samples of different donors. The most reliable method of finding multiplets involves mixing cells of different species [6, 13, 18, 48]. Multiplets are identified as GEMs whose reads are confidently mapped across multiple species. However, this method does not work when mixing samples of the same species. Instead, when working with samples of the same species, as long as the donors show sufficient amount of genetic variations, then multiplets can be identified as GEMs which contain distinct genetic signatures from multiple donors [12]. Unfortunately, neither method works when samples come from a single donor, which limits their applicability in scaling up single cell experiments. Sample barcoding, on the other hand, is capable of identifying multiplets even when samples are drawn from the same donor.

Besides the aforementioned methods, it is also plausible to identify some doublets through examining single cell expression profiles. When working with assays that contain multiple cell types, under the assumption that cells of the same type have highly similar expression profiles while cells of different cell types have drastically different expression profiles, multiplets are identified as small GEM groups whose expression profiles share similarities to multiple distinct large GEM groups or to multiple expression profiles of known distinct cell types [35, 48]. This idea can be further expanded to artificially create synthetic doublets from a single cell dataset and detect doublets by selecting GEMs whose expression profiles resemble synthetic doublets [22, 44]. While the idea has shown promise, a major limitation of RNA-based doublet finding studies is the lack of reliable evaluation mechanisms. The most reliable evaluation methods that are employed in these studies are still cross-species validation, cross-donor validation, and cross-cell-type validation. In cross-cell-type validations, cell types of distant expression profiles are employed to secure reliable identifications of phony cell types. GMM-Demux supplements RNA-based doublet finding studies by providing an additional means for evaluating the efficacy of their doublet identification results.

Sample barcoding provides an additional domain to the above experimental methods and has a wider applicability. Cross-species, cross-donor, and cross-cell-type multiplet identification methods rely on biological features of their respective domains, while sample barcoding gives the end users the freedom to customize the experiment, fine-tune the multiplet detection resolution, and bypass the reliance on biological features. For instance, in our Memory T dataset, cells of all five samples come from the same donor and consist of a single cell type. None of the traditional multiplet identification methods is applicable to this experiment, as there is only a single species, a single donor and a single cell type. Sample barcoding-based multiplet detection methods, such as GMM-Demux, demuxEM, and MULTI-seq, remain functional as they do not rely on a specific set of biological features. GMM-Demux, specifically, is able to work in junction with multiplet identification methods of other domains (when possible). It can use the sample barcoding information to authenticate multiplet classifications predicted by methods of other domains (when applicable).

There are only a few prior studies on modeling multiplet rates. Demuxlet [12], a genetic variation-based multiplet identifier, models the singlet rate as $(1-d_{0})^{\frac {Y}{Y_{0}}}$, where *Y* is the planned number of cells and *d*_0_ is the observed doublet rate (obtained through a mixed-species experiment) when loading *Y*_0_ cells in library preparation. By default, Demuxlet assumes *d*_0_=0.01 with *Y*_0_=1*K*. Although not elaborated in the Demuxlet paper, we notice that the singlet rate equation in Demuxlet bears a striking resemblance to the singlet rate equation used by GMM-Demux. Specifically, within the range of *Y*∈[1*K*,40*K*], ${(1-\frac {1}{100})^{\frac {Y}{1,000}} \simeq (1-\frac {1}{(100,000)})^{Y}}$. This is because the curve $f(x) = (1 - \frac {x}{100,000})^{\frac {Y}{x}}$ is almost flat within *x*∈[1,10,000]. Hence, the singlet formula used by Demuxlet under *d*_0_=0.01 and *Y*_0_=1*K* can be approximately explained by GMM-Demux as randomly partitioning *Y* cells among a total of *X*=100*K* cell-assay droplets. Despite apparent similarities between their formulas, GMM-Demux and Demuxlet employ different underlying statistical mechanics. Demuxlet uses a discriminative model, which uses regression to subjectively model the multiplet rate as a parametrized curve. GMM-Demux, on the other hand, uses a self-explanatory, generative model that directly simulates the GEM formation process. The generative model allows GMM-Demux to estimate the MSM rates of pure-type and phony-type GEM clusters in a sample barcoding dataset, while the discriminative model of Demuxlet does not. The generative model also enables GMM-Demux to accurately simulate multiplets, including both pure-type and phony-type GEMs; singlets; SSMs; and MSMs, whereas Demuxlet cannot.

Alternatively, other works model the number of cells in a GEM with Poisson distributions [3, 7, 25]. A major downside of this branch of methods is the difficulty in estimating the model parameters. A Poisson model uses the average number of cells in a GEM as its parameter. However, this number changes when the number of loaded cells changes. As a result, these models cannot be readily used for experiment planning. Interestingly, Poisson distribution is a special case of the binomial distribution, where the number of probabilistic experiments in the binomial process (*X*, in this case) approaches infinity [28]. Poisson distribution is often used as a numerical approximation of binomial distributions, especially when the number of droplets (X) is large and the average number of cells in a droplet is small. Poisson distribution-based multiplet rate estimators in fact support the GEM formation model of GMM-Demux and can be considered as numerical approximations of GMM-Demux.

Despite outperforming existing methods, the underlying assumptions of GMM-Demux impose a number of limitations. First, GMM-Demux assumes a wide gap in the HTO concentrations before and after sample pooling. HTO concentration gaps are key to defining the two peaks in the bimodal distribution of HTO UMI counts. Although from our observation, the two peaks are always well defined and are always far apart from each other in the HTO UMI count distributions, this is not 100% guaranteed, especially when the sample number is low (e.g., *M*=2). When pooling fewer samples together, the HTO concentration reduction by pooling could diminish. However, this is more of a limitation of the sample barcoding technology, rather than a limitation of GMM-Demux. Based on the premise of the sample barcoding technology, which strives to tag only sample-specific cells with HTOs, we believe that the bimodal distribution assumption should always hold. Second, the online experiment planner requires prior knowledge of the number of cell-assay droplets generated by the library preparation equipment. We suggest users profile their library preparation equipment once with GMM-Demux for the cell-assay droplet count and use the profiled number in future experiment planning. While it is logical to assume that the same library preparation equipment generates the same number of cell-assay droplets over repetitive runs, this is yet to be confirmed. In reality, based on the total volume of the loaded cell assay, the total count of cell-assay droplets could vary, even if the cell-assay pump operates at a constant frequency. Such variation, however, does not affect the MSM classifier, the SSM rate estimator, or the putative cell-type authenticator. It only affects the online experiment planner and can be potentially alleviated by running the experiment planner with a suite of likely cell-assay droplet configurations. Third, GMM-Demux cannot identify phony-type GEMs on its own. Rather, GMM-Demux authenticates pre-clustered, potential cell type-defining GEM groups. The efficacy of the cell-type authentication result depends on the quality of the clustering input: GMM-Demux is able to accurately classify GEM groups into pure-type and phony-type GEM clusters if the clustering input has high fidelity (GEMs of different cell types are organized into individual clusters). Otherwise, given a low-quality clustering input, GMM-Demux will label most clusters as mixture GEM clusters. By decoupling clustering from cell-type authentication, GMM-Demux provides the end users the freedom of selecting and customizing the clustering algorithm that best fits their specific applications. Finally, GMM-Demux assumes cells are partitioned into droplets independently. This model does not consider the volume taken up by each cell. A more realistic model would assign diminishing likelihoods to having additional cells partitioned into a droplet as more cells accumulate in the droplet. To that end, GMM-Demux does not take cell size differences into consideration either. As cells differ in size, a more accurate model would assign a smaller likelihood to having two large cells partitioned into the same droplet than that of two small cells. Unfortunately, the cell size and droplet size information is not readily available in sample barcoding data, which limits us from studying the effect of cell size on multiplet rates. Nevertheless, given that the probability of a droplet containing more than three cells is already close to zero according to our current droplet formation model, and the fact that the cell-assay droplet size has to be large enough to accommodate the largest possible cell in a tissue, we believe it is unnecessary to further complicate the GEM formation model to include the cell size information.

We further benchmarked GMM-Demux with an additional 4-HTO colonoscopic biopsy cell-hashing experiment from paired inflamed and uninflamed biopsies from a patient with Crohn’s disease. We observed results that are in concordance with the PBMC and the Memory T datasets in the “Results” section. The medical use-only colonoscopic biopsy dataset is excluded from the main results because of privacy constraints.

## Conclusion

In this paper, we proposed a model-based Bayesian framework, GMM-Demux, for detecting sample barcoding-detectable multiplets in a sample barcoding dataset, estimating the percentage of sample barcoding-undetectable multiplets in the remaining dataset, predicting the multiplet rates of planned future sample barcoding experiments, and validating the existence of putative cell types. At its core, GMM-Demux uses Gaussian mixture models to identify GEMs that contain sample-specific cells and then uncovers MSMs by selecting GEMs that contain cells from multiple samples. We showed that GMM-Demux accurately and consistently classifies GEMs into SSDs and MSMs and generates more accurate and more consistent results when compared against existing methods. We further proposed a GEM formation model to estimate the SSM rate in a sample barcoding dataset. The GEM formation model describes the GEM formation process as an augmented binomial process. We showed that the GEM formation model accurately characterizes the GEM formation process. We built an online experiment planner that estimates the multiplet rate of planned future sample barcoding (or an ordinary single cell) experiments. Then, we used the online experiment planner to generate a series of multiplet profiles under various experimental setups. Finally, we proposed putative cell type authenticator that authenticates the existence of putative cell type-defining GEM clusters, and showed that GMM-Demux correctly identifies phony-type GEM clusters in single cell datasets.

GMM-Demux is the first work that is able to not only accurately and consistently classify sample barcoding-detectable MSMs in a sample barcoding dataset, but also estimate the undetectable SSM rates among the remaining SSDs. Furthermore, GMM-Demux is the first work attempting to model the GEM formation process using a generative model. GMM-Demux incorporates its GEM formation model into an online experiment planner that is capable of anticipating experimental outcomes of planned sample barcoding experiments, and it is a first in systematically verifying the legitimacy of putative cell types using sample barcoding information.

In our future work, we intend to perform more sample barcoding experiments with different tissues and investigate the underlying mechanisms that govern the number of cell-assay droplets and the capture rate in a sample barcoding experiment. We seek to expand the GEM formation model and use it to detect false lineage discoveries and false cell-type discoveries in single cell data analysis. We also plan to investigate how to identify SSMs within SSDs.

## Methods

GMM-Demux is built around four goals: (1) separate MSMs from SSDs in a sample barcoding dataset; (2) estimate singlet and SSM rates of a sample barcoding dataset; (3) plan future sample barcoding experiments—estimate the anticipated MSM, SSM, and singlet rates of a planned future experiment; and (4) determine whether a homogeneous GEM cluster is a pure-type GEM cluster. GMM-Demux has two separate components: (1) a Gaussian mixture model-based MSM classifier and (2) a model-based SSM rate estimator. The MSM classifier classifies GEMs into MSMs and SSDs using Gaussian mixture models and computes the likelihood of each classification. The SSM rate estimator estimates the SSM and the singlet rate of the dataset. The SSM rate estimator models the GEM formation process as an augmented binomial process. It infers the latent parameters of the model, such as the number of cells of each sample and the number of cell-assay droplets formed during sequencing, from observed variables, including the number of cell-enclosing GEMs of each sample and the number of MSMs of each sample pair. Finally, the SSM rate estimator computes the estimated singlet and SSM rates of each sample with the inferred latent parameters. With the GEM formation model, GMM-Demux determines whether a proposed homogeneous GEM cluster is a pure-type GEM cluster, a phony-type GEM cluster, or a mixture cluster.

Based on the GEM formation model, we build an online sample barcoding experiment planner that estimates the multiplet rates of future sample barcoding experiments. Researchers can use the experiment planner to anticipate the outcome of a sample barcoding experiment without actually conducting the experiment. The online experiment planner takes the number of cells planned for sequencing as well as the number of samples planned for sample barcoding as inputs and outputs of the estimated MSM, SSM, and singlet rates of the anticipated outcome.

### Multi-sample multiplet (MSM) classifier

The MSM classifier pre-processes the HTO matrix with centered-log-ratio (CLR) normalization [35, 36]. CLR normalizes the HTO UMI counts of each GEM column-wise (sample-wise) as follows:

1 $$ x_{i}^{l} = \text{log} \frac{\bar{x}_{i}^{l}}{(\prod_{j=1}^{n} \bar{x}_{j}^{l})^{\frac{1}{n}}}  $$

Here, $x_{i}^{l}$ denotes the CLR-normalized HTO UMI count of the *l*th sample in the *i*th GEM (the *i*th row and the *l*th column of the HTO matrix); $\bar {x}_{i}^{l}$ denotes the original HTO UMI count of the *l*th sample in the *i*th GEM and *n* denotes the total number of GEMs.

**Fig. 11:**
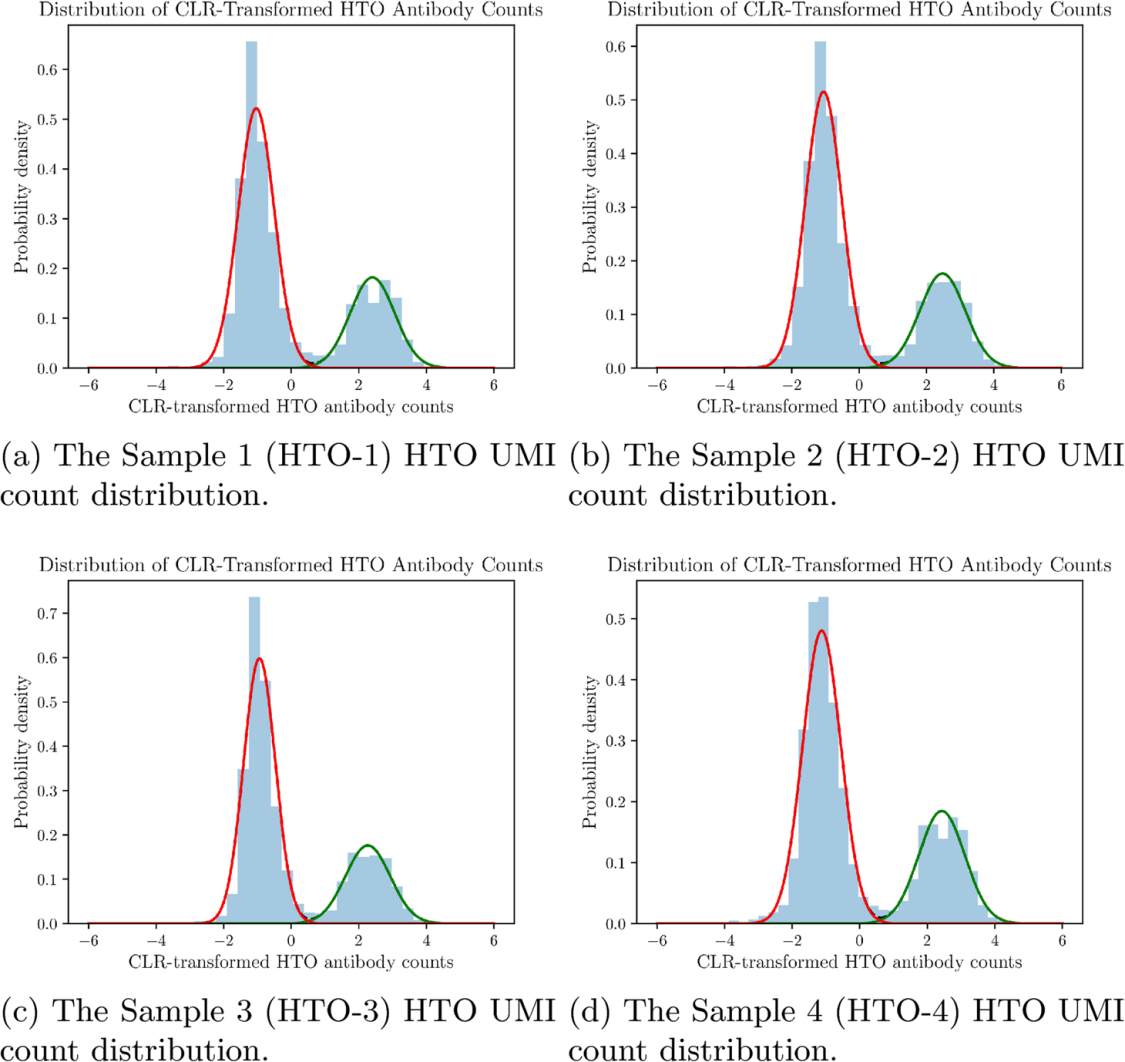
Distributions of CLR-transformed HTO UMI counts from a 4-sample cell-hashing experiment. Each distribution is decomposed into two Gaussian distributions, $\mathcal {N}_{high}(\mu _{high}^{\hat {l}},\,(\sigma _{high}^{\hat {l}})^{2})$ and $\mathcal {N}_{low}(\mu _{low}^{\hat {l}},\,(\sigma _{low}^{\hat {l}})^{2})$. $\mathcal {N}_{high}(\mu _{high}^{\hat {l}},\,(\sigma _{high}^{\hat {l}})^{2})$ represents the HTO UMI count distribution of cell-enclosing GEMs. $\mathcal {N}_{low}(\mu _{low}^{\hat {l}},\,(\sigma _{low}^{\hat {l}})^{2})$ represents the HTO UMI count distribution of cell-free GEMs

The distributions of the CLR-transformed HTO UMI counts of a 4-sample cell-hashing experiment are illustrated in Fig. 11. From this figure, we observe that for each sample, the CLR-transformed HTO UMI counts follow a bimodal distribution which resembles a mixture of two Gaussian distributions. GMM-Demux models the HTO UMI count distribution with an aggregated two-Gaussian distribution mixed model. We color the two distributions as red and green, respectively, in Fig. 11. For a specific sample $\hat {l}$ ($l=\hat {l}$), the Gaussian distribution with the smaller mean, $\mathcal {N}_{low}^{\hat {l}}(\mu _{low}^{\hat {l}},\,(\sigma _{low}^{\hat {l}})^{2})$ (in red), accounts for GEMs that do not contain cells from $\hat {l}$ ($\hat {l}$-cell-free GEMs). The other distribution, $\mathcal {N}_{high}^{\hat {l}}(\mu _{high}^{\hat {l}},\,(\sigma _{high}^{\hat {l}})^{2})$ (in green), on the contrary, models GEMs that contain cells from $\hat {l}$ ($\hat {l}$-cell-enclosing GEMs). It is worth noting that GEMs from $\mathcal {N}_{low}^{\hat {l}}(\mu _{low}^{\hat {l}},\,(\sigma _{low}^{\hat {l}})^{2})$ still have positive HTO counts. In cell hashing, when cell assays of all samples are pooled together, free-floating HTO antibodies that have not yet bound to any cell still exist in the solution, as shown in Additional file 1: Figure S2. These residual free-floating HTO antibodies bind randomly to all cells from all samples (the restaining step in Additional file 1: Figure S2). However, as cell assays are pooled together, antibodies are diluted; hence, $\mathcal {N}_{low}^{\hat {l}}(\mu _{low}^{\hat {l}},\,(\sigma _{low}^{\hat {l}})^{2})$ has a lower mean ($\mu _{low}^{\hat {l}} < \mu _{high}^{\hat {l}}$).

GEMs from $\mathcal {N}_{high}^{\hat {l}}(\mu _{high}^{\hat {l}},\,(\sigma _{high}^{\hat {l}})^{2})$, on the other hand, bind with HTO antibodies prior to pooling of samples. Before pooling, HTO antibodies have much higher concentrations. As a result, $\mathcal {N}_{high}^{\hat {l}}(\mu _{high}^{\hat {l}},\,(\sigma _{high}^{\hat {l}})^{2})$ has a higher mean.

For each sample, GMM-Demux uses its Gaussian mixture model to find GEMs that contain cells from the sample. Given a GEM, *i*, and a sample $\hat {l}$, GMM-Demux tests whether $x_{i}^{\hat {l}}$ originates from the $\mathcal {N}_{high}^{\hat {l}}(\mu _{high}^{\hat {l}},\,(\sigma _{high}^{\hat {l}})^{2})$ distribution of $\hat {l}$: if $x_{i}^{\hat {l}}$ originates from $\mathcal {N}_{high}^{\hat {l}}(\mu _{high}^{\hat {l}},\,(\sigma _{high}^{\hat {l}})^{2})$, then *i* must contain cells from $\hat {l}$; otherwise, $x_{i}^{\hat {l}}$ must belong to $\mathcal {N}_{low}^{\hat {l}}(\mu _{low}^{\hat {l}},\,(\sigma _{low}^{\hat {l}})^{2})$, which means GEM *i* does not contain cells from $\hat {l}$.

Let $Z_{i}^{\hat {l}}=high$ denote the event that $x_{i}^{\hat {l}}$ originates from $\mathcal {N}_{high}^{\hat {l}}(\mu _{high}^{\hat {l}},\,(\sigma _{high}^{\hat {l}})^{2})$ and $Z_{i}^{\hat {l}}=low$ denote the event that $x_{i}^{\hat {l}}$ originates from $\mathcal {N}_{low}^{\hat {l}}(\mu _{low}^{\hat {l}},\,(\sigma _{low}^{\hat {l}})^{2})$. Let $P(Z_{i}^{\hat {l}}=high)$ and $P(Z_{i}^{\hat {l}}=low)$ denote the prior probability of GEM *i* originating from $\mathcal {N}_{high}^{\hat {l}}(\mu _{high}^{\hat {l}},\,(\sigma _{high}^{\hat {l}})^{2})$ and $\mathcal {N}_{low}^{\hat {l}}(\mu _{low}^{\hat {l}},\,(\sigma _{low}^{\hat {l}})^{2})$, respectively. Then, the probability of observing HTO count value $x_{i}^{\hat {l}}$ in GEM *i* equals to:

2 $$ P(x_{i}^{\hat{l}}) = P(x_{i}^{\hat{l}} \mid Z_{i}^{\hat{l}}=high) \cdot P(Z_{i}^{\hat{l}}=high) + P(x_{i}^{\hat{l}} \mid Z_{i}^{\hat{l}}=low) \cdot P(Z_{i}^{\hat{l}}=low)  $$

where $P(x_{i}^{\hat {l}} \mid Z_{i}^{\hat {l}}=high) \sim \mathcal {N}_{high}^{\hat {l}}(\mu _{high}^{\hat {l}},\,(\sigma _{high}^{\hat {l}})^{2})$ and $P(x_{i}^{\hat {l}} \mid Z_{i}^{\hat {l}}=low) \sim \mathcal {N}_{low}^{\hat {l}}(\mu _{low}^{\hat {l}},\,(\sigma _{low}^{\hat {l}})^{2})$.

GMM-Demux computes the mean and the standard deviation of $\mathcal {N}_{high}^{l}(\mu _{high}^{\hat {l}},\,(\sigma _{high}^{\hat {l}})^{2})$ and $\mathcal {N}_{low}^{l}(\mu _{low}^{\hat {l}},\,(\sigma _{low}^{\hat {l}})^{2})$, as well as the prior probabilities $P(Z_{i}^{l}=high)$ and $P(Z_{i}^{l}=low)$ of each sample *l* using the Expectation Maximization (EM) Technique [34].

With all Gaussian mixture models computed across all samples, for each GEM *i*, GMM-Demux computes the posterior probability of GEM *i* containing cells from sample $\hat {l}$. Let $P(Z_{i}^{\hat {l}}=high \mid x_{i}^{\hat {l}})$ denote the posterior probability of $x_{i}^{\hat {l}}$ originating from $\mathcal {N}_{high}^{\hat {l}}(\mu _{high}^{\hat {l}},\,(\sigma _{high}^{\hat {l}})^{2})$, and $P(Z_{i}^{\hat {l}}=low \mid x_{i}^{\hat {l}})$ denote the probability of $x_{i}^{\hat {l}}$ originating from $\mathcal {N}_{high}^{\hat {l}}(\mu _{high}^{\hat {l}},\,(\sigma _{high}^{\hat {l}})^{2})$. Both posterior probabilities ($P(Z_{i}^{\hat {l}}=high \mid x_{i}^{\hat {l}})$ and $P(Z_{i}^{\hat {l}}=low \mid x_{i}^{\hat {l}})$) are computed using Bayes’ rule:

3 $$ \arraycolsep=1.4pt\def\arraystretch{2.2} \begin{array}{ll} P(Z_{i}^{\hat{l}}=high \mid x_{i}^{\hat{l}}) = \frac{P(x_{i}^{\hat{l}} \mid Z_{i}^{\hat{l}}=high) \cdot P(Z_{i}^{\hat{l}}=high)} {P(x_{i}^{\hat{l}})} \\ P(Z_{i}^{\hat{l}}=low \mid x_{i}^{\hat{l}}) = \frac{P(x_{i}^{\hat{l}} \mid Z_{i}^{\hat{l}}=low) \cdot P(Z_{i}^{\hat{l}}=low)} {P(x_{i}^{\hat{l}})} \end{array}  $$

The probability ($P(i \in SSD_{\hat {l}})$) of *i* being a single-sample droplet (SSD) of sample $\hat {l}$ ($SSD_{\hat {l}}$) can be computed as:

4 $$ P(i \in SSD_{\hat{l}}) = P(Z_{i}^{\hat{l}}=high \mid x_{i}^{\hat{l}}) \cdot \prod_{l \neq \hat{l}} P(Z_{i}^{l}=low \mid x_{i}^{l})  $$

The probability of *i* being a multi-sample multiplet (MSM) can be computed as:

5 $$ P(i \in MSM) = 1 - \sum_{l} P(i \in SSD_{l})  $$

GMM-Demux classifies GEMs by ranking above probabilities: a GEM *i* is classified as a SSD of $\hat {l}$ if $P(i = SSD_{\hat {l}})$ is the largest among all, or as a MSM if *P*(*i*=*MSM*) is the largest among all.

In fact, GMM-Demux is able to compute the probability of a GEM containing cells of any specific multi-sample configuration. Assume *U* is a set of samples (e.g., sample *l*_1_ and sample *l*_4_). The probability of GEM *i* containing cells from *U*, *MSM*_*U*_, can be computed by:

6 $$ P(i \in MSM_{U}) = \prod_{l \in U} P(Z_{i}^{l}=high \mid x_{i}^{l}) \cdot \prod_{l \notin U} P(Z_{i}^{l}=low \mid x_{i}^{l})  $$

This allows GMM-Demux to not only identify and count SSDs, but also identify and count MSMs of specific sample combinations in a sample barcoding dataset. Counting MSMs of specific sample combinations is key to verifying the correctness of the SSM rate estimator, as we will show in later sections.

GMM-Demux lets the user specify a confidence cutoff *c* to filter out uncertain classifications. Sometimes, GEMs have HTO UMI counts that reside in the junction area between $\mathcal {N}_{high}^{l}(\mu _{high}^{\hat {l}},\,(\sigma _{high}^{\hat {l}})^{2})$ and $\mathcal {N}_{low}^{l}(\mu _{low}^{\hat {l}},\,(\sigma _{low}^{\hat {l}})^{2})$ on a HTO sample dimension. Such GEMs produce ambiguous classification results: they have similar likelihoods between multiple classifications, which typically are all below 0.5. Uncertain GEMs are pruned by the confidence cutoff *c*: GEMs with maximum probabilities across all classifications which are less than *c* are deemed *uncertain GEMs* and are removed from the population. By tweaking *c*, GMM-Demux allows users to adjust the level of rigorousness in identifying SSDs and MSMs.

### Same-sample multiplet (SSM) rate estimator

As previously discussed, sample barcoding cannot distinguish SSMs from singlets. While GMM-Demux does not seek to identify SSMs in SSDs, it estimates the percentage of SSMs and singlets in each sample using the SSM rate estimator. Estimating the SSM rate in a dataset is critical for quality control. SSM rate represents the noise level of a sample. Samples with high SSM rates have low quality and should be removed.

GMM-Demux estimates the percentage of SSMs among all GEMs using a probabilistic model that models the entire GEM formation process in sample barcoding. The GEM formation process occurs after pooling of samples and governs the subsequent random distribution of cells into GEMs. GMM-Demux models the GEM formation process as an augmented binomial process: it assumes that after pooling of samples, the entire cell assay is divided into a finite number of droplets, called cell-assay droplets. Each cell is randomly and independently partitioned into a cell-assay droplet. During the single cell barcoding process, a fraction of all cell-assay droplets are combined with gel beads and form GEMs. The rest of the cell-assay droplets do not form GEMs and will not be sequenced. We use the term *droplet capture rate* to denote the probability that a cell-assay drop is combined with a gel bead. GEMs, which contain both cell-enclosing cell-assay droplets and gel beads, are recovered after sequencing and are summarized in a HTO matrix. A detailed illustration of the GEM formation model is provided in Additional file 1: Section S1.

The rates of multiplets, including both SSM rates and MSM rates, are modeled as the probability of having multiple cells (from the same or different samples) partitioned into the same cell-assay droplet. A major challenge for this method is that key parameters, namely the number of cells in each sample, the droplet capture rate, and the total number of cell-assay droplets, are not directly observable. Instead, from the MSM classifier, we observe the number of sample-specific GEMs as well as the number of MSMs of any sample pair. Combined with the prior knowledge of the estimated total number of cells loaded for sample barcoding, the SSM rate estimator derives the latent parameters of the model and uses the complete model to estimate the multiplet rates of the dataset.

#### Modeling multiplets

The SSM rate estimator models the GEM formation process as follows: Assume there are a total of *X* cell-assay droplets. Also assume there are *y*_*l*_ cells in a sample, *l*, with *Y* denoting the overall population of all cells, or $Y = \sum _{l} y_{l}$. The model assumes that each cell is independently and randomly partitioned into a cell-assay droplet. Consequently, a cell has a probability of 1/*X* to reside within a specific cell-assay droplet. Assuming that no bias exists among cells from different samples, then the probability of a cell-assay droplet, *i*, being a singlet, given that *i* is not empty, can be calculated as:

7 $$ P(i \in \text{\texttt{singlet}} \mid i \in \mathtt{non{\text -}empty}) \approx \frac{\mathbb{E}[\#_{\text{\texttt{singlets}}}]}{\mathbb{E}[\#_{\mathtt{non{\text -}empty\ drops}}]}  $$

where $\mathbb {E}[\#_{\text {\texttt {singlets}}}]$ is the expected number of singlets and $\mathbb {E}[\#_{\mathtt {non{\text -}empty drops}}]$ is the expected number of non-empty cell-assay droplets. For simplicity, in the rest of this paper, we refer to cell-assay droplets simply as droplets.

Since cells are randomly partitioned into droplets, $\mathbb {E}[\#_{\text {\texttt {singlets}}}]$ can be computed from a binomial model. Specifically, we have ${\mathbb {E}[\#_{\text {\texttt {singlets}}}] = X \cdot P(i \in {\text {\texttt {singlet}}})}$, where *P*(*i*∈singlet) denotes the probability of having one and only one cell, out of a total of *Y* cells, residing in *i*. All other cells are partitioned into other droplets. Mathematically, we have:

8 $$ \mathbb{E}[\#_{\text{\texttt{singlets}}}] = X \cdot \binom{Y}{1} \frac{1}{X} (1 - \frac{1}{X})^{Y-1}  $$

Similarly, the expected number of non-empty droplets can be computed as $\mathbb {E}[\#_{\mathtt {non{\text -}empty\ drops}}] = X \cdot P(i \in {\mathtt {non{\text -}empty\ drops}})$. *P*(*i*∈non-emptydrops) is the probability of *i* being non-empty, and it equals to 1−*P*(*i*∈emptydrops). According to binomial distribution, *P*(*i*∈emptydrops) equals to the probability of all cells residing in droplets other than *i*. Overall, we have:

9 $$ \mathbb{E}[\#_{\mathtt{non{\text -}empty\ drops}}] = X \cdot (1 - (1 - \frac{1}{X})^{Y})  $$

Equally, the probability of *i* being a MSM given *i* is not empty, *P*(*i*∈MSM), can be computed as:

10 $$ P(i \in \text{\texttt{MSM}} \mid i \in \mathtt{non{\text -}empty}) = 1 - \frac{P(i \in \text{\texttt{SSD}})}{P(i \in \mathtt{non{\text -}empty})}  $$

with *P*(*i*∈SSD) denoting the probability of *i* being a SSD.

When more than one sample is labeled in sample barcoding, we have $P(i \in {\text {\texttt {SSD}}})= \sum _{l}P(i \in \text {\texttt {SSD}}_{l})$, where *P*(*i*∈SSD_*l*_) is the probability of *i* being a SSD of sample *l*. Let set *D*_*l*_ represent all and only *l*-cell-enclosing droplets and set $D_{l}^{C}$ to represent all and only *l*-cell-free droplets. The probability of *i* being a SSD of sample $\hat {l}$ ($\text {\texttt {SSD}}_{\hat {l}}$) equals the probability of *i* being a cell-enclosing droplet in $\hat {l}$ and a cell-free droplet in all other samples. Based on binomial distribution, the probability of *i* belonging to $D_{\hat {l}}^{C}$, $P(i \in D_{\hat {l}}^{C})$, equals the probability of all cells of $\hat {l}$ residing in droplets other than *i*, which is $(1 - \frac {1}{X})^{y_{\hat {l}}}$. As $D_{\hat {l}}$ and $D_{\hat {l}}^{C}$ complement each other, we have $P(i \in D_{\hat {l}}) = 1 - P(i \in D_{\hat {l}}^{C})$. We expand $P(i \in \text {\texttt {SSD}}_{\hat {l}})$ into the following:

11 $$ P(i \in \text{\texttt{SSD}}_{\hat{l}}) = P(i \in {D_{\hat{l}}}) \cdot \prod_{l\neq\hat{l}}P(i \in D_{l}^{C})  $$

where $P(i \in D_{\hat {l}})$ and $\prod _{l\neq \hat {l}}P(i \in D_{l}^{C})$ can be computed as:

12 $$ \arraycolsep=1.4pt\def\arraystretch{2.2} \begin{array}{ll} P(i \in D_{\hat{l}}) = 1 - P(i \in D_{\hat{l}}^{C}) = 1 - (1 - \frac{1}{X})^{y_{\hat{l}}} \\ \prod_{l\neq\hat{l}}P(i \in D_{l}^{C}) = (1 - \frac{1}{X})^{\sum_{l\neq\hat{l}}y_{l}} \end{array}  $$

Finally, the probability of *i* being a SSM is simply the probability of *i* being neither a MSM nor a singlet. Mathematically, we have:

13 $$ P(i \in \text{\texttt{SSM}}) = 1 - P(i \in \text{\texttt{singlet}}) - P(i \in \text{\texttt{MSM}})  $$

Alternatively, we can compute *P*(*i*∈SSM) as $P(i \in \text {\texttt {SSM}}) = \sum _{l} P(i \in \text {\texttt {SSM}}_{l})$, with *P*(*i*∈SSM_*l*_) denoting the probability of *i* being a SSM of sample *l*. Because a SSD of *l* must be either a SSM of *l* or a singlet of *l*, therefore, event {*i*∈SSM_*l*_∣*i*∈SSD_*l*_} and event {*i*∈singlet_*l*_∣*i*∈SSD_*l*_)} must be collectively exhaustive events. Together, *P*(*i*∈SSM_*l*_) can be computed as:

14 $$ P(i \in \text{\texttt{SSM}}_{l}) = P(i \in \text{\texttt{SSD}}_{l})\cdot (1 - P(i \in \text{\texttt{singlet}}_{l} \mid i \in \text{\texttt{SSD}}_{l}))  $$

Since all singlets of *l* are SSDs of *l*, we have:

15 $$ \arraycolsep=1.4pt\def\arraystretch{2.2} \begin{array}{ll} P(i \in \text{\texttt{singlet}}_{l} \mid i \in \text{\texttt{SSD}}_{l}) \approx \frac{\mathbb{E}[\#_{\text{\texttt{singlets}}_{l}}]}{\mathbb{E}[\#_{\text{\texttt{SSD}}_{l}}]} \\ \mathbb{E}[\#_{\text{\texttt{singlets}}_{l}}] = X \binom{y_{l}}{1} \frac{1}{X} (1 - \frac{1}{X})^{Y-1} \end{array}  $$

The two methods (Eqs. (13) and (14)) of calculating *P*(*i*∈SSM) are equivalent (details are omitted to conserve space).

Overall, given *X* and *y*_*l*_ for every sample *l* of a sample barcoding dataset, the SSM rate estimator estimates the singlet rate (*P*(*i*∈singlet), Eq. (7)), the MSM rate (*P*(*i*∈MSM), Eq. (10)), and the SSM rate (*P*(*i*∈SSM), Eqs. (13) and (14)) of the dataset. Unlike the SSM rate, which can only be inferred indirectly through the GEM formation model, the MSM rate can be obtained both analytically through the GEM formation model and numerically by interpreting the MSM classification result. As a result, we can validate the correctness of the GEM formation model by comparing the MSM rates obtained through both methods. In the “Results” section, we show that both methods provide consistent MSM rates.

We perform simulations to verify the correctness of the above equations. The simulation results are included in Additional file 1: Section S2. Specifically, we repeatedly simulate the GEM formation process. We show that the singlet, SSM, and MSM rates measured from simulations asymptotically match the values analytically computed with above equations.

#### Estimating model parameters

GMM-Demux relies on *X* and *y*_*l*_ of every sample *l* to compute the SSM rates. However, neither *X* nor *y*_*l*_ is directly observable in a sample barcoding dataset. Instead, from the classification result, GMM-Demux observes *z*_*l*_, the number of GEMs in *D*_*l*_.

Let *r*_*cap*_ denote the droplet capture rate. From *z*_*l*_ and a user-provided estimation of the total cell count, *Y*, GMM-Demux computes both *X*, *r*_*cap*_, and *y*_*l*_. For a HTO sample *l*, based on our multiplet model, we have $P(i \in D_{l}^{C} \mid X, y_{l}) = (1 - \frac {1}{X})^{y_{l}}$ (*X* and *y*_*l*_ serve as parameters) and $P(i \in D_{l} \mid X, y_{l}) = 1 - P(i \in D_{l}^{C} \mid X, y_{l})$. Let random variable *Z*_*l*_ denote the number of GEMs that enclose cells from *l* and let *P*(*Z*_*l*_=*z*_*l*_∣*X*,*r*_*cap*_,*y*_*l*_) denote the probability of observing *z*_*l*_*l*-cell-enclosing GEMs under the parameter set [*X*,*r*_*cap*_,*y*_*l*_]. According to the GEM formation model, which models partitioning of cells into droplets with a binomial distribution, we have:

16 $$ {\begin{aligned} \arraycolsep=1.4pt\def\arraystretch{2.2} \begin{array}{ll} P(Z_{l}=z_{l} \mid X,r_{cap},y_{l}) = \binom{X}{\frac{z_{l}}{r_{cap}}} (P(i \in D_{l} \mid X,y_{l}))^{\frac{z_{l}}{r_{cap}}} (P(i \in D_{l}^{C} \mid X,y_{l}))^{X - \frac{z_{l}}{r_{cap}}} \\ P(Z_{1} = z_{1}, Z_{2} = z_{2}, \dots, Z_{M} = z_{M} \mid X,r_{cap},y_{1},y_{2}, \dots,y_{M}) = \prod_{l = 1}^{M} P(Z_{l}=z_{l} \mid X,r_{cap},y_{l}) \end{array} \end{aligned}}  $$

We derive the model parameters by computing:

17 $$ \arraycolsep=1.8pt\def\arraystretch{2.2} \begin{array}{lllll} \begin{aligned} & \underset{X,r_{cap},y_{1},\dots,y_{M}}{arg\,max} & & P(Z_{1} = z_{1}, Z_{2} = z_{2}, \dots, Z_{M} = z_{M} \mid X,r_{cap},y_{1},y_{2}, \dots,y_{M}), \\ & \text{subject to} & & X > 0 \\ & & & \sum_{l=1}^{M} y_{l} = Y \\ & & & y_{l} > 0, \; l = 1, \ldots, M \\ & & & r_{cap} \in [0,1], \end{aligned} \end{array}  $$

where *Y* is the user-provided total number of cells loaded for library preparation, which can be obtained from the hemocytometer.

### Online sample multiplexing experiment planner

The online sample barcoding experiment planner estimates the singlet, SSM, and MSM rates of a planned sample barcoding experiment via the GEM formation model. Specifically, it takes the estimated number of cells (*Y*), the planned number of samples for sample barcoding (*M*), the estimated number of droplets (*X*), and the droplet capture rate (*r*_*cap*_) in library preparation as inputs, and it computes the estimated multiplet rates. The online experiment planner assumes cells are evenly distributed among *M* samples.

The online experiment planner also estimates the relative single-sample multiplet (RSSM) rate, defined as the estimated number of SSMs among SSDs. Mathematically, the RSSM rate is defined as:

18 $$ P(i \in \text{\texttt{SSM}} | i \in \text{\texttt{SSD}}) \approx \frac{\mathbb{E}[\#_{\text{\texttt{SSM}}}]}{\mathbb{E}[\#_{\text{\texttt{SSD}}}]} = \frac{\mathbb{E}[\#_{\text{\texttt{SSM}}}]}{\mathbb{E}[\#_{\text{\texttt{singlet}}}] + \mathbb{E}[\#_{\text{\texttt{SSM}}}]}  $$

The RSSM rate marks the overall quality of a sample barcoding dataset. It represents the percentage of irremovable multiplets among SSDs, after removing all MSMs in the dataset. If the RSSM rate of the estimated outcome is too high, then the planned experiment should be aborted, as the anticipated outcome will be too noisy for downstream analysis. While dividing the cell assay into more samples drives down the RSSM rate, as it reduces $\mathbb {E}[\#_{\text {\texttt {SSM}}}]$, it increases both the cost and the complexity of the experiment. With the multiplet rate estimator, researchers can determine the minimum number of HTO samples to use in a sample barcoding experiment, to save cost while meeting the RSSM rate target.

The online experiment planner computes the multiplet rates as follows:

19 $$ \arraycolsep=1.4pt\def\arraystretch{2.2} \begin{array}{ll} P(i \in \text{\texttt{singlet}}) = \frac{Y(1-\frac{1}{X})^{Y-1}}{X(1-(1-\frac{1}{X})^{Y})} \\ P(i \in \text{\texttt{MSM}}) = \frac{M(1 - (1 - \frac{1}{X})^{\frac{Y}{M}})(1-\frac{1}{X})^{\frac{Y(M-1)}{M}}}{X(1-(1-\frac{1}{X})^{Y})} \\ P(i \in \text{\texttt{SSM}}) = 1 - P(i \in \text{\texttt{singlet}}) - P(i \in \text{\texttt{MSM}}) \\ P(i \in \text{\texttt{RSSM}}) = \frac{P(i \in \text{\texttt{SSM}})}{P(i \in \text{\texttt{SSD}})} \end{array}  $$

The above equations show that the number of samples, *M*, does not affect the singlet rate. The singlet rate is solely determined by *X* and *Y*. However, a greater *M* reduces the SSM rate and increases the MSM rate. Therefore, we conclude that dividing a cell assay into more samples by sample barcoding transforms more SSMs into MSMs. Transforming SSMs into MSMs improves the quality of the dataset. With fewer SSMs, the RSSM rate of the dataset decreases. In comparison, having more MSMs does not affect the quality of the dataset, as MSMs are removed by GMM-Demux.

Given *r*_*cap*_, the online experiment planner also computes the estimated number of cell-enclosing GEMs in the final output, as well as the estimated number of SSDs after removing MSMs. The number of cell-enclosing GEMs, *#*_non-emptyGEM_=*#*_non-emptydrops_·*r*_*cap*_ (*#*_non-emptydrops_ is computed in Eq. (9)). The number of SSDs is computed as *#*_SSD_=*#*_non-emptyGEM_·(1−*P*(*i*∈MSM)).

Among all four inputs, *Y* and *M* are user-controlled while *X* and *r*_*cap*_ are largely dictated by the library preparation equipment. However, based on our observations, we found that *X* mostly varies between 65*K* and 80*K*. To account for the wide ranges of variability of the inputs, the online experiment planner uses sliders for selecting *X*, *Y*, *M*, and *r*_*cap*_, which have ranges of 60*K*– 100*K*, 1*K*– 80*K*, 1–20, and 0–1, respectively. The online experiment planner supports dynamic updates. It computes the estimated multiplet rates in real time as the user updates input parameters. In practice, we recommend that users profile their library preparation equipment once for the total number of droplets (*X*) in a sequencing run, by performing a small-scale sample barcoding experiment, and use the profiled *X* (included in the GMM-Demux output) in planning future experiments.

### Pure-type GEM verification

In novel cell-type identification, a cell-type classifier is used to group GEMs into clusters. Each cluster is assumed to represent a unique cell type. Clusters with average expression profiles that do not match any known cell types are identified as novel cell types [40].

After clustering, phony-type GEMs are grouped into distinct clusters. Phony-type GEM clusters may be incorrectly identified as novel cell types, as their expression profiles do not match known cell types, generating false discoveries. GMM-Demux rectifies true novel cell types by validating if the alleged novel cell-type GEM cluster contains mainly pure-type GEMs. Based on the GEM composition in the cluster, GMM-Demux classifies GEM clusters into three categories: pure-type GEM clusters, phony-type GEM clusters, and mixture clusters. Phony-type GEM clusters contain mostly phony-type GEMs. Pure-type GEM clusters contain mostly pure-type GEMs. Mixture clusters contain large quantities of both pure-type and phony-type GEMs.

Let *G* represent a GEM cluster. GMM-Demux classifies *G* by examining the MSM ratio of *G*. For simplicity, we assume cells are equally randomly divided into the *M* sample barcoding samples. If *G* is a phony-type GEM cluster, the MSM ratio of *G* must be very high. Elaborated in Additional file 1: Section S3, the expected MSM ratio of a phony-type cluster approaches and exceeds $1-\frac {1}{M}$. Otherwise, if *G* is a pure-type GEM cluster, its MSM ratio should not be greater than the MSM ratio of the entire sample barcoding dataset, which is much smaller than $1-\frac {1}{M}$. The MSM ratio reflects the GEM composition of *G*: in a phony-type GEM cluster, all GEMs are multiplets; hence, the MSM ratio of *G*, $r_{\mathtt {MSM_{G}}}$, equals to $r_{\mathtt {MSM_{G}}} = \frac {\#_{\mathtt {MSM_{G}}}}{\#_{\mathtt {SSM_{G}}}+\#_{\mathtt {MSM_{G}}}}$, where $\#_{\mathtt {MSM_{G}}}$ and $\#_{\mathtt {SSM_{G}}}$ denote the number of MSMs and SSMs in *G*, respectively; in a pure-type GEM cluster, however, we have $r_{\mathtt {MSM_{G}}} = \frac {\#_{\mathtt {MSM_{G}}}}{\#_{\mathtt {singlet_{G}}}+\#_{\mathtt {SSM_{G}}}+\#_{\mathtt {MSM_{G}}}}$ instead, where $\#_{\mathtt {singlet_{G}}}$ denotes the number of singlets in *G*. By comparing the two ratios, we observe that pure-type GEM clusters include singlet counts in the denominator, whereas phony-type GEM clusters do not. As a result, the MSM ratio is much higher in phony-type GEM clusters than in pure-type GEM clusters. Complex situations where cells are not evenly distributed among sample barcoding samples are discussed in Additional file 1: Section S3.

GMM-Demux uses hypothesis testing to measure the confidence of each classification. GMM-Demux prepares two hypotheses, the *phony-type hypothesis* and the *pure-type hypothesis*, which assume *G* being a pure-type or a phony-type GEM cluster, respectively. GMM-Demux tests both hypotheses with the binomial test and computes a *p* value for each hypothesis. Based on the hypothesis testing results, GMM-Demux classifies *G* as a pure-type GEM cluster, a phony-type GEM cluster, or a mixture cluster. Details of the hypothesis tests are provided in Additional file 1: Section S3.

Based on the classification result of *G*, GMM-Demux recommends different actions. Being classified as a phony-type GEM cluster suggests that the proportion of pure-type GEMs in *G*, if there exists any, is extremely small and most GEMs in *G* are phony-type GEMs. GMM-Demux recommends excluding *G* from further analysis. Being classified as a mixture cluster suggests that *G* mixes pure-type GEMs and phony-type GEMs together and has non-trivial numbers of GEMs in both categories. This is often a result of poor clustering quality where *G* becomes a super-cluster over several pure-type and phony-type GEM clusters. GMM-Demux recommends refinement over the clustering method and subdividing *G* into pure-type GEM and phony-type GEM sub-clusters. Finally, being classified as a pure-type GEM cluster suggests that it is plausible that *G* defines a real cell type. Further analysis over *G* is recommended.

### Compatibility

The GMM-Demux classifier is compatible with CellRanger-3.1.0 from 10X Genomics. It takes the sample barcoding data of post-filtering, non-empty droplets, in the market matrix (ṁtx) format, together with the estimated number of cells (*Y*), as inputs, and it outputs a double column table as the classification result. The row indices of the output table are GEM barcodes. The two columns are the classification of each GEM and the confidence score of each classification, respectively. With *M* samples, GMM-Demux classifies GEMs into a maximum of 2^*M*^+1 classes. Besides the uncertain class, the negative class, and *M* SSD classes, there are $\binom {M}{2}$ bi-sample classes, $\binom {M}{3}$ tri-sample classes, …and $\binom {M}{M}=1$*M*-sample class. Additionally, GMM-Demux produces a SSM rate summary file, which includes the SSM rate and the RSSM rate of each sample, and a summary file that includes the multiplet rates of the entire dataset. The summary file also includes the estimated number of cell-assay droplets (*X*) and the estimated droplet capture rate (*r*_*cap*_) of the library preparation equipment. Example outputs are provided in Additional file 1: Section S4.

## Supplementary information

**Additional file 1** Supplementary figures

**Additional file 2** Supplementary tables

**Additional file 3** Review history

## Data Availability

The source code of GMM-Demux is accessible at Github [45] under the MIT license and Zenodo [46]. The in-house cell-hashing datasets are available in the Gene Expression Omnibus repository, under accession GSE152981 [47]. The public PBMC cell-hashing dataset is also available in the Gene Expression Omnibus repository, under accession GSE131756 [37]. The colonoscopic biopsy dataset can be shared via a Material Transfer Agreement after it is reviewed by the University of Pittsburgh.
