# Supplementary material for: GMM-Demux: sample demultiplexing, multiplet detection, experiment planning, and novel cell-type verification in single cell sequencing
Source: Genome Biol. 2020 Jul 30;21:188. doi: 10.1186/s13059-020-02084-2 (PMC7393741; doi:10.1186/s13059-020-02084-2)
Supplement: Supplementary file 1 — Additional file 1 Supplementary figures [file 13059_2020_2084_MOESM1_ESM.pdf]

Supplementary Methods and Results

1 Modeling the General Droplet Formation Process

Outlined in the original droplet-seq paper[18], in droplet-based single cell library preparation, GEMs are formed by "co-flowing" two aqueous solutions across an oil channel to form nanoliter-sized droplets. One flow contains gel beads, microcapsules that contain barcoded microparticles suspended in a lysis buffer. The other flow contains the cell assay, which is a HTO-tagged cell suspension. Figure S1 illustrates the co-flow process. In our model, the two flows are driven by two separate pumps that operate in discrete pulses. Each pulse pumps unit volume solution into oil, forming an emulsion droplet. In our model, the two pumps does not operate in sync and their frequencies differ. Assuming the unit volume of each pulse from the cell assay pump is μ, and the total volume of the cell assay solution is V, then the total number of pulses from the cell-assay pump throughout a sample barcoding library preparation can be calculated as $X = \frac{V}{\mu}$. Therefore, in the final solution, there are a total of $X = \frac{V}{\mu}$ droplets that contain cell-assay solution.

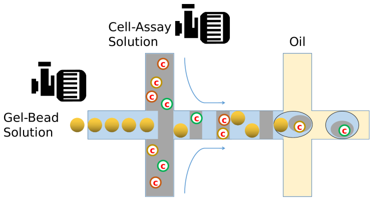

Figure S1: Illustration of the co-flow process.

Not all cell-assay droplets can be recovered through sequencing. A droplet is sequenced only if it also contains a gel bead, which is not always guaranteed. In our model, we assume r_{cap} as a constant probability of a droplet getting a gel bead. Cell-assay droplets without gel beads are not harvested through sequencing.

An illustration of the workflow of sample barcoding is provided in Figure S2

2 Validating Multiplet Rate Equations

We validate the multiplet rate equations by comparing the equation-derived multiplet rates against the multiplet rates obtained through simulation. Specifically, given $[Y, M, X]$ (Y is the total number of cells, M is the number of samples and X is the total number of cell-assay droplets), we divide Y cells evenly into M samples and randomly partition each cell into a droplet. We do not include r_{cap} in our simulation as r_{cap} does not affect the multiplet rates. We collect all droplets from the simulation; count the numbers of singlets, SSMs and MSMs and computes the singlet, SSM and MSM rates. For each $[Y, M, X]$ parameter set, we repeat the simulation 500, 1000 and 1500 times and average the singlet, SSM and MSM rates over all iterations. The comparison results are shown in Table S1, S2 and S3.

As shown in the tables, as the number of iterations increases, the multiplet rates obtained through simulation asymptotically approaches the model-derived multiplet rates. This suggests that the equations for computing multiplet rates are correct.

#Cells (Y)	#Droplets (X)	#Samples (M)	500 iterations	1000 iterations	1500 iterations	model-derived
2000	8000	2	0.88038	0.88029	0.88040	0.88025
2000	8000	5	0.88001	0.88001	0.88030	0.88025
2000	8000	10	0.88042	0.88003	0.88028	0.88025
2000	10000	2	0.90313	0.90296	0.90349	0.90337
2000	10000	5	0.90278	0.90301	0.90344	0.90337
2000	10000	10	0.90332	0.90364	0.90337	0.90337
4000	8000	2	0.77068	0.77084	0.77069	0.77078
4000	8000	5	0.77029	0.77085	0.77129	0.77078
4000	8000	10	0.77041	0.77062	0.77078	0.77078
4000	10000	2	0.81304	0.81327	0.81298	0.81332
4000	10000	5	0.81287	0.81330	0.81353	0.81332
4000	10000	10	0.81403	0.81362	0.81349	0.81332

Table S1: Comparison of simulated and model-derived singlet rates.

#Cells (Y)	#Droplets (X)	#Samples (M)	500 iterations	1000 iterations	1500 iterations	model-derived
2000	8000	2	0.06238	0.06258	0.06225	0.06242
2000	8000	5	0.09773	0.09758	0.09736	0.09742
2000	8000	10	0.10842	0.10890	0.10860	0.10870
2000	10000	2	0.05042	0.05014	0.04988	0.04996
2000	10000	5	0.07895	0.07869	0.07833	0.07836
2000	10000	10	0.08761	0.08728	0.08751	0.08757
4000	8000	2	0.12455	0.12417	0.12434	0.12436
4000	8000	5	0.18978	0.18924	0.18887	0.18940
4000	8000	10	0.21019	0.20976	0.20957	0.20967
4000	10000	2	0.10001	0.09959	0.09996	0.09967
4000	10000	5	0.15366	0.15332	0.15321	0.15329
4000	10000	10	0.16937	0.16994	0.17004	0.17022

Table S2: Comparison of simulated and model-derived MSM rates.

#Cells (Y)	#Droplets (X)	#Samples (M)	500 iterations	1000 iterations	1500 iterations	model-derived
2000	8000	2	0.05723	0.05712	0.05733	0.05732
2000	8000	5	0.02225	0.02239	0.02233	0.02232
2000	8000	10	0.01114	0.01105	0.01111	0.01104
2000	10000	2	0.04644	0.04688	0.04662	0.04666
2000	10000	5	0.01825	0.01828	0.01822	0.01826
2000	10000	10	0.00906	0.00907	0.00910	0.00904
4000	8000	2	0.10475	0.10497	0.10496	0.10485
4000	8000	5	0.03992	0.03990	0.03982	0.03980
4000	8000	10	0.01938	0.01961	0.01964	0.01954
4000	10000	2	0.08693	0.08712	0.08705	0.08699
4000	10000	5	0.03345	0.03336	0.03325	0.03337
4000	10000	10	0.01658	0.01643	0.01645	0.01644

Table S3: Comparison of simulated and model-derived SSM rates.

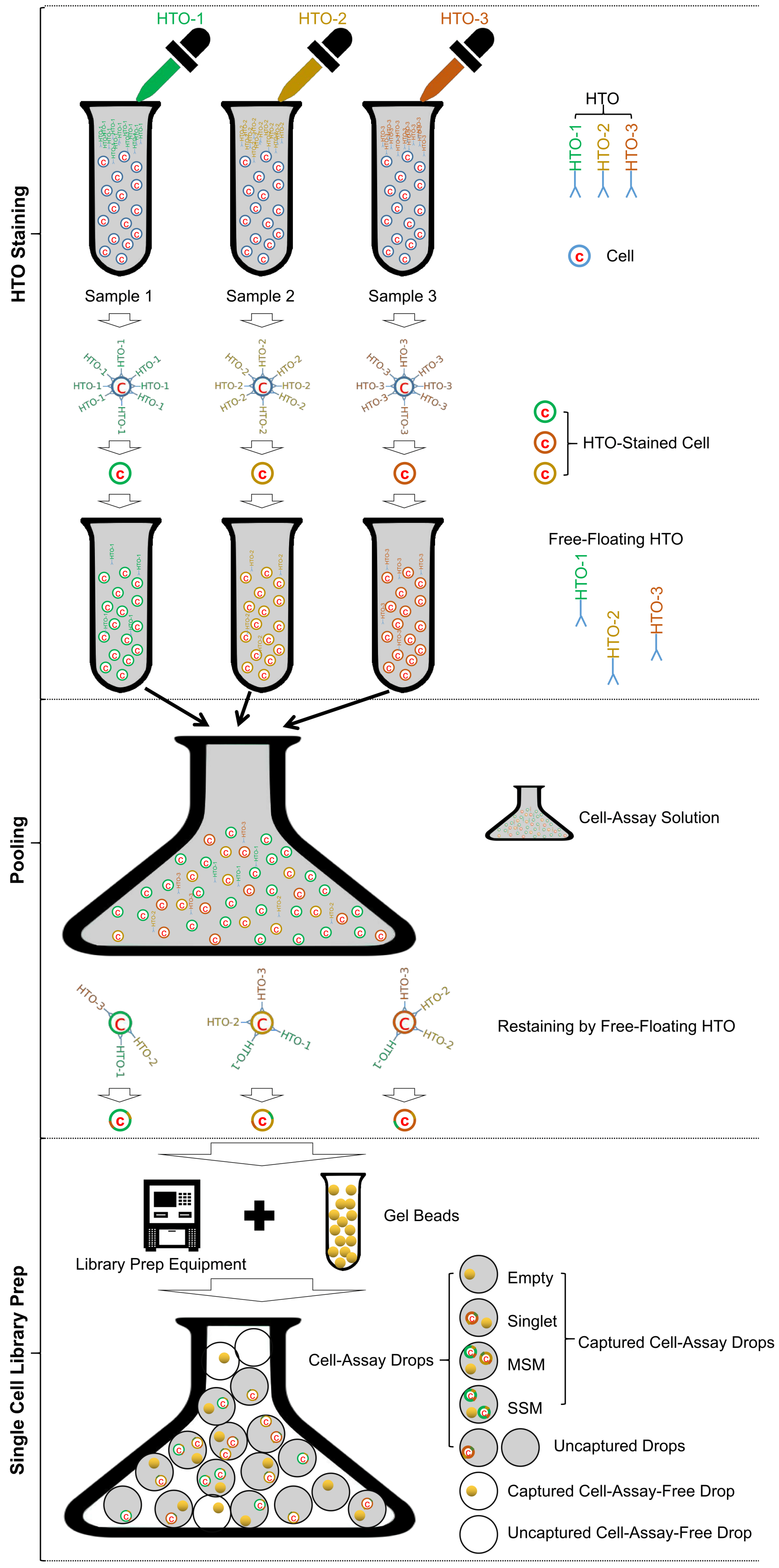

Figure S2: Sample barcoding workflow.

3 Hypothesis Testings in Pure-Type GEM Verification

To classify a GEM cluster G, GMM-Demux tests two hypotheses: G is a pure-type GEM cluster; and G is a phony-type GEM cluster. In both hypothesis tests, GMM-Demux first computes the expected value of $P(i \in \texttt{MSM}_\texttt{G}|i \in G)$—the probability of a GEM in G being a MSM, under the respective hypotheses. Then GMM-Demux computes the statistical significance of observing the true MSM population of G under the expected $P(i \in \texttt{MSM}_\texttt{G}|i \in G)$ derived from the previous step. GMM-Demux tests both hypotheses with the binomial test: let $P_0(i \in \texttt{MSM}_\texttt{G}|i \in G)$ denote the expected value of $P(i \in \texttt{MSM}_\texttt{G}|i \in G)$; GMM-Demux assumes that each GEM i in G has the probability of $P_0(i \in \texttt{MSM}_\texttt{G}|i \in G)$ to become a MSM, and computes the statistical significance (the p-value) of observing the true number of MSMs in G. The null hypotheses of the binomial test is rejected if the p-value of the test falls below a user defined alpha level (0.05 by default). According to the binomial test results of both hypotheses, G is classified as a pure-type GEM cluster, a phony-type GEM cluster or a mixture cluster accordingly.

GMM-Demux derives $P_0(i \in \texttt{MSM}_\texttt{G}|i \in G)$ from the GEM formation model. For simplicity, we assume cells are uniformly randomly distributed into M sample barcoding samples. Formally, the probability of a GEM i in G is a MSM can be formulated as the following:

$$P_0(i \in \texttt{MSM}_\texttt{G}|i \in G) = \sum_j P(i \in \texttt{MSM}_\texttt{G}|c_i = j) \cdot P(c_i = j) \tag{20}$$

where c_i denotes the number of cells in GEM i and $c_i = j$ states that there are j cells in i. If G is a phony-type cluster, since all GEMs are multiplets, $P(c_i = 1) = 0$. For $j > 1$, $P(i \in \texttt{MSM}_\texttt{G}|c_i = j) = 1 - P(i \in \texttt{SSM}_\texttt{G}|c_i = j) = 1 - \binom{1}{M}(\frac{1}{M})^j = 1 - (\frac{1}{M})^{j-1}$. Let k denote the number of cell types in a GEM. If G is a k-cell-type phony-type cluster, where each GEM in G contains (the same) k ($k > 1$) cell types, then $P_0(i \in \texttt{MSM}_\texttt{G}|i \in G) > 1 - (\frac{1}{M})^{k-1}$, as $P(c_i = j) = 0$ for all $j < k$. In general, let y_G represent the total cell population size of the k cell types of G in the cell assay. In a k-cell-type phony-type cluster, $P(c_i = j) = 0$ for all $j < k$ and $P(c_i = j) = \binom{y_G - k}{j-k}(\frac{1}{X})^{j-k}(1 - \frac{1}{X})^{k-j} \cdot P(c_i = k)$ for all $j \geq k$. When $X > y_G >> 1$ and $k > 1$, we have $P_0(i \in \texttt{MSM}_\texttt{G}|i \in G) \gtrapprox 1 - (\frac{1}{M})^{k-1}$.

The formula for $P_0(i \in \texttt{MSM}_\texttt{G}|c_i = j)$ is slightly different in more complex scenarios where the cell assay is not evenly distributed into the M sample barcoding samples. Assume cells are not uniformly randomly distributed into sample barcoding samples and let p_x denote the probability of a cell being distributed to a sample x. Then $P(i \in \texttt{SSM}_\texttt{G}|c_i = j) = \sum_{x=1}^{M} P(i \in \texttt{SSM}_\texttt{G}{}^x|c_i = j)$, where $P(i \in \texttt{SSM}_\texttt{G}{}^x|c_i = j)$ denotes the probability of a droplet i being a SSM of sample x, given that i already contains j cells. In other words, $P(i \in \texttt{SSM}_\texttt{G}{}^x|c_i = j)$ is the probability of all j cells in i come from sample x, which means $P(i \in \texttt{SSM}_\texttt{G}{}^x|c_i = j) = (p_x)^j$. Therefore, we have $P(i \in \texttt{SSM}_\texttt{G}|c_i = j) = \sum_{x=1}^{M}(p_x)^j$. Accordingly, $P_0(i \in \texttt{MSM}_\texttt{G}|i \in G)$ is updated as $P_0(i \in \texttt{MSM}_\texttt{G}|i \in G) \gtrapprox 1 - \sum_{x=1}^{M}(p_x)^k$ under the condition of $X > y_G >> 1$ and $k > 1$.

With $P_0(i \in \texttt{MSM}_\texttt{G}|c_i = j)$ computed for $k > 1$, the null and alternative hypotheses of the phony-type hypothesis test are formulated as follows: the null hypothesis states that $H_0^{phony} : P(i \in \texttt{MSM}_\texttt{G}|i \in G) \geq P_0(i \in \texttt{MSM}_\texttt{G}|i \in G)$, while the alternative

hypothesis states that $H_a^{phony} : P(i \in \texttt{MSM}_\texttt{G}|i \in G) < P_0(i \in \texttt{MSM}_\texttt{G}|i \in G)$. The null hypothesis is tested with the binomial test and is rejected if the p-value of the test is smaller than the alpha level. Rejecting the null hypothesis suggests that G is not a phony-type GEM cluster.

The phony-type hypothesis test can be further extended to infer the number of cell types (k) in G. For simplicity, again cells are assumed to be uniformly distributed into M sample barcoding samples, without loss of generality. For $k = 2$, $P_0(i \in \texttt{MSM}_\texttt{G}|i \in G) \gtrapprox 1 - \frac{1}{M}$. For $k = 3$, $P_0(i \in \texttt{MSM}_\texttt{G}|i \in G) \gtrapprox 1 - (\frac{1}{M})^2$. For $k = n$, $P_0(i \in \texttt{MSM}_\texttt{G}|i \in G) \gtrapprox 1-(\frac{1}{M})^{n-1}$. For each P_0, GMM-Demux performs the binomial test and rejects the null hypothesis when the p-value drops below the alpha value. Since $P_0(i \in \texttt{MSM}_\texttt{G}|i \in G)$ increases as k increases, rejecting the null hypothesis suggests that G is not a phony-type GEM cluster of no-less-than k cell types (but could still be a phony-type GEM cluster with fewer cell types).

Rejecting H_0^{phony} does not make G a pure-type GEM cluster. G could also be a mixture cluster, which contains both pure-type GEMs and phony-type GEMs. To check if G is a pure-type GEM only cluster, GMM-Demux performs the *pure-type hypothesis test* H^{pure}, which assumes that G includes only $k = 1$ cell type.

When $k = 1$, $P_0(i \in \texttt{MSM}_\texttt{G}|i \in G)$ is much smaller than $1 - \frac{1}{M}$. Let τ denote the cell type of G and assume that G includes all pure-type GEMs of type τ. Let y_τ denote the number of τ cells in the cell assay and z_G denote the number of GEMs in G. According to the GEM formation model, the probability of i being a pure-type GEM of type τ can be computed as $P(i \in SSD_\tau) = (1-(1-\frac{1}{X})^{y_\tau})(1-\frac{1}{X})^{Y-y_\tau}$. The probability of observing z_G pure-type GEMs of type τ equals to $P(\#_{\texttt{GEM}_\tau} = z_G) = \binom{z_G/r_{cap}}{X} P(i \in SSD_\tau)^{z_G/r_{cap}}(1 - P(i \in Pure_\tau))^{X-(z_G/r_{cap})}$. Consequently, GMM-Demux generates an estimation of y_τ by maximizing the likelihood of $P(\#_{\texttt{GEM}_\tau} = z_G)$.

With y_τ computed, the probability of i being a pure-type MSM of type τ, denoted as $P(i \in \texttt{MSM}_\tau)$, can be computed in a manner that is similar to Equation 10. Subsequently, we have $P_0(i \in \texttt{MSM}_\texttt{G}|i \in G) = \frac{P(i \in \texttt{MSM}_\tau) \cdot X}{z_G}$. The null and alternative hypotheses of the pure-type hypothesis test are formulated as $H_0^{pure} : P_{i \in MSM_\tau} \leq P(i \in \texttt{MSM}_\tau)_0$ and $H_a^{pure} : P(i \in \texttt{MSM}_\tau) > P(i \in \texttt{MSM}_\tau)_0$, respectively. GMM-Demux tests the null hypothesis with the binomial test; computes the p-value; compares the p-value against the alpha level and rejects H_0^{pure} if the p-value is smaller than the alpha level. Rejecting H_0^{pure} suggests that G is not a pure-type GEM cluster.

Based on the hypothesis testing results of both the pure-type and phony-type hypothesis tests, G is classified as a pure-type GEM cluster, a phony-type GEM cluster or a mixture cluster. Specifically, G is classified as a pure-type GEM cluster if H_0^{pure} is not rejected; G is classified as a phony-type GEM cluster if H_0^{phony} is not rejected; and G is classified as a mixture cluster if both H_0^{pure} and H_0^{phony} are rejected.

G has the potential to reveal a new cell type only if G is classified as a pure-type cluster. When classified as a phony-type cluster, all GEMs in G are recommended to be removed from the dataset, as all SSDs in G has a high probability to be SSMs. Finally, being classified as a mixture cluster suggests that G is not homogeneous and the clustering quality is low. Further refinement over G is recommended.

4 Example GMM-Demux Output

GMM-Demux generates three output files. It generates a classification result file, a classification header file, and a summary file. An example result and header file set is presented in Table S4 and S5. In the classification result file, each sample and each multi-sample combination is given a unique classification id. The classification id is matched with a description text in the header file. The summary file contains two tables, illustrated in Table S6 and S7. The first table presents a summary of the computed and inferred parameters of the dataset, including the number of cell-assay droplets (X), the capture rate (r_{cap}), the singlet, MSM and SSM rates of the entire dataset. The second table contains a per-sample summary, which stores the estimated SSM rate of each sample.

GEM Barcodes	Classification id	Confidence Score
AAACCTGAGACAAAGG-1	2	0.9999996
AAACCTGAGACAAGCC-1	3	0.9999854
AAACCTGAGAGGTTGC-1	3	0.9999995
AAACCTGAGAGTGAGA-1	5	0.9952781
AAACCTGAGCCCAACC-1	3	0.9999971
AAACCTGAGCTTATCG-1	7	0.9999966
AAACCTGAGGACAGAA-1	1	0.9999986
AAACCTGAGGGATCTG-1	3	0.9903349
AAACCTGAGGGATGGG-1	1	0.9995598
AAACCTGAGGGCACTA-1	3	0.5391441
AAACCTGAGGTTACCT-1	2	0.9999990
AAACCTGAGTACGATA-1	2	0.9991886
AAACCTGCAACACCCG-1	7	0.9998400
AAACCTGCAAGCTGTT-1	3	0.9999598
AAACCTGCAATCGAAA-1	0	0.9784425
AAACCTGCACCCTATC-1	4	0.9995443

Table S4: An example MSM classification result file.

Classification id	Classification Description
0	negative
1	hto1
2	hto2
3	hto3
4	hto1-hto2
5	hto1-hto3
6	hto2-hto3
7	hto1-hto2-hto3

Table S5: The header file of Table S4.

#Droplets (X)	r_{cap}	Est. Singlet	Obs. MSM	Est. SSM	Est. RSSM	Negative	Unclear
68480	56	76.11	18.05	5.25	6.45	0.47	2.71

Table S6: An example summary file for the entire dataset. Except the number of droplets (X), all other values are in %.

	hto1	hto2	hto3	hto4
# Cells	8982	8937	8746	9462
RSSM rate	6.4141	6.3828	6.2494	6.7489

Table S7: The per-sample summary of the example sample barcoding experiment. All RSSM values are in %.

5 Classification Results of the PBMC-2 and the Memory-T Datasets

MSM classification results of the PBMC-2 and the Memory-T datasets are included in Figure S3.

6 Distinct MSM Classification Results

Figure S4 shows the GMM-Demux classification results where each sample combination is given a distinct color. Notice that when the number of classes is large (e.g., the PBMC-2 sample), the color difference between classes become less noticeable to a naked eye.

7 Stability Analysis with Simulated Datasets

Figure S5 shows the stability benchmarks of GMM-Demux, multi-seq, demuxEM and Seurat. The stability is measured by computing the percentage of GEMs that changes classifications between repetitive runs. As shown in the figure, Seurat displays low stability while others classifiers produce consistent classifications between runs.

8 Online Experiment Planner Illustration

A screenshot of the online experiment planner is presented in Figure S6. To use the online experiment planner, the user provides the anticipated number of cells in the cell assay (Y), the number of HTO samples for sample barcoding (M), the estimated number of cell-assay droplets (X) and the estimated capture rate r_{cap} of the single cell library preparation equipment as inputs. The experiment planner outputs the estimated number of GEMs; the estimated singlet, MSM and SSM rates of GEM population; the estimated number of same-sample droplets (SSD) after removing MSMs using GMM-Demux; and the estimated RSSM rate of the remaining SSD population.

Both X and r_{cap} can be obtained from the SSM rate estimator by running GMM-Demux over an existing sample barcoding scRNA-seq dataset. We observe that X typically ranges between $65K$-$80K$ and r_{cap} typically varies between 0.4-0.6. In practice, we recommend profiling the library preparation equipment by first conducting a pilot sample barcoding scRNA-seq experiment and

The online experiment planner dynamically interacts with the user. It dynamically updates the outputs upon changes on input values. Given a fixed number of cells as well as a fixed equipment profile (a fixed number of cell-assay droplets and a fixed capture rate), by dragging the slider handle of the HTO-sample-number input (M), the user can observe updates on multiplet rates in real time and find the minimum number of HTO samples required to meet a specific RSSM rate target.

9 Multiplet Rate Profiling Results

The profiles of the singlet, MSM, SSM rates, as well as the estimated number of harvested GEMs, under varying cell populations (Y) and sample counts (M) are shown in Figure S7. Figure S7 is generated under the setting of 80K cell-assay droplets ($X = 80K$) and 0.5 capture rate ($r_{cap} = 0.5$). The above setting is based on the observation from Table 5.

As we have previously discussed, the singlet rate of an experiment, as well as the number of harvested gel-bead-enclosing GEMs only depend on the cell population size Y and are unaffected by the sample barcoding sample size M. Figure S7a and S7b displays the singlet rates and the estimated number of harvested GEMs under varying cell population sizes (Y). Between 1K to 40K, as more cells are loaded into the experiment, the singlet rate monotonically and super-linearly decreases, while the number of GEMs monotonically and sub-linearly increases.

The MSM and SSM rates, however, depend on both the cell population size Y and the sample number M. Under a fixed Y, a larger M transforms more SSMs into MSMs, although the margin quickly diminishes as M grows. On the other hand, under a fixed M, a larger Y leads to both greater MSM and greater SSM rates, in almost a linear fashion. The MSM and SSM rates under variate Ys and Ms are shown in Figure S7c and S7d, respectively.

The singlet rates reported in Figure S7a are consistent with findings from previous works. As reported in Zheng et al [49], the observed singlet rates are 98.4% for loading 2K cells and 96.9% for 6K cells, under the assumption of a 50% capture rate. In our work, according to the online experiment planner, under $X = 80K$ and $r_{cap} = 0.5$, the estimated singlet rate of $Y = 2K$ is 98.75% while the singlet rate of $Y = 6K$ is 96.3%.

Finally, we profile the minimum number of samples required to achieve certain RSSM rate targets under various cell population sizes (Y). Such profile helps in designing future sample barcoding experiments that minimize cost and complexity while meeting data quality targets. Figure S7f and S7e shows the profiling results of 1% and 5% RSSM rate targets, respectively, over various cell population sizes, again under the assumption of $X = 80K$ and $r_{cap} = 0.5$.

Singlet, MSM, SSM, RSSM rate profiles, as well as GEM count profiles under other settings ($[X, r_{cap}]$ other than $[80K, 0.5]$) are provided in Figure S8 to S13. Together, they serve as guidance in designing future sample barcoding experiments.

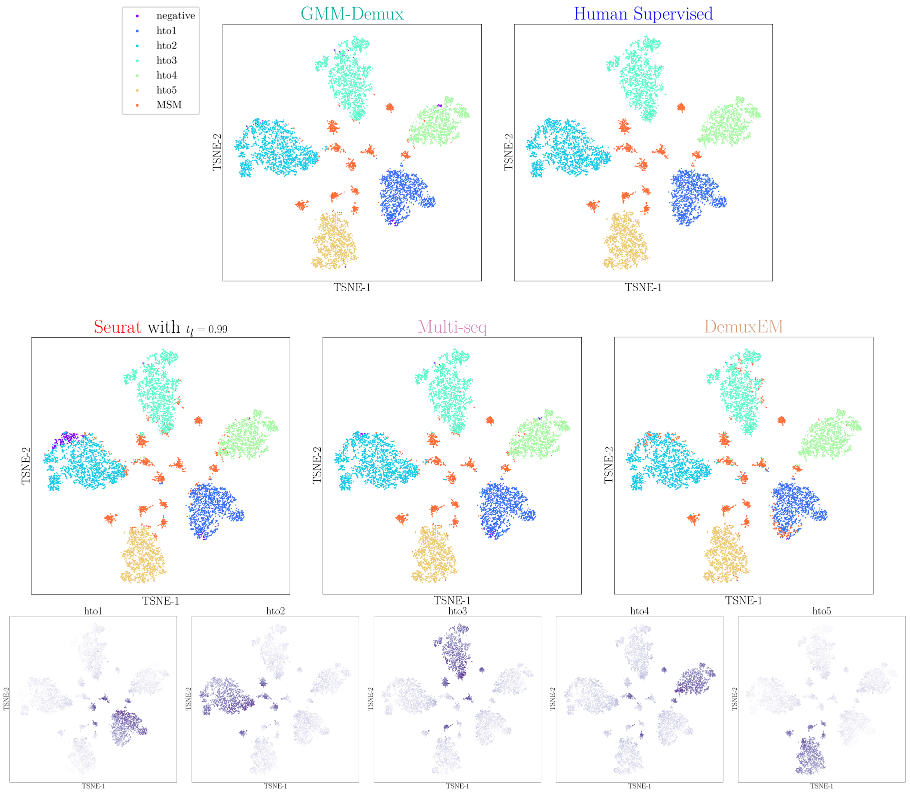

(a) Results for CD4$^+$ Memory T.

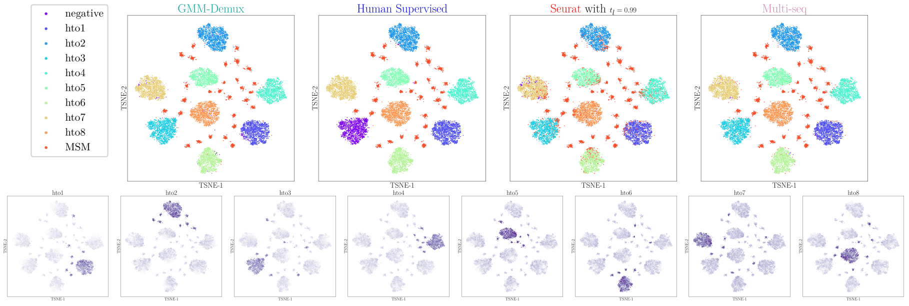

(b) Results for PBMC-2.

Figure S3: Classification results of the PBMC-2 and the Memory-T datasets. DemuxEM requires sample barcoding information of empty drops, which is not available in the PBMC-2 dataset. Hence we are not able to benchmark demuxEM on the PBMC-2 dataset.

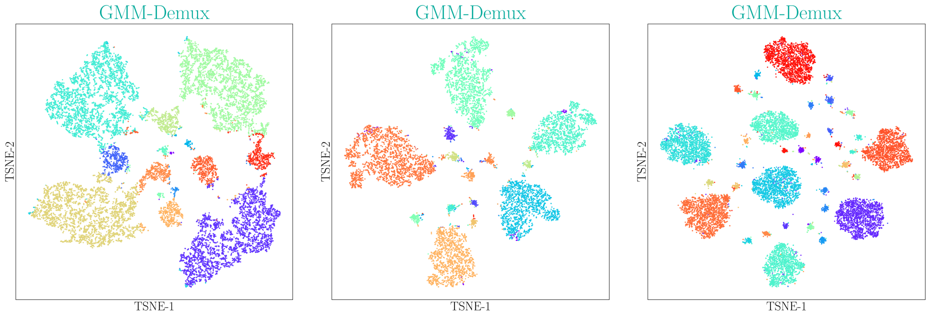

(a) PBMC-1 (b) CD4$^+$ Memory T (c) PBMC-2

Figure S4: GMM-Demux detailed classification results. Each sample and each multi-sample combination is given a unique color. As the number of classes grows, difference between colors becomes less noticeable.

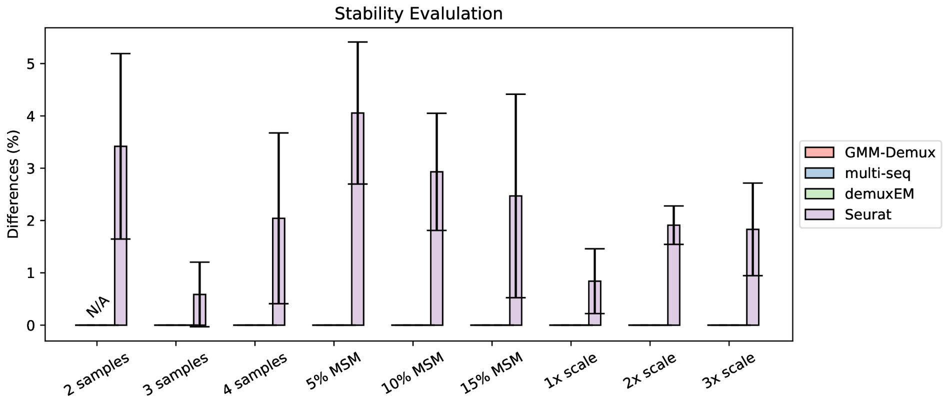

Figure S5: Stability comparison between sample barcoding classifiers on simulated datasets. Seurat is notably less stable than other classifiers.

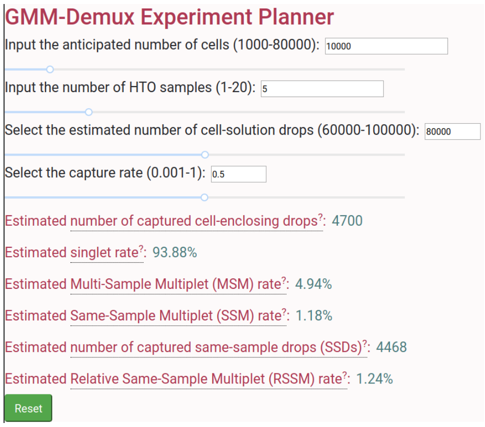

Figure S6: Screenshot of the online sample barcoding experiment planner. The experiment planner computes multiplet rates in real time as parameters are updated.

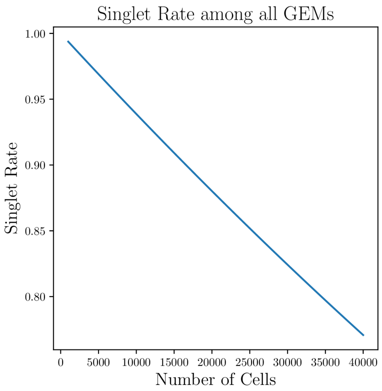

(a) Singlet rate profile.

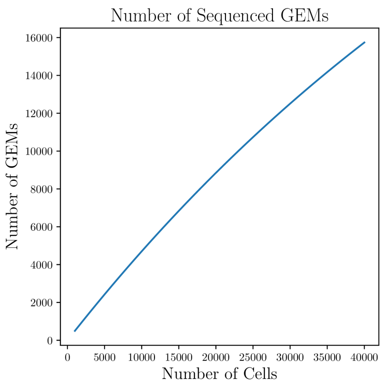

(b) GEM count profile.

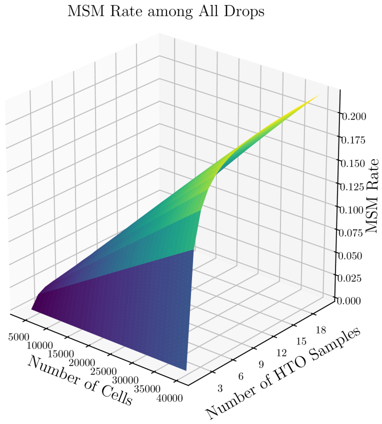

(c) MSM rate profile.

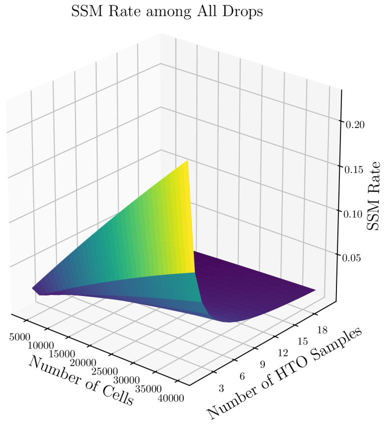

(d) SSM rate profile.

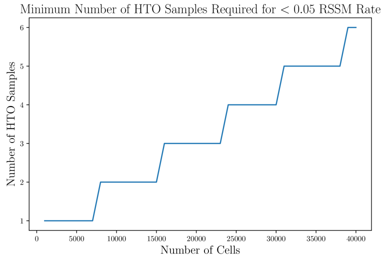

(e) Sample number profile for 5% RSSM rate target.

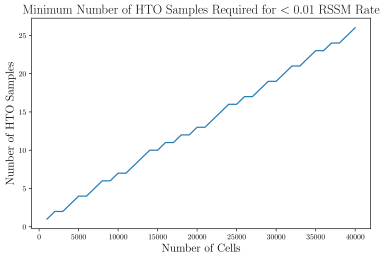

(f) Sample number profile for 1% RSSM rate.

Figure S7: Sample barcoding experiment profiles under the assumption of 80K cell-assay droplets ($X = 80$) and 0.5 capture rate ($r_{cap} = 50\%$). The singlet rate monotonically decreases as the cell population grows. Under a fixed cell population size, however, as the number of samples grows, more SSMs transit into MSMs.

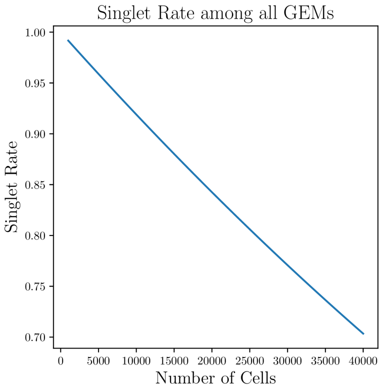

(a) Singlet rate profile.

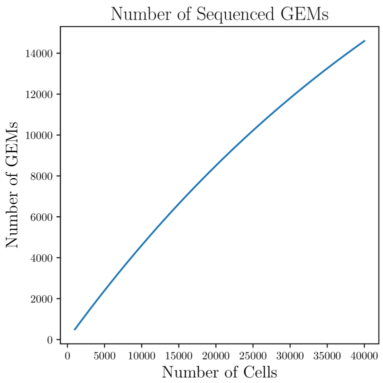

(b) GEM count profile.

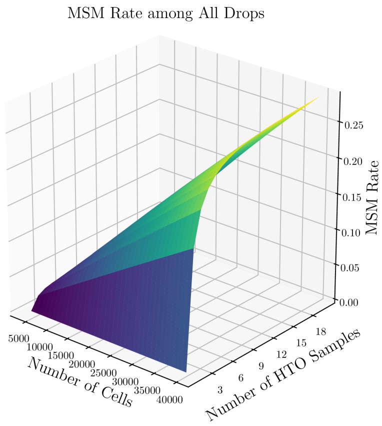

(c) MSM rate profile.

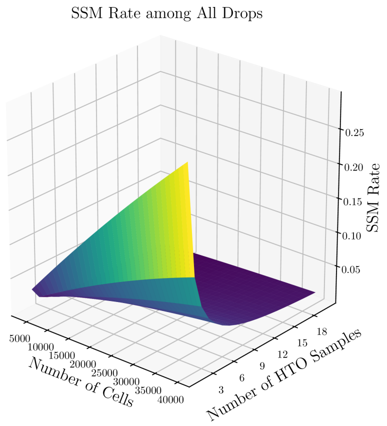

(d) SSM rate profile.

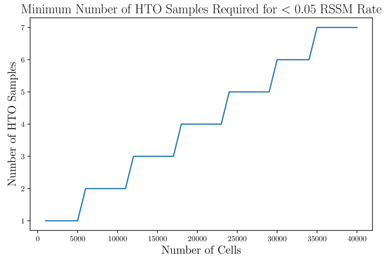

(e) Sample number profile for 5% RSSM rate target.

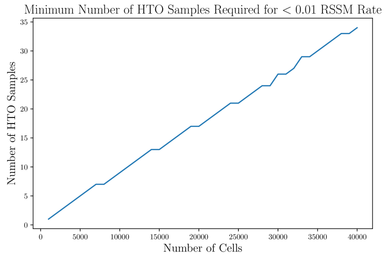

(f) Sample number profile for 1% RSSM rate.

Figure S8: Sample barcoding profiles under the assumption of 60K cell-assay droplets ($X = 60K$) and 0.5 capture rate ($r_{cap} = 50\%$).

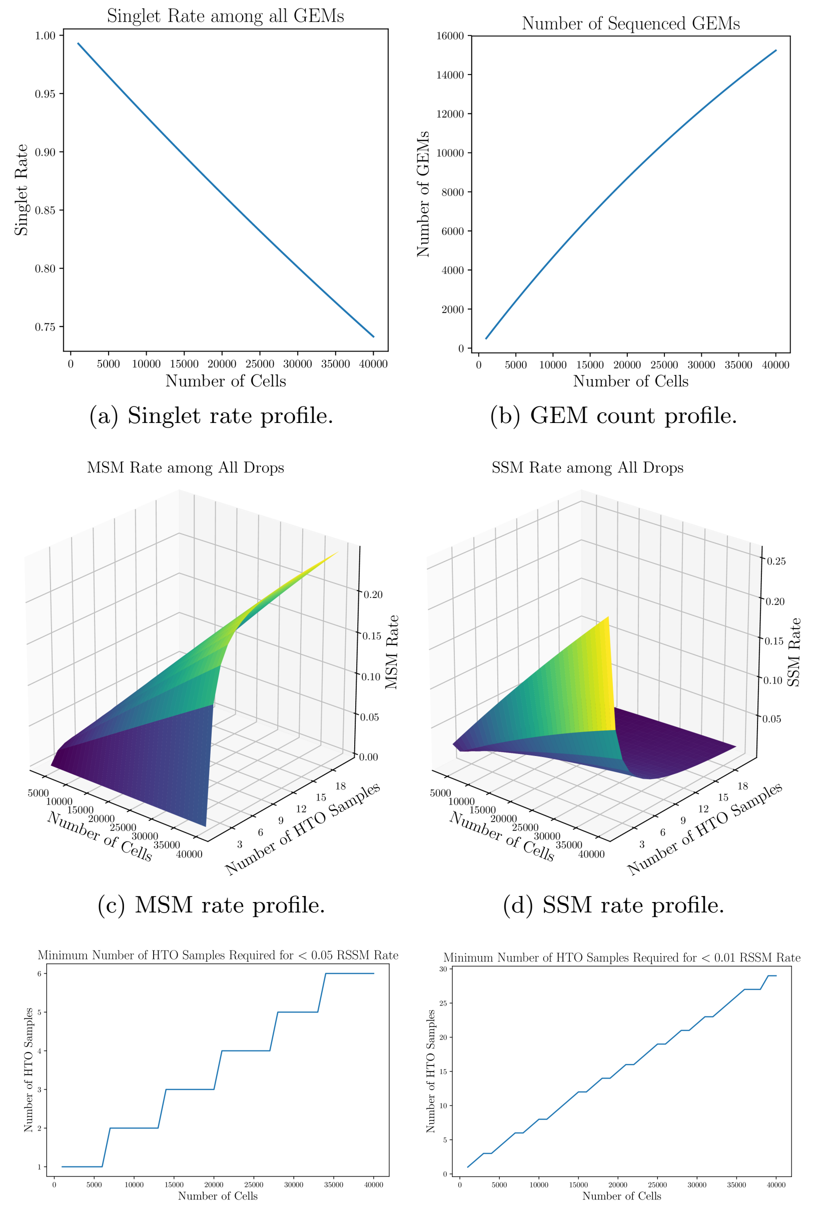

(a) Singlet rate profile.

(b) GEM count profile.

(c) MSM rate profile.

(d) SSM rate profile.

(e) Sample number profile for 5% RSSM rate target.

(f) Sample number profile for 1% RSSM rate.

Figure S9: Sample barcoding profiles under the assumption of 70K cell-assay droplets ($X = 70K$) and 0.5 capture rate ($r_{cap} = 50\%$).

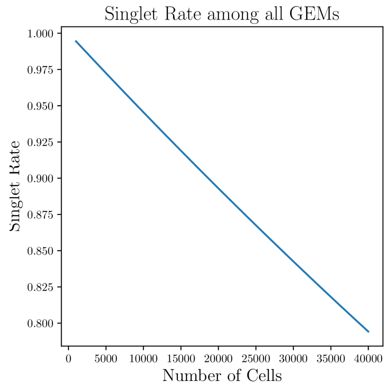

(a) Singlet rate profile.

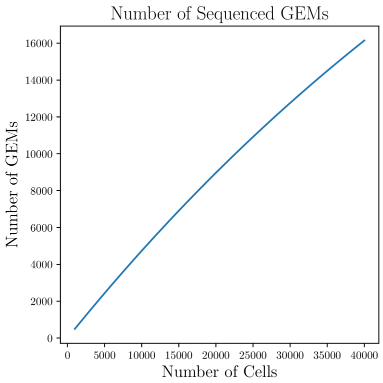

(b) GEM count profile.

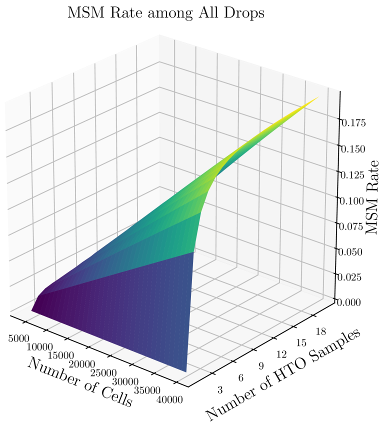

(c) MSM rate profile.

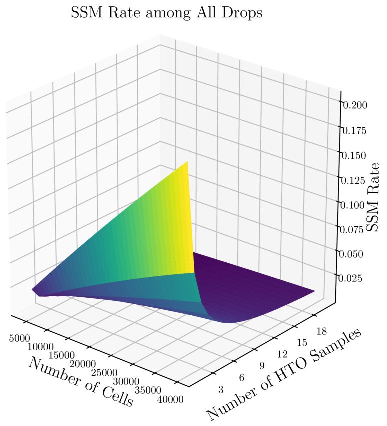

(d) SSM rate profile.

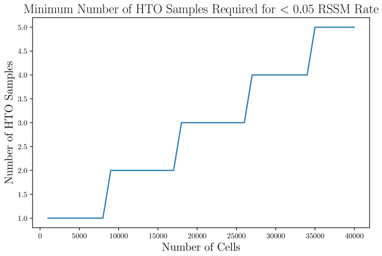

(e) Sample number profile for 5% RSSM rate target.

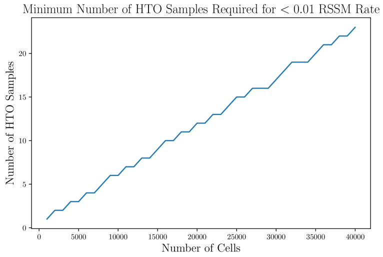

(f) Sample number profile for 1% RSSM rate.

Figure S10: Sample barcoding profiles under the assumption of 90K cell-assay droplets ($X = 90K$) and 0.5 capture rate ($r_{cap} = 50\%$).

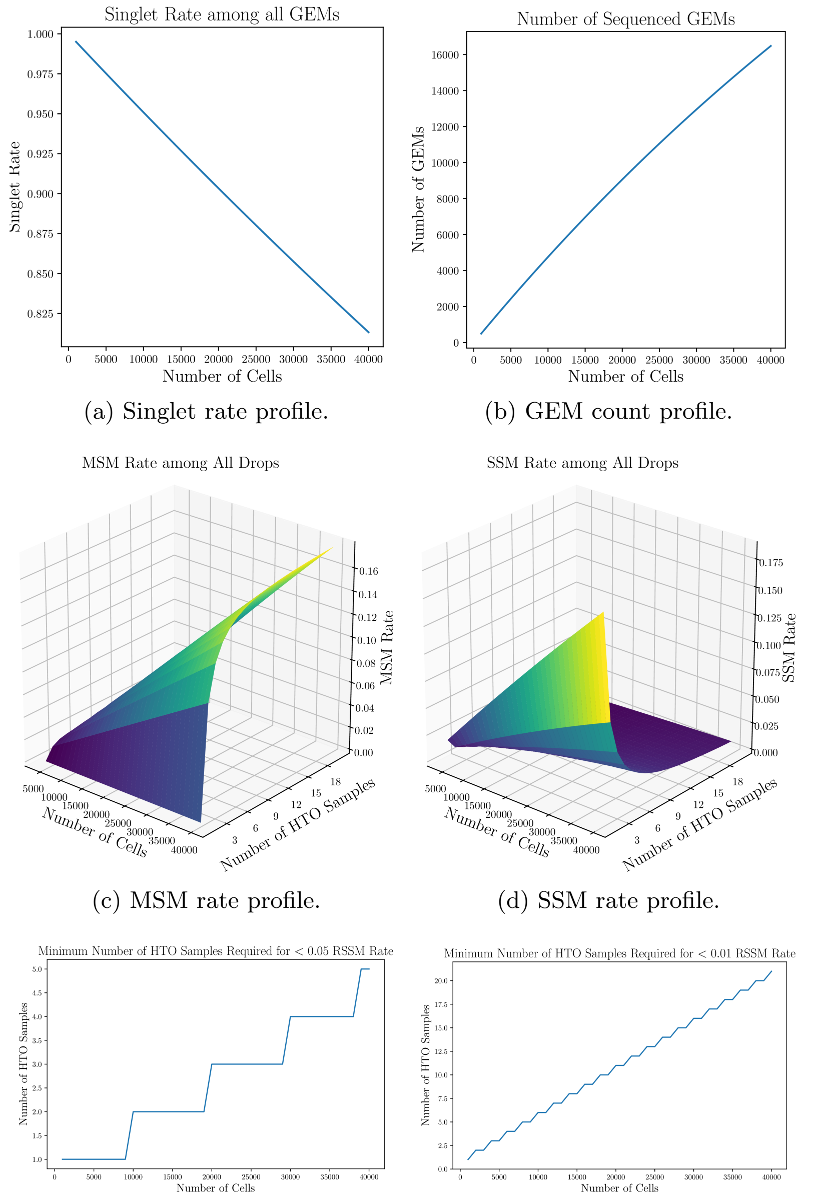

(a) Singlet rate profile.

(b) GEM count profile.

(c) MSM rate profile.

(d) SSM rate profile.

(e) Sample number profile for 5% RSSM rate target.

(f) Sample number profile for 1% RSSM rate.

Figure S11: Sample barcoding profiles under the assumption of 100K cell-assay droplets ($X = 100K$) and 0.5 capture rate ($r_{cap} = 50\%$).

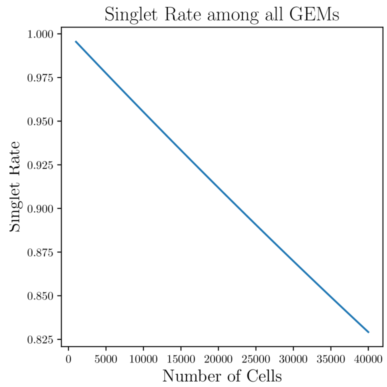

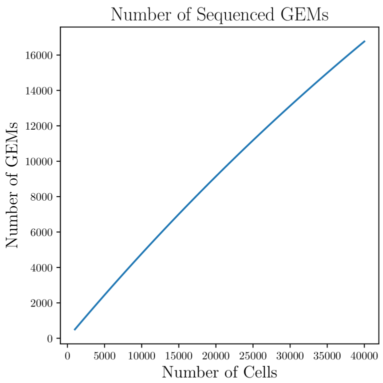

(a) Singlet rate profile. (b) GEM count profile.

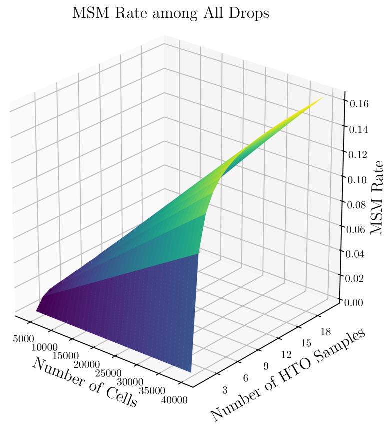

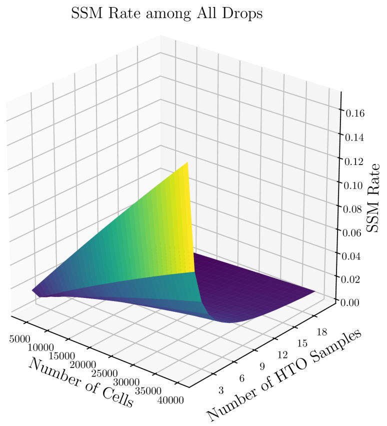

(c) MSM rate profile. (d) SSM rate profile.

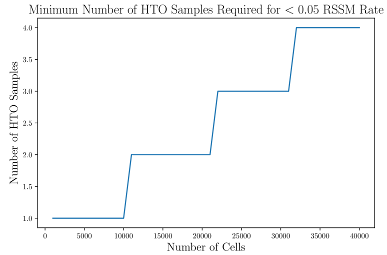

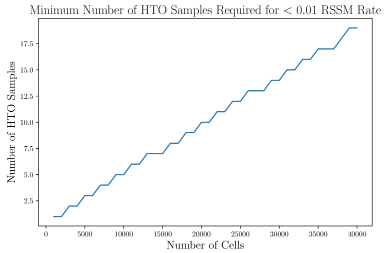

(e) Sample number profile for 5% RSSM rate target. (f) Sample number profile for 1% RSSM rate.

Figure S12: Sample barcoding profiles under the assumption of 110K cell-assay droplets ($X = 110K$) and 0.5 capture rate ($r_{cap} = 50\%$).

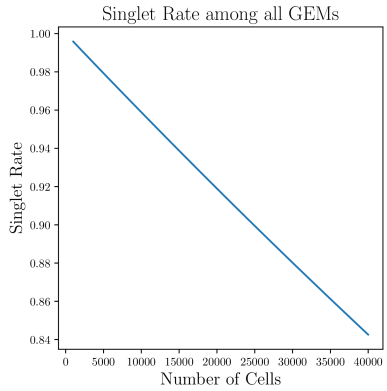

(a) Singlet rate profile.

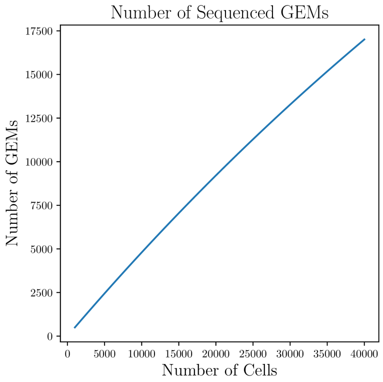

(b) GEM count profile.

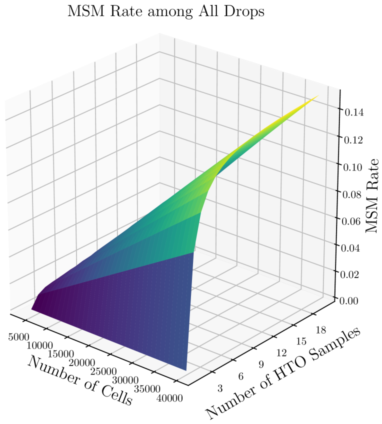

(c) MSM rate profile.

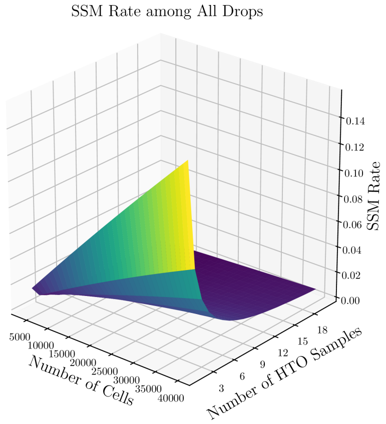

(d) SSM rate profile.

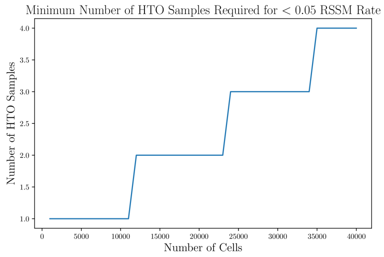

(e) Sample number profile for 5% RSSM rate target.

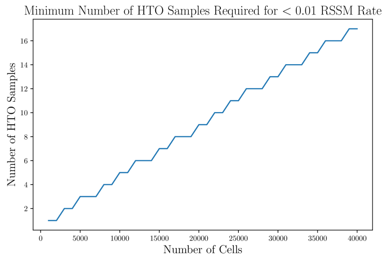

(f) Sample number profile for 1% RSSM rate.

Figure S13: Sample barcoding profiles under the assumption of 120K cell-assay droplets ($X = 120K$) and 0.5 capture rate ($r_{cap} = 50\%$).

10 Confidence Score Distributions

Figure S14 shows the confidence score distributions of the three cell hashing datasets presented in Table 2. As shown in the figure. Most droplets (> 95%) have confidence scores above 0.8.

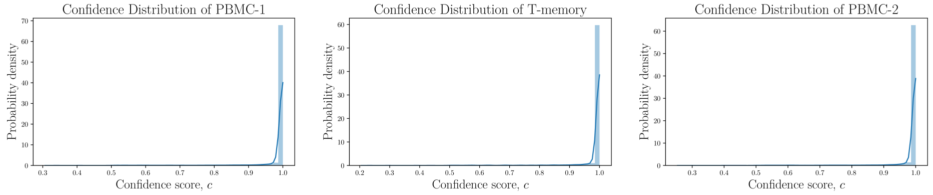

Figure S14: Confidence Score Distributions.

11 Background HTO Antibody Distributions

Figure S15 displays the CLR-normalized per-sample HTO antibody count distributions in the PBMC-1 dataset, including both GEMs and empty droplets. If the background HTO antibody assumption of demuxEM is correct, where HTO antibodies come from either sample-staining antibodies or free-floating antibodies in the suspension, then we should observe only two Gaussian components in each distribution. However, as shown in all plots, there exist more than two Gaussian components. This proves that free-floating antibodies in the GEM suspension is not the only source of background HTO antibodies and the assumption of demuxEM is inconclusive.

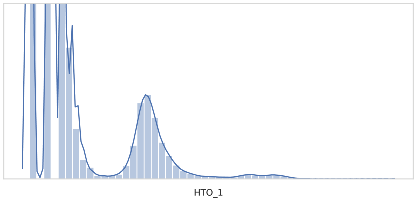

(a) Sample 1.

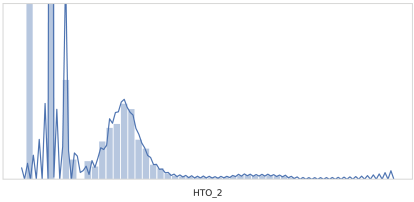

(b) Sample 2.

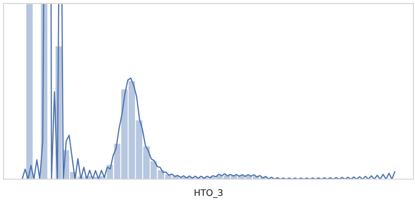

(c) Sample 3.

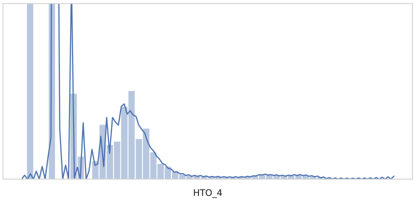

(d) Sample 4.

Figure S15: Distributions of CLR-transformed HTO UMI counts of the PBMC-1 dataset, with empty droplets included.

12 Parameter Selections for DoubletFinder and Scrublet

We applied both DoubletFinder and scrublet to the PBMC-1 dataset with default parameters. For scrublet, we did not specify any parameters to the

`scrub_doublet()` function. For DoubletFinder, we used the following parameters: `PCS = 1:10`, `pN = 0.25`, `pK = 0.01` (as suggested in the manuscript), and we assumed a doublet formation rate of 0.125 (we assumed that the DoubletFinder is agnostic to the sample-barcoding information and does not know the total multiplet rate). We used the Seurat clustering result to derive the pure-type multiplet rate and then calculated the phony-type multiplet rate accordingly, following the instructions included in the DoubletFinder manuscript.

13 Impact of Phony-Type GEMs in Downstream Analysis

We use scanpy[44] to benchmark the impact of phony-type GEMs in downstream scRNA-seq analysis. We compare the RNA-based clustering results of the PBMC-1 dataset with and without removing phony-type GEMs. Both clustering results are visualized in UMAP plots in Figure S16, with their respective surface-marker-based annotations on the side as references. As shown in Figure S16A, without removal of phony-type GEMs, the RNA clustering result is less in accordance with the surface marker annotation result (Figure S16B). Cluster 6, in particular, has a MSM percentage of 51.57%. Further decomposition of cluster 6 (Figure S16E) shows that it consists GEMs of multiple types, of which 19.8% are phony-type GEMs. After removal of phony-type GEMs, however, the RNA-based clustering result (Figure S16C) is a lot similar to the surface marker annotation result (Figure S16D). The AMI scores of both RNA clustering results, measured by comparing against the surface marker annotation, with and without removal of phony-type GEMs, are 0.72 and 0.53, respectively. Note that we only measure the AMI scores against the pure-type GEMs. We remove all phony type GEMs prior to computing the AMI scores in both cases. A higher AMI score indicates that removing phony type GEMs creates better clusters of pure-type GEMs that are more in accordance with their surface marker annotations. Overall, we conclude that phony-type GEMs can confound downstream scRNA-seq analysis and their subsequent removal is critical to the integrity of downstream scRNA-seq analysis.

14 MSM Estimation Result for PBMC-2

The comparison between observed and model-derived MSM counts for the PBMC-2 dataset is summarized in Table 8 (provided separately). Similar to the PBMC-1 and the $CD4^+$ Memory T datasets, the observed counts and model-derived counts are highly consistent for each and every SSD and MSM category.

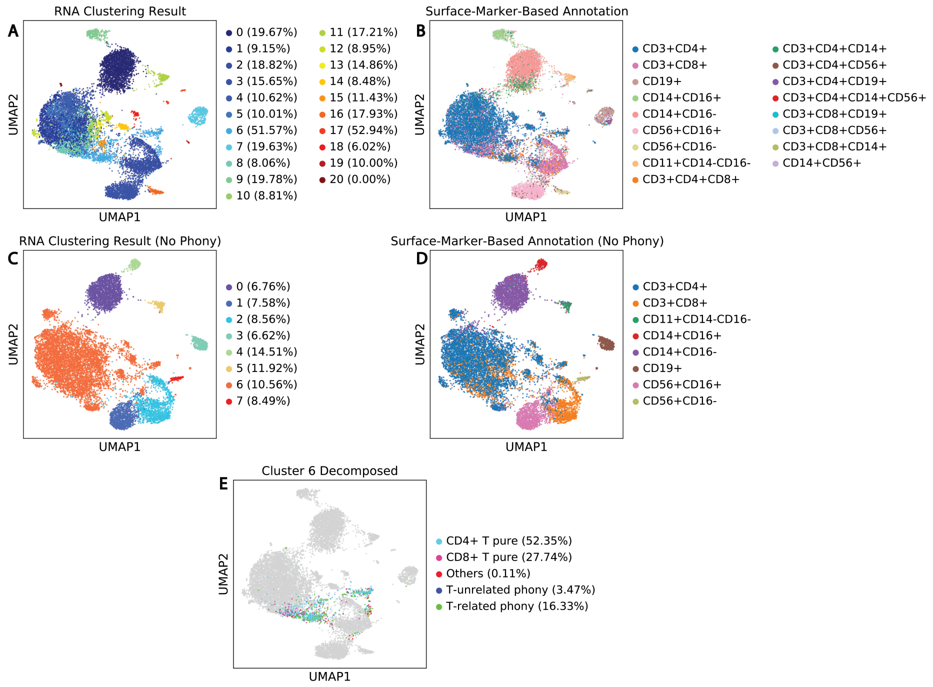

Figure S16: Impact of phony-type GEMs in downstream RNA analysis. Figure A illustrates the clustering result of the RNA data of the PBMC-1 dataset. Included in the parenthesis are MSM percentages of the cluster. Figure B shows the cell type annotations of each GEM according to their surface marker profiles. Figure C shows the clustering result of the RNA data of just the pure-type GEMs. Figure D depicts the cell type annotation of each GEM in Figure C, using the surface marker data. Figure E shows the composition of cluster 6 in Figure A, according to the surface marker data.
