# Supplementary material for: GMM-Demux: sample demultiplexing, multiplet detection, experiment planning, and novel cell-type verification in single cell sequencing
Source: Genome Biol. 2020 Jul 30;21:188. doi: 10.1186/s13059-020-02084-2 (PMC7393741; doi:10.1186/s13059-020-02084-2)
Supplement: Supplementary file 3 — Additional file 3 Review history [file 13059_2020_2084_MOESM3_ESM.docx]

Review History

**First round of review**

**Reviewer 1**

**Were you able to assess all statistics in the manuscript, including the appropriateness of statistical tests used?**

Yes: Algorithm methods and associated code were sufficiently documented

**Were you able to directly test the methods?**

No

**Comments to author:**

Summary

The authors describe GMM-Demux - a computational pipeline for processing sample-multiplexed scRNA-seq data. The authors demonstrate that GMM-Demux (i) identifies doublets and singlets, (ii) estimates the proportion of undetected doublets, and (iii) formalizes the identification of artifactual cell clusters according to the proportion of cells in each cluster that were classified as doublets. The authors begin by proposing a conceptual framework for classifying droplets according to (i) HTOs (e.g., MSMs, SSMs, or singlets) and (ii) cell types (e.g., pure-type, phony-type, or mixed). The authors then benchmark GMM-Demux against the Seurat heuristic classifier on three sample-multiplexed scRNA-seq and CITE-seq datasets as well as simulated data. Finally, the authors illustrate how doublet classifications can be used to identify putative artifactual cell clusters grouped according to surface protein abundances measured using CITE-seq.

It is evident that GMM-Demux is a useful tool for processing sample multiplexed scRNA-seq data. However, it remains unclear how GMM-Demux performance compares to other existing sample demultiplexing algorithms (see Major Comment #1), which will make it difficult for the single-cell genomics community to make an informed choice about how to analyze their data. Moreover, considering the abundance of alternative experiment planning tools and sample demultiplexing algorithms, it is clear that the current manuscript presents two key innovations: (i) a model for the proportion of undetected doublets (e.g., SSMs) and (ii) a formal framework for using GMM-Demux classification results to parse GEM clusters into 'phony' and 'pure' subsets. I believe that the authors should employ existing doublet detection methods (e.g., DoubletFinder and Scrublet) as well as the actual gene expression data (i.e., RNA UMI counts instead of solely relying on HTO and CITE-seq counts) to validate both of these GMM-Demux features.

For example, it is established that sample multiplexing approaches will identify doublets to which computational doublet detection techniques (e.g., DoubletFinder and Scrublet) are insensitive. However, doublet detection techniques are able to detect the subset of SSMs formed from cells that are transcriptionally distinct. Could this fact be incorporated to better improve SSM estimations and, as a result, experiment planning (see Major Comment #3)? Moreover, could the authors use these doublet detection techniques along with standard gene expression metrics (e.g., cluster identity, number of detected genes, total RNA or HTO UMIs, etc.) to further validate their identification of pure/mixed/phony GEM clusters beyond what they have shown for just a handful of surface protein abundances (see Major Comment #4)? Gene expression data (e.g., RNA counts) are available for most of the datasets presented in the paper but are not used once!

Major Comments

1. The authors successfully benchmark GMM-Demux against the Seurat heuristic classifier. However, the authors' claims that "the heuristic classifier currently is the only analytical tool for processing sample barcoding data" and that "GMM-Demux is the first work that is able to… accurately and consistently classify sample-barcoding-detectable MSMs in a sample barcoding dataset" are incorrect and must be removed prior to publication.

In fact, the poor performance of the Seurat demultiplexing algorithm was previously shown in multiple publications and a pre-print describing alternative algorithms for sample demultiplexing and doublet detection (e.g., deMULTIplex: McGinnis et al., 2019, Nature Methods; demuxEM: Gaublomme et al., 2019, Nature Communications; Solo: Bernstein et al., 2019, bioRxiv). The authors must refer to these methods explicitly and benchmark the performance of GMM-Demux to each method. Comparing to GMM-Demux to demuxEM is especially critical, as this approach is conceptually identical to GMM-Demux and requires an explicit explanation of how the two methods differ.

2. While the tool itself seems useful, I do not see the utility of including the online experiment planning resource in the Results section of the current manuscript, especially considering that similar experiment planning tools already exist.

3. The authors' claim that existing doublet detection methods which leverage gene expression information (e.g., DoubletFinder and Scrublet) cannot "reliably distinguish multiplets from transitional cells" runs counter to evidence described in the associated publications (e.g., DoubletFinder: McGinnis, Murrow & Gartner, 2019, Cell Systems; Scrublet: Wolock, Lopez & Klein, 2019, Cell Systems). Moreover, while it is true that DoubletFinder and Scrublet cannot identify doublets formed from transcriptionally-similar cells, other doublet detection algorithms have the potential to address this limitation (e.g., Wainer-Katsir & Linial, 2019, bioRxiv). The authors must either definitively prove these claims or remove them from the manuscript. More generally, it could be useful to explore how GMM-Demux can interface constructively with these existing methods (e.g., to improve SSM modeling and detection of SSMs, experiment planning, etc.), instead of just ignoring their existence.

4. Many of the authors' arguments rely on the notion that CD4/8 double-positive T-cells are too rare to be seen in this study. While it is very possible that these cells are indeed doublets, I think many would argue that they are not "non-existent" in PBMC samples and that this is not "conventional wisdom." I suggest that the authors utilize the gene expression data (e.g.. not just CITE-seq gating) to discern whether there are different clusters of CD4/8 double-positive T-cells that can be linked to singlet or doublet classifications (e.g., by looking at results from computational doublet detection techniques, total numbers of RNA and/or HTO UMIs, etc.). Leveraging the gene expression data would additionally improve the classification of 'mixed' GEM clusters (e.g., CD14+CD16+ non-classical monocytes), which the authors unsatisfactorily discuss due to their limited interpretation of CITE-seq gates.

**Reviewer 2**

**Were you able to assess all statistics in the manuscript, including the appropriateness of statistical tests used?**

Yes

**Were you able to directly test the methods?**

Yes

**Comments to author:**

Xin et al present a tool (GMM-Demux) to accurately identify sample-barcoding-detectable multiplets and estimate the proportion of sample-barcoding-undetectable multiplets. The authors apply their method to real and simulated data sets. Additionally, the authors provide a freely available online experiment planner to help guide future experiments. While the tool seems to do what it is supposed to, I recommend that the authors demonstrate that their tool solves a significant problem and enables novel biological insight to motivate broad utility. Please find a more detailed list of concerns below:

Major:

Methodologically the tool seems sound. However, in the current format it is difficult to judge how big of a problem GMM-Demux solves. Does the contamination of phony GEMs have any grave downstream effects? It is not clear why I need to care about them. I expect that emphasizing this point would increase utility of the tool.

For example, can the phony GEMs lead to erroneous discovery of novel cell types? The authors try to address this question in Section 5.5., where the authors illustrate the use of GMM-Demux by analyzing the PBMC-1 dataset. I find this exemplary analysis rather constructed. The idea of CITE-seq is to profile BOTH protein and RNA simultaneously. However, the authors use a protein-only gating strategy to derive cell types and ignore the RNA information completely. I wonder if the derived 17 GEM clusters would also appear when performing standard scRNAseq analysis on the RNA data. Do these phony GEM clusters (i.e. CD3+CD4+CD14+) actually produce separate clusters in the scRNAseq data? If not, then I am not sure those would be considered "novel candidate cell types" and the contamination seems to be rather subtle.

Can the phony GEMs corrupt cell type expression profiles? The authors should evaluate if cell type marker profiles are impacted and compare marker gene lists before and after removing phony clusters.

It is not clear how big the improvement over existing methods is. I appreciate the fact the authors used a "Human Supervised" approach for comparison. On first sight the differences between the methods seem to be very small. What could be the downstream effects? The authors should also compare the heuristic method in their analysis in Section 5.5 or additional analyses as suggested.

Fig 6 - it is hard to evaluate stability by looking at a few (manually selected?) tSNE plots. The authors should perform a systematic analysis to evaluate stability. For example, show the variance of the heuristic classification across varying tl values and sub-sampling scenarios.

Fig 7 - The authors should expand the simulation analysis by testing multiple scenarios. Is the 69x improvement a stable result?

Fig 8 - Non-proportionate Venn diagrams are a very misleading visualization. The authors should compare the estimated and observed proportion using a different visualization (maybe scatter plot?). Can the authors statistically evaluate significance of the agreement?

The authors should comment on runtime.

Minor:

The manuscript would benefit from shortening. Fig 1 is essentially a remake of Fig 1A from Stoeckius et al and not necessary. Fig 4 is informative but all 4 plots look the same and could benefit from possible compression.

Consistent font should be used across all equations

p.10 "This idea can be further expended into artificially create..."

The Github repo is exceptionally well documented but includes minor typos which could be fixed by another thorough readthrough.

**Authors Response**

**Point-by-point responses to the reviewers’ comments:**

Reviewer #1:

Summary: The authors describe GMM-Demux - a computational pipeline for processing sample-multiplexed scRNA-seq data. The authors demonstrate that GMM-Demux (i) identifies doublets and singlets, (ii) estimates the proportion of undetected doublets, and (iii) formalizes the identification of artifactual cell clusters according to the proportion of cells in each cluster that were classified as doublets. The authors begin by proposing a conceptual framework for classifying droplets according to (i) HTOs (e.g., MSMs, SSMs, or singlets) and (ii) cell types (e.g., pure-type, phony-type, or mixed). The authors then benchmark GMM-Demux against the Seurat heuristic classifier on three sample-multiplexed scRNA-seq and CITE-seq datasets as well as simulated data. Finally, the authors illustrate how doublet classifications can be used to identify putative artifactual cell clusters grouped according to surface protein abundances measured using CITE-seq.

It is evident that GMM-Demux is a useful tool for processing sample multiplexed scRNA-seq data. However, it remains unclear how GMM-Demux performance compares to other existing sample demultiplexing algorithms (see Major Comment #1), which will make it difficult for the single-cell genomics community to make an informed choice about how to analyze their data. Moreover, considering the abundance of alternative experiment planning tools and sample demultiplexing algorithms, it is clear that the current manuscript presents two key innovations: (i) a model for the proportion of undetected doublets (e.g., SSMs) and (ii) a formal framework for using GMM-Demux classification results to parse GEM clusters into 'phony' and 'pure' subsets. I believe that the authors should employ existing doublet detection methods (e.g., DoubletFinder and Scrublet) as well as the actual gene expression data (i.e., RNA UMI counts instead of solely relying on HTO and CITE-seq counts) to validate both of these GMM-Demux features.

For example, it is established that sample multiplexing approaches will identify doublets to which computational doublet detection techniques (e.g., DoubletFinder and Scrublet) are insensitive. However, doublet detection techniques are able to detect the subset of SSMs formed from cells that are transcriptionally distinct. Could this fact be incorporated to better improve SSM estimations and, as a result, experiment planning (see Major Comment #3)? Moreover, could the authors use these doublet detection techniques along with standard gene expression metrics (e.g., cluster identity, number of detected genes, total RNA or HTO UMIs, etc.) to further validate their identification of pure/mixed/phony GEM clusters beyond what they have shown for just a handful of surface protein abundances (see Major Comment #4)? Gene expression data (e.g., RNA counts) are available for most of the datasets presented in the paper but are not used once!

*Response to summary: We thank the reviewer for the summary. We greatly appreciate the enthusiasm from the reviewer and the constructive recommendations. The reviewer accurately and concisely summarized the contribution of our work. Indeed, a major contribution of our work is that we proposed a classical probability model to describe the droplet formation process and that we showed evidence proving the correctness of the model. This is also the key contribution that separates our work from existing demultiplexing methods. The reviewer also made very insightful observations that transcriptomic data has the potential to further improve the classification of cell types.*

*In fact, we initially planned to include the transcriptomics analysis in the manuscript at the time of writing. However, deterred by the inconsistent and sometimes very low correlations between mRNA and protein expressions (Haider & Pal, 2013) and that surface protein markers are often considered more reliable and are widely used to annotate the ground truth in cell type classification (Abdelaal, et al., 2019), we decided to drop the transcriptomics analysis and proceed with just the surface marker data for the purpose of sample demultiplexing and multiplet detection. Additionally, since GMM-Demux focuses on cell type authentication rather than cell type classification, and the performance of the cell type authentication analysis partially depends on the accuracy of the clustering result, we intentionally took a supervised clustering approach in the surface marker space.*

*In the version, following reviewer’s suggestion, we have supplied additional transcriptomics analysis to complement our primary analysis. We show that (1) the phony-type and pure-type classification of GEM clusters are consistent with RNA-based doublet classifications and (2) there exist limitations in finding doublets with RNA data, especially when searching for doublets of biologically similar cells.*

Major Comments

Comment (C)1. The authors successfully benchmark GMM-Demux against the Seurat heuristic classifier. However, the authors' claims that "the heuristic classifier currently is the only analytical tool for processing sample barcoding data" and that "GMM-Demux is the first work that is able to... accurately and consistently classify sample-barcoding-detectable MSMs in a sample barcoding dataset" are incorrect and must be removed prior to publication.

In fact, the poor performance of the Seurat demultiplexing algorithm was previously shown in multiple publications and a pre-print describing alternative algorithms for sample demultiplexing and doublet detection (e.g., deMULTIplex: McGinnis et al., 2019, Nature Methods; demuxEM: Gaublomme et al., 2019, Nature Communications; Solo: Bernstein et al., 2019, bioRxiv). The authors must refer to these methods explicitly and benchmark the performance of GMM-Demux to each method. Comparing to GMM-Demux to demuxEM is especially critical, as this approach is conceptually identical to GMM-Demux and requires an explicit explanation of how the two methods differ.

*Response to C1: We thank the reviewer for suggesting these compelling tools. At the time of finishing our first draft, neither DemuxEM (Gaublomme, et al., 2019) nor MULTI-seq (McGinnis, et al., 2019) was published. We apologize for this oversight. We have subsequently removed the statement from the manuscript. Additionally, following the reviewer’s suggestion, we have benchmarked GMM-Demux against demultiplex (MULTI-seq) and demuxEM. We have included their results in the Result section (page 6-8). Below is a short summary of the benchmark results.*

*We generated 9 of simulated cell hashing datasets using the single-sample droplets from the four samples of the PBMC-1 dataset. Below is the summary of the 9 simulated datasets.*

*We benchmarked GMM-Demux against Seurat, demuxEM and MULTI-seq. We measured the AMI score (Vinh, Epps, & Bailey, 2010) of each MSM classification result against the simulation ground truth. Below shows the AMI score benchmark result.*

*As shown in the above figure, GMM-Demux achieves high accuracy across all simulation settings, while other methods have reduced AMI scores when the number of samples is small (2 samples) or when samples have imbalanced cell populations (2x and 3x scales). Note that MULTI-seq failed to complete processing the 2-sample dataset, as it exited with error messages.*

*The reviewer is keen at noticing the similarity between demuxEM and GMM-Demux. Both methods assume that the HTO antibody count of a GEM come from two sources: background antibodies and sample staining. However, the two methods differ from modeling the background HTO counts. GMM-Demux models the background HTO antibodies as free-floating antibodies that rebind to cell surfaces during pooling while demuxEM models background antibodies as non-binding free-floating antibodies that are encapsulated in all emulsions, including the empty droplets. As a result, demuxEM takes unfiltered scRNA-seq data and extracts the background HTO antibody distribution from the empty droplets. GMM-Demux, on the other hand uses filtered data (post empty-droplet removal) and builds the background antibody distribution using Gaussian Mixture Models.We find that the background antibody assumption of demuxEM is not reliable. Below shows the antibody distribution of unfiltered data (also in the Supplementary Materials page 57). If the assumption of demxEM is correct, then we should observe two Gaussian components. However, this is not confirmed in the antibody distributions. There exist multiple peaks in each plot, which proves that inferring background antibody distributions solely from empty dorplets is inconclusive. This also explains the poorer AMI scores of demuxEM in simulation.*

C2.	While the tool itself seems useful, I do not see the utility of including the online experiment planning resource in the Results section of the current manuscript, especially considering that similar experiment planning tools already exist.

*Response to C2: We would like to first thank the reviewer for the suggestion. The major reason that we included the planner in the main body is to emphasize that it is based on a probabilistic model (a generative model, models droplet formation as a tangible random process), rather than a statistical model (fits curves to data), as with most other planners (Kang, et al., 2017; Stoeckius, et al., 2018). We believe that probabilistic generative models are more trustworthy and using them brings additional interpretability to the online planner than simply using pre-fit curves. However, we agree that the difference among the two types of models is subtle and including the experiment planner in the main text could be excessive, given the current length of the paper. We have moved the online planner to the Supplementary Materials (page 46) following the reviewer’s suggestion.*

C3.	The authors' claim that existing doublet detection methods which leverage gene expression information (e.g., DoubletFinder and Scrublet) cannot "reliably distinguish multiplets from transitional cells" runs counter to evidence described in the associated publications (e.g., DoubletFinder: McGinnis, Murrow & Gartner, 2019, Cell Systems; Scrublet: Wolock, Lopez & Klein, 2019, Cell Systems). Moreover, while it is true that DoubletFinder and Scrublet cannot identify doublets formed from transcriptionally-similar cells, other doublet detection algorithms have the potential to address this limitation (e.g., Wainer-Katsir & Linial, 2019, bioRxiv). The authors must either definitively prove these claims or remove them from the manuscript. More generally, it could be useful to explore how GMM-Demux can interface constructively with these existing methods (e.g., to improve SSM modeling and detection of SSMs, experiment planning, etc.), instead of just ignoring their existence.

*Response to C3: We thank the reviewer for the suggestion. We agree that the original claim could be misleading without supportive materials. We especially appreciate the idea from the reviewer that GMM-Demux could work in junction with RNA-based doublet finding methods. Following this lead, we have updated the Related Works section and we have applied both DoubletFinder (McGinnis, Murrow, & Gartner, 2019) and scrublet (Wolock, Lopez, & Klein, 2019) on the PBMC-1 dataset. We compared their doublet finding results against the MSM classification result of GMM-Demux, as well as the phony type GEM classification result based on the manual annotations of the surface marker data. The comparison result is provided below and added to the Result section (page 11):*

*As shown in the above figure, phony-type GEM clusters not only have high MSM percentages, they also have high percentages of doublets identified by DoubletFinder and scrublet. Detailed MSM and DoubletFinder-designated (DBF) doublet percentages of each GEM cluster are provided in the table below.*

*As shown in the table, the level of MSM and DBF-doublet percentages are in accordance in all clusters. However, it is evident that DoubletFinder underestimates the number of doublets in each cluster. This can also be observed in the tSNE plots, as the doublets in both the DoubletFinder and scrublet tSNE plots much scarcer than the phony-type GEM clusters.*

*Overall, this proves that GMM-Demux can be used together with RNA-based doublet finding methods to authenticate putative cell types, just as the reviewer anticipated.*

C4. Many of the authors' arguments rely on the notion that CD4/8 double-positive T-cells are too rare to be seen in this study. While it is very possible that these cells are indeed doublets, I think many would argue that they are not "non-existent" in PBMC samples and that this is not "conventional wisdom." I suggest that the authors utilize the gene expression data (e.g.. not just CITE-seq gating) to discern whether there are different clusters of CD4/8 double-positive T-cells that can be linked to singlet or doublet classifications (e.g., by looking at results from computational doublet detection techniques, total numbers of RNA and/or HTO UMIs, etc.). Leveraging the gene expression data would additionally improve the classification of 'mixed' GEM clusters (e.g., CD14+CD16+ non-classical monocytes), which the authors unsatisfactorily discuss due to their limited interpretation of CITE-seq gates.

*Response to C4: We thank the reviewer for pointing this out and we apologize for the confusion. In the manuscript, we stated that such cell types are rare in PBMC, as they should occur in very small populations. We did not deny the existence of such cell types. Following the reviewer’s suggestion, we verified the phony-type classifications with DoubletFinder (please refer to the previous response) and observe that the CD4+CD8+ T cell GEM cluster have 52.84% of its GEMs designated as doublets by DoubletFinder. This percentage is among the average doublet percentages of other phony-type GEMs, which validates the phony-type classification of the cluster by GMM-Demux.*

*We appreciate the suggestion by the reviewer that cell-type annotation could be further refined with RNA data and we think it is a very promising idea. However, to our disappointment, doublets rarely forms isolated clusters in the RNA space. Rather, they are likely to remain mixed with other pure-type GEM clusters. Below we present three examples: CD4+ T cells, CD8+ T cells and the double positive T cells (phony);*

*CD4+ T cells, monocytes and the CD4+ T-monocyte cells (phony); T cells, B cells and the T-B cells (phony).*

*As shown in all three plots, phony-type GEMs do not necessarily form separate clusters that are distant from the pure-type GEM clusters. This complicates our effort to refine the selection of pure-type GEMs by incorporating the RNA data. We attempted to further refine the selection of both CD16+ NK and CD16+ Monocyte GEMs by enforcing tight groups in both the RNA and the surface marker space. The refinement results of both cell types are presented in the figure below. Details of selection refinement are added to the Result section (page 11).*

*After refinement, the MSM percentage of both clusters reduced slightly, from 14.91% to 6.77% (CD16+ monocytes) and from 9.62% to 9.32% (CD16+ NK).*

*While the selection refinement of CD16+ monocyte is largely successful, the MSM percentage reduction of the CD16+ NK cluster is very moderate. This is perhaps due to both the large population of CD16+ NK GEM population as well as the inherit difficulties in separating biologically similar cell types, both in RNA space and in surface marker space. It is also worth noting that DoubletFinder failed to discover any doublet in the CD16+ NK GEM population as well (0% doublet rate).*

*While this does not disprove the idea of combining sample barcoding, surface marker data and RNA data to improve selections of pure-type GEMs, given that GMM-Demux is not a doublet identification method but a putative cell type verification method (our follow up work, CITE-sort (Lian, et al., 2020), to be appear on Bioinformatics, touches on the topic of doublet-aware cell type annotation), we decided to address the limitation of GEM annotations in our future work.*

Reviewer #2:

Summary: Xin et al present a tool (GMM-Demux) to accurately identify sample-barcoding-detectable multiplets and estimate the proportion of sample-barcoding-undetectable multiplets. The authors apply their method to real and simulated data sets. Additionally, the authors provide a freely available online experiment planner to help guide future experiments. While the tool seems to do what it is supposed to, I recommend that the authors demonstrate that their tool solves a significant problem and enables novel biological insight to motivate broad utility. Please find a more detailed list of concerns below:

*Response to summary: We thank the reviewer for the summary. We appreciate the reviewer’s suggestion of adding evidence proving the urgency of identifying doublets. To address this comment, we conducted additional scRNA-seq analysis for the PBMC-1 dataset and showed that phony type GEMs could confound downstream RNA analysis. Aside from removing doublets, GMM-Demux allows researchers to plan ahead with the online planner and drastically increase the throughput of scRNA-seq (reduces library prep cost) without worrying about doublet contaminations, which may lead to false positives as reported in many studies.*

Major:

C1. Methodologically the tool seems sound. However, in the current format it is difficult to judge how big of a problem GMM-Demux solves. Does the contamination of phony GEMs have any grave downstream effects? It is not clear why I need to care about them. I expect that emphasizing this point would increase utility of the tool.

For example, can the phony GEMs lead to erroneous discovery of novel cell types? The authors try to address this question in Section 5.5., where the authors illustrate the use of GMM-Demux by analyzing the PBMC-1 dataset. I find this exemplary analysis rather constructed. The idea of CITE-seq is to profile BOTH protein and RNA simultaneously. However, the authors use a protein-only gating strategy to derive cell types and ignore the RNA information completely. I wonder if the derived 17 GEM clusters would also appear when performing standard scRNAseq analysis on the RNA data. Do these phony GEM clusters (i.e. CD3+CD4+CD14+) actually produce separate clusters in the scRNAseq data? If not, then I am not sure those would be considered "novel candidate cell types" and the contamination seems to be rather subtle.Can the phony GEMs corrupt cell type expression profiles? The authors should evaluate if cell type marker profiles are impacted and compare marker gene lists before and after removing phony clusters.

*Response to C1: We thank the reviewer for the appreciation of our method and the suggestion of providing additional rationale for removing phony-type GEMs. Indeed, phony-type GEMs could confound downstream analysis. Below we show the RNA-based clustering result of the PBMC-1 dataset, with the MSM percentage of each cluster supplied in the parentheses (A). Comparing to the surface-marker-based annotation (B) of the dataset, the RNA-based clustering is less in accordance. Especially it fails to separate the NK GEM group from the CD8+ T cell group (Cluster 4). Additionally, 51.57% of the GEMs in cluster 6 are MSMs. Further decomposition of cluster 6 (E) shows that it consists of GEMs from multiple types, including 19.8% phony-type GEMs. After removing the phony-type GEMs, however, the RNA clustering result (C) is more in accordance with the surface-marker based annotations (D), improving the AMI score of clustering pure-type GEMs from 0.53 to 0.72. Further details are included in the revised Supplementary Materials (page 57-58).*

*While the perception of phony-type GEMs form isolated clusters is rational, we observe that in reality they are mostly embedded among pure-type GEM clusters (please refer to the subfigure B above and our response to reviewer 1 C4). However, this does not mean that they are not phony-type GEMs. RNA-based doublet identification methods, including DoubletFinder and scrubet, provide supporting evidence of their phony-type designations (please refer to our response to reviewer 1 C3).*

C2: It is not clear how big the improvement over existing methods is. I appreciate the fact the authors used a "Human Supervised" approach for comparison. On first sight the differences between the methods seem to be very small. What could be the downstream effects? The authors should also compare the heuristic method in their analysis in Section 5.5 or additional analyses as suggested.

Fig 6 - it is hard to evaluate stability by looking at a few (manually selected?) tSNE plots. The authors should perform a systematic analysis to evaluate stability. For example, show the variance of the heuristic classification across varying tl values and sub-sampling scenarios.

Fig 7 - The authors should expand the simulation analysis by testing multiple scenarios. Is the 69x improvement a stable result?

*Response to C2: This is a great suggestion and we thank the reviewer for asking it. We have conducted 9 additional simulations and benchmarked GMM-Demux against 3 other MSM classifiers including Seurat, MULTI-seq and demuxEM. Based on the benchmark results, GMM-Demux is the most stable and is highly accurate (please refer to our response to reviewer 1 C1). For each dataset, we repeated each method 20 times and reported their benchmark results in bar plots with error bars.*

*We have also conducted additional stability test following the reviewer’s suggestion and added the result to the Supplementary Materials (page 46). The stability test result is shown below. We compute the percentages of GEMs that changes classifications between repetitive runs. As shown below, Seurat is less stable than the other three methods.*

C3: Fig 8 - Non-proportionate Venn diagrams are a very misleading visualization. The authors should compare the estimated and observed proportion using a different visualization (maybe scatter plot?). Can the authors statistically evaluate significance of the agreement?

*Response to C3: We share the concern of the reviewer. However, when plotting the GEM-sample count compositions, we must incorporate both the number of GEMs of a sample barcoding composition, as well has information about member samples of that configuration. While scatter plots reveal the correlation between observed and model derived GEM counts, they do not portrait the member sample compositions. Proportionate Venn diagrams, on the other hand, underrepresent MSM types with small populations and changes shape between repetitive runs.*

*We think that our current logical Venn diagram is still perhaps the best compromise to show both the GEM counts and the member sample configuration of each Venn diagram compartment. Nevertheless, we have produced alternative visualizations including both log-proportionate Venn diagrams as well as scatter plots. We used the eulerr R package (Larsson, 2018) to generate the proportionate Venn diagrams (Euler diagrams). For the Euler diagrams, we let the area of each compartment proportional to the log transformed value of the corresponding GEM count. Both plots are attached below. A major problem with the proportionate Venn diagram (Euler diagram) is that the shape of the diagram changes as input data changes, which makes it more difficult to compare their results. We are happy to use these plots instead of the current logical Venn diagram in the manuscript if the reviewer thinks they are more informative.*

*Observed GEM counts of PBMC-1	Model-derived GEM counts of PBMC-1*

*Observed GEM counts of T-Memory	Model-derived GEM counts of T-Memory*

*We used chi-square test to measure the statistical significance of the agreement between the model-derived and the observed GEM counts. The p-values for the PBMC-1 and the T-memory datasets are 0.99 and 0.45, respectively. This proves that our droplet formation model is accurate at describing the droplet formation process.*

C4: The authors should comment on runtime.

*Response to C4: We thank the reviewer for this suggestion. We have added the runtime benchmark to the Result section. The runtime benchmark result is also shown below. GMM-Demux is the fastest among all 4 MSM classifiers.*

Minor:

C5.	The manuscript would benefit from shortening. Fig 1 is essentially a remake of Fig 1A from Stoeckius et al and not necessary. Fig 4 is informative but all 4 plots look the same and could benefit from possible compression. Consistent font should be used across all equation p.10 "This idea can be further expended into artificially create..."

*Response to C5: We thank the reviewer for these suggestions. We have followed the reviewer’s advice and replace Fig 1 to the Supplementary Materials. We will fix all formatting issues in our final version according to the publisher requests. We have compressed Fig 4 by moving the scatter plots of PBMC-2 and T-memory datasets to the Supplementary Materials (page 46). For the sentence on page 10, we have changed it to “This idea can be further extended...”.*

C6.	The Github repo is exceptionally well documented but includes minor typos which could be fixed by another thorough readthrough.

*Response to C6: We thank the reviewer for supporting our software. We have further polished the readme of our Github repo.*

*Additional Comments: We are in the process of depositing both in-house datasets (PBMC-1 and T-Memory) to GEO. They are pending release upon the acceptance of the paper.*

*References*

*Abdelaal, T., Michielsen, L., Cats, D., Hoogduin, D., Mei, H., Reinders, M., & Mahfouz, A. (2019). A comparison of automatic cell identification methods for single-cell RNA sequencing data. Genome Biology, 20(1), 194-194. Retrieved from https://academic.microsoft.com/paper/2971653850*

*Gaublomme, J., Li, B., McCabe, C., Knecht, A., Yang, Y., Drokhlyansky, E., . . . Regev, A. (2019). Nuclei multiplexing with barcoded antibodies for single-nucleus genomics. Nature Communications, 10(1), 2907-2907. Retrieved from https://academic.microsoft.com/paper/2953584518*

*Haider, S., & Pal, R. (2013). Integrated Analysis of Transcriptomic and Proteomic Data. Current Genomics, 14(2), 91-110. Retrieved from https://academic.microsoft.com/paper/2032964150*

*Kang, H., Subramaniam, M., Targ, S., Nguyen, M., Maliskova, L., McCarthy, E., . . . Ye, C. (2017). Multiplexed droplet single-cell RNA-sequencing using natural genetic variation. Nature Biotechnology, 36(1), 89-94. Retrieved from https://academic.microsoft.com/paper/2774307122*

*Larsson, J. (2018). eulerr: Area-Proportional Euler Diagrams with Ellipses. Retrieved from*

*https://academic.microsoft.com/paper/2786600960*

*Lian, Q., Xin, H., Ma, J., Konnikova, L., Chen, W., Gu, J., & Chen, K. (2020). Artificial-Cell-Type Aware Cell Type Classification in CITE-seq. bioRxiv. Retrieved from https://academic.microsoft.com/paper/3003608605*

*McGinnis, C., Murrow, L., & Gartner, Z. (2019). DoubletFinder: Doublet Detection in Single-Cell RNA Sequencing Data Using Artificial Nearest Neighbors. Cell systems, 8(4), 329. Retrieved from https://academic.microsoft.com/paper/2953151710*

*McGinnis, C., Patterson, D., Winkler, J., Conrad, D., Hein, M., Srivastava, V., . . . Gartner, Z. (2019). MULTI-seq: sample multiplexing for single-cell RNA sequencing using lipid-tagged indices. Nature Methods, 16(7), 619-626. Retrieved from https://academic.microsoft.com/paper/2951068818*

*Stoeckius, M., Zheng, S., Houck-Loomis, B., Hao, S., Yeung, B., Mauck, W., . . . Satija, R. (2018). Cell Hashing with barcoded antibodies enables multiplexing and doublet detection for single cell genomics. Genome Biology, 19(1), 224. Retrieved from https://academic.microsoft.com/paper/2785085486*

*Vinh, N., Epps, J., & Bailey, J. (2010). Information Theoretic Measures for Clusterings Comparison: Variants, Properties, Normalization and Correction for Chance. Journal of Machine Learning Research, 11, 2837-2854. Retrieved from https://academic.microsoft.com/paper/2162833336*

*Wolock, S., Lopez, R., & Klein, A. (2019). Scrublet: Computational Identification of Cell Doublets in Single-Cell Transcriptomic Data. Cell systems, 8(4). Retrieved from https://academic.microsoft.com/paper/2817838603*

**Second round of review**

**Reviewer 1**

GMM-Demux Revision Review

Summary

GMM-Demux is a computational pipeline which serves three purposes which the authors expand upon in the revised manuscript.

First, GMM-Demux classifies cells into sample barcode groups using a model-based Bayesian framework which operates on HTO count matrices. The authors have included excellently-executed new analyses in the revised manuscript (e.g., AMI, run-time, and stability tests on simulated data) which convincingly show that GMM-Demux outperforms alternative classifiers such as Seurat, MULTI-seq, and demuxEM. However, the authors miss the mark when outlining the differences in assumptions and limitations between GMM-Demux and these alternative workflows (see Major Comment #1).

Second, GMM-Demux estimates the SSM, MSM, and singlet rates for different sample-multiplexed scRNA-seq experimental designs. I appreciate that the authors moved these analyses to SI for the sake of brevity. However, upon a second reading of the manuscript, I noticed that the number of doublets estimated by GMM-Demux varies dramatically from the experimentally-validated doublet formation rate estimates provided in the 10x Genomics scRNA-seq User Guide (see Major Comment #2). While the validation of the MSM/SSM model is somewhat convincing, the authors fail to explain why their doublet estimates are higher than the doublet formation rate estimates generated from species-mixing experiments. I’m in no position to assert that the authors are necessarily wrong in this case – however, since the GMM-Demux doublet rate predictions are internally-consistent but not experimentally-validated, while the 10x doublet rate predictions are internally-consistent and experimentally-validated, the onus is on the authors to explain why 10x got it wrong. Alternatively, in the case that I am misinterpreting the author’s claims, the authors must clarify the writing substantially in order to avoid confusing the reader.

Third, GMM-Demux maps classification results onto RNA-seq and CITE-seq unsupervised clustering results and presents a statistical framework for determining whether clusters represent singlets (e.g., pure-type GEMs) or doublets (e.g., phony-type GEMs). We appreciate that the authors took our suggestion to leverage the scRNA-seq data for benchmarking GMM-Demux against existing computational doublet detection techniques. However, these analyses are not appropriately documented and seemed to conclude that GMM-Demux phony-type GEM classifications were more-accurate than the predictions from DoubletFinder and Scrublet (see Major Comments #3 and #4), even though the author’s own analyses suggest that this conclusion is incorrect (e.g., MSM-enriched clusters are identified by DoubletFinder but not by GMM-Demux phony-type GEM prediction).

On balance, I am excited and convinced by the author’s claims that GMM-Demux represents an improvement over alternative sample classification algorithms, and am comfortable with the formal publication of these analyses (upon inclusion of the suggested edits). Moreover, I am, in theory, excited about the statistical framework for leveraging sample classification results for authenticating cell type annotations. However, I have serious concerns about the multiplet formation model (e.g., why is predicted doublet rate so much higher than experimentally-validated doublet rates?) and the interpretations of the benchmarking analysis between phony-type GEM and doublet detection method predictions.

Comments on Author Responses

1. “[W]e initially planned to include the transcriptomics analysis in the manuscript at the time of writing. However, deterred by the inconsistent and sometimes very low correlations between mRNA and protein expressions (Haider & Pal, 2013) and that surface protein markers are often considered more reliable and are widely used to annotate the ground truth in cell type classification (Abdelaal, et al., 2019), we decided to drop the transcriptomics analysis and proceed with just the surface marker data for the purpose of sample demultiplexing and multiplet detection.”

While I appreciate that the authors trust protein expression patterns over transcriptomic signals in the context of cell type annotation, it is worth noting that GMM-Demux is presented as a tool for analyzing sample-multiplexed scRNA-seq data, of which only a subset will have associated surface protein expression data. Demonstrating that GMM-Demux will work on datasets without CITE-seq information is critical.

2. “We thank the reviewer for suggesting these compelling tools. At the time of finishing our first draft, neither DemuxEM (Gaublomme, et al., 2019) nor MULTI-seq (McGinnis, et al., 2019) was published.”

For what it’s worth, DemuxEM and MULTI-seq were first deposited onto bioRxiv in the fall/winter of 2018, and were published 6 months prior to your manuscript submission in Nature Communications and Nature Methods, respectively. Regardless, we find the authors new analytical comparisons between methods on simulated data very well-executed and convincing.

3. “We have moved the online planner to the Supplementary Materials (page 46) following the reviewer’s suggestion.”

Thanks! I also appreciate how the authors clarified the distinction between the experiment planning models.

4. “…phony-type GEM clusters not only have high MSM percentages, they also have high percentages of doublets identified by DoubletFinder and Scrublet… Overall, this proves that GMM-Demux can be used together with RNA-based doublet finding methods to authenticate putative cell types, just as the reviewer anticipated.”

This analysis is problematic for reasons discussed in Major Comment #3. Moreover, I personally feel like the distinction between a ‘cell type authentication’ and ‘doublet prediction’ tool is quite subtle – i.e., How is ‘authenticating’ a cell type different from determining it is not a doublet? Is predicting which cells are not doublets really that different from predicting which cells are doubles? The authors should make this distinction more clearly before publication.

5. “We appreciate the suggestion by the reviewer that cell-type annotation could be further refined with RNA data and we think it is a very promising idea. However, to our disappointment, doublets rarely forms isolated clusters in the RNA space.”

The statement runs counter to numerous observations in the literature, and the demonstrated utility of computational doublet detection algorithms – which the authors themselves utilize in this manuscript – requires doublets and singlets to cluster separately.

Major Comments

1. In the ‘Related Works’ section, the authors do a good job of explaining alternative sample classification workflows. However, they mischaracterize the assumptions and limitations of the MULTI-seq classification algorithm in two key ways.

First, the authors state that “[MULTI-seq] assumes that the HTO antibody solutions across all samples have similar concentrations and GEMs across all samples have HTO counts at similar magnitudes… it heavily relies on the assumption that all samples have similar HTO count profiles…” This is incorrect.

The MULTI-seq classification workflow involves iteratively (i) Modeling HTO count distributions using gaussian kernel density estimation, (ii) Identifying local maxima in each distribution, (iii) Defining on- and off-target barcode peaks as the maximum- and mode-associated local maxima, respectively, and (iv) Identifying the inter-maxima quantile threshold which produces the most singlets. Thresholding based on quantiles instead of absolute numbers of HTO counts helps avoid classification biases if some samples have higher HTO counts than others (e.g., due to different labeling concentrations, membrane association kinetics for different cell types, etc.), which would cause the on- and off-target local maxima to be in different relative positions. Moreover, while it is true that MULTI-seq assumes that “all samples have similar HTO count profiles,” this does not mean it assumes the local maxima are in the same positions, as the authors suggest. Instead, MULTI-seq assumes that the sample HTO distributions are generally bimodal, much as GMM-Demux does. With that being said, it is a fair criticism of MULTI-seq that the quantile threshold used is monolithic and may not be ideal for every barcode (although not for the reasons the authors suggest). For this reason, the GMM-Demux confidence threshold strategy is a good addition for the method and should be highlighted by the authors.

Second, the authors state that “When the profiles of sample-specific HTO antibody solutions diverge, the accuracy of MULTI-seq reduces and could even lead to execution failures (as shown in our simulation) as it fails to derive a cross-sample universal HTO count cutoff.” While it is true that MULTI-seq accuracy is limited when HTO distributions diverge (as is eloquently shown in the data simulations), this is unlikely to be the reason why MULTI-seq threw an error when applied to the 2-sample simulated data. As stated above, MULTI-seq identifies on- and off-target peaks for each HTO count distribution by finding the maximum- and mode-associated local maxima. This necessarily assumes that these local maxima are different – that is, that there are more off-target than on-target cells for every HTO distribution – which is an assumption that breaks down when only 2 samples are multiplexed, or when one multiplexed sample is far in excess of all other samples. The authors need to clarify this section dramatically.

2. Targeting 5,000 cells recovered from each of 4 samples suggests that cells were loaded into the droplet microfluidics device to target 20,000 total recovered cells. According to the 10x V2 user guide, this would result in a 15.4% doublet formation rate, while the authors note an expected 23.9% doublet rate. If this is a typo, please fix and update all analyses that rely on this number. If the true doublet rate is 23.9% and not 15.4%, please explain why this is the case, as the doublet rate estimates from 10x are experimentally validated. Similarly, the anticipated doublet rate from the 10x V2 user guide when 3,200 cells are loaded is 1.47% not 1.9%.

3. The comparative analyses of GMM-Demux to the computational doublet detection methods DoubletFinder and Scrublet are problematic for two reasons. First, the authors claim that DoubletFinder and Scrublet “only discover fractions of phony-type GEMs.” Looking at the embeddings in Fig. 11, it is perplexing how the authors could have come to this conclusion, as all of the phony-type GEM clusters are detected by both DoubletFinder and Scrublet. In fact, the only difference between the phony-type GEM and DoubletFinder results are the regions highlighted with red circles below, which are enriched with MSMs, suggesting that they are true phony-type GEMs that are being missed. This point must be addressed before publication.

Second, the authors claim that DoubletFinder underestimates the number of doublets in phony-type GEM clusters. However, the number of DoubletFinder predictions is a user-defined parameter, so erroneously setting this parameter value too low could explain this observation. The details of how the authors applied DoubletFinder and Scrublet to their data is also missing from the manuscript (were the methods applied to the RNA or CITE-seq counts? What parameters were selected?), making interpretation of this claim impossible.

4. In the ‘Related Works’ section, the authors do a good job of explaining the experimental strategies and algorithms which have been utilized previously for doublet detection. However, in this section the authors claim that the benchmarking strategies employed for testing the accuracy of doublet detection algorithms (e.g., cross-species, cross-donor, and cross-cell-type validation) have “limited applicabilities in practice” because they will not work well on scRNA-seq datasets describing transcriptionally-homogeneous cell types from the same donor. Notably, the authors ignore that many doublet detection algorithms have additionally been benchmarked against sample-multiplexing data. Moreover, while doublet detection algorithms certainly will not work well when applied to transcriptionally-homogenous scRNA-seq data, it is unclear if GMM-Demux will fare any better, as the method itself requires MSM enrichment in gene expression clusters which are separate from bona fide singlets. It is possible that GMM-Demux is able to identify doublets that are missed by DoubletFinder and Scrublet, but the authors do not prove this claim satisfactorily.

5. As a Methods paper, the authors are limited to 4-6 main text figures. While it is naturally up to the authors how to adhere to this limitation, I suggest the following figure progression (moving other figures to SI, particularly figures referenced only in the Methods section):

I. Figure 1: Introduction of SSM/MSM/singlet and phony-type/pure-type GEM nomenclature. Can either merge Fig. 1 with Fig. 2 or just move Fig. 1 to SI.

II. Figure 2: PBMC-1 classification results. Keep existing Fig. 3.

III. Figure 3: Classification method performance comparison. Merge Fig. 6, Fig. 7, and Fig. S5 to make points about classification accuracy, run time, and classification result stability. Should move data simulation embeddings (including all of the simulations, not just the 4-sample simulation) to SI.

IV. Figure 4: SSM estimation verification. Keep existing Fig. 8, and perhaps incorporate Fig. S15.

V. Figure 5: Benchmarking phony-type GEM classifications. Visualize low-input scRNA-seq phony-type GEM verification experiment (which was a very smart experiment, by the way), and comparisons of GMM-Demux MSM and phony-type GEM classifications with DoubletFinder/Scrublet predictions.

6. While the comparisons between GMM-Demux and Seurat are well-executed, the authors should de-emphasize this part of their manuscript in favor of the comparisons to MULTI-seq and demuxEM. The poor performance of the Seurat classifier has already been described in numerous publications – hence the development of Solo, demuxEM, and the MULTI-seq classification pipelines. It is fine to make these points yet again in SI, but the key comparisons to focus on are between GMM-Demux and demuxEM/MULTI-seq.

7. I agree with Reviewer #2 that the manuscript, even in its now revised form, would benefit from significant shortening. For example, using the main text to explain that “each dot in the tSNE plot represents a GEM,” that, “the color of the GEM specifies the class of the GEM,” that, ”GEMs with similar HTO UMI count profiles are more likely to be drawn close to each other,” and finally that, “GEMs with similar HTO UMI count profiles are likely to be grouped together but there is no guarantee” is excessive — especially considering the likely readership for this manuscript. This point also holds true for the subsequent paragraph where the authors note that “GEMs with higher HTO UMI counts of the sample have darker colors,” as well as numerous other points throughout the manuscript. While I appreciate the author’s thoroughness, the deluge of details detracts from the main conclusions of the work, and can be much more simply and effectively explained in the figure legends.

Minor Comments

1. Pg. 6, Line 39: “Among all three classifiers, GMM-Demux and MULTI-seq adhere to the HTO heat maps the most.” This is a vague statement, either remove or clarify (perhaps with summary statistics).

2. Pg. 4, Line 55: Cells were cultured at 1 x 106 cells/mL, not 1 x 10-6 cells/mL.

3. “Online Cell Hashing Experiment Planner” should be renamed “Online Sample Multiplexing Experiment Planner” to encompass other sample multiplexing methods beyond cell hashing.

4. The authors may consider using more descriptive Results sub-section headers which convey the main conclusion(s) of the described analyses. For example, instead of “Joint analysis with surface marker data,” the authors could title the sub-section, “GMM-Demux classifications distinguish phony- and pure-type GEM clusters.”

5. The authors wrote that, “we refine the selection of both CD16+ GEM groups by clipping out GEMs that disperse into the CD16- GEM cluster.” What do they mean by “clipping out”? The authors should use unsupervised clustering on the scRNA-seq data to perform this “refinement.”

6. Very generally, this manuscript would benefit greatly from having the tightened and typos fixed.

7. Pg. 11, Line 46: “We show that phony-type GEMs can confound downstream analysis and degrade RNA clustering accuracy, as well as generating low-quality clusters with high MSM concentrations.” The RNA clustering accuracy point is well taken. However, saying that leaving phony-type GEMs in scRNA-seq data results in “low-quality clusters with high MSM concentrations,” to my interpretation, is self-evident. Put another way, why is it surprising that leaving phony-type GEMs in your data – which are known to be clusters with high MSM concentrations – results in the presence of clusters with high MSM concentrations?

8. Pg. 22, Line 35: “The heuristic classifier currently is the only analytical tool for processing sample barcoding data.” This is untrue, and is an especially confusing statement since the authors state that “there are three analytical methods for processing sample barcoding” in the following sentence. Please remove.

9. Color legend is missing from Fig. S4, Fig. 4.

10. Pg. 3, Line 17: “Existence of such PBMC types defies conventional wisdom, where CD3+ CD19+ cells and CD4+ CD8+ T cells are believed to be extremely rare.” Since CD4+ CD8+ T-cells are known to exist, the authors should consider removing the term “defies conventional wisdom.” Perhaps switch to, “Existence of such PBMC types at the observed frequencies is unlikely, as CD3+ CD19+ cells and CD4+ CD8+ T cells are believed to be extremely rare.”

11. Pg. 3, Line 44: “Most importantly, they cannot determine whether an alleged novel-cell-type-defining GEM cluster consists of mainly pure-type GEMs. Hence they cannot use MSM classification results to verify the legitimacy of putative novel cell types in scRNA-seq.” This statement is a bit misleading, as the existing sample classification algorithms do not aim to use the classification results to test for cluster legitimacy. This is like saying that unsupervised clustering does not identify differentially-expressed genes between clusters – clustering is a necessary first-step before DGE analysis, but the main goal is not DGE analysis.

**Reviewer 2**

I commend the authors for their efforts to address my concerns.

C1, however, remains partially unsolved. As the authors point out, phony GEMs rarely form isolated clusters. The question remains how large of an impact these phony-type GEMs have on the inferred expression profiles of given clusters. The authors should compare expression profiles derived from clusters before/after removing phony-type GEMs. Does the inclusion of phony-type GEMs lead to ‘wrong’ marker gene lists? How is differential expression analysis impacted?

**Authors Response**

**Point-by-point responses to the reviewers’ comments:**

Reviewer #1:

Summary: GMM-Demux is a computational pipeline which serves three purposes which

the authors expand upon in the revised manuscript.

First, GMM-Demux classifies cells into sample barcode groups using a model-based Bayesian framework which operates on HTO count matrices. The authors have included excellently-executed new analyses in the revised manuscript (e.g., AMI, run-time, and stability tests on simulated data) which convincingly show that GMM-Demux outperforms alternative classifiers such as Seurat, MULTI-seq, and demuxEM. However, the authors miss the mark when outlining the differences in assumptions and limitations between GMM-Demux and these alternative workflows (see Major Comment #1).

Second, GMM-Demux estimates the SSM, MSM, and singlet rates for different sample-multiplexed scRNA-seq experimental designs. I appreciate that the authors moved these analyses to SI for the sake of brevity. However, upon a second reading of the manuscript, I noticed that the number of doublets estimated by GMM-Demux varies dramatically from the experimentally-validated doublet formation rate estimates provided in the 10x Genomics scRNA-seq User Guide (see Major Comment #2). While the validation of the MSM/SSM model is somewhat convincing, the authors fail to explain why their doublet estimates are higher than the doublet formation rate estimates generated from species-mixing experiments. I’m in no position to assert that the authors are necessarily wrong in this case – however, since the GMM-Demux doublet rate predictions are internally-consistent but not experimentally-validated, while the 10x doublet rate predictions are internally-consistent and experimentally-validated, the onus is on the authors to explain why 10x got it wrong. Alternatively, in the case that I am misinterpreting the author’s claims, the authors must clarify the writing substantially in order to avoid confusing the reader.

Third, GMM-Demux maps classification results onto RNA-seq and CITE-seq unsupervised clustering results and presents a statistical framework for determining whether clusters represent singlets (e.g., pure-type GEMs) or doublets (e.g., phony-type GEMs). We appreciate that the authors took our suggestion to leverage the scRNA-seq data for benchmarking GMM-Demux against existing computational doublet detection techniques. However, these analyses are not appropriately documented and seemed to conclude that GMM-Demux phony-type GEM classifications were more-accurate than the predictions from DoubletFinder and Scrublet (see Major Comments #3 and #4), even though the author’s own analyses suggest that this conclusion is incorrect (e.g., MSM-enriched clusters are identified by DoubletFinder but not by GMM-Demux phony-type GEM prediction).

On balance, I am excited and convinced by the author’s claims that GMM-Demux represents an improvement over alternative sample classification algorithms, and am comfortable with the formal publication of these analyses (upon inclusion of the suggested edits). Moreover, I am, in theory, excited about the statistical framework for leveraging sample classification results for authenticating cell type annotations. However, I have serious concerns about the multiplet formation model (e.g., why is predicted doublet rate so much higher than experimentally-validated doublet rates?) and the interpretations of the benchmarking analysis between phony-type GEM and doublet detection method predictions.

Comments on Author Responses

1. “[W]e initially planned to include the transcriptomics analysis in the manuscript at the time of writing. However, deterred by the inconsistent and sometimes very low correlations between mRNA and protein expressions (Haider & Pal, 2013) and that surface protein markers are often considered more reliable and are widely used to annotate the ground truth in cell type classification (Abdelaal, et al., 2019), we decided to drop the transcriptomics analysis and proceed with just the surface marker data for the purpose of sample demultiplexing and multiplet detection.”

While I appreciate that the authors trust protein expression patterns over transcriptomic signals in the context of cell type annotation, it is worth noting that GMM-Demux is presented as a tool for analyzing sample-multiplexed scRNA-seq data, of which only a subset will have associated surface protein expression data. Demonstrating that GMM-Demux will work on datasets without CITE-seq information is critical.

2.	“We thank the reviewer for suggesting these compelling tools. At the time of finishing our first draft, neither DemuxEM (Gaublomme, et al., 2019) nor MULTI-seq (McGinnis, et al., 2019) was published.”

For what it’s worth, DemuxEM and MULTI-seq were first deposited onto bioRxiv in the fall/winter of 2018, and were published 6 months prior to your manuscript submission in Nature Communications and Nature Methods, respectively. Regardless, we find the authors new analytical comparisons between methods on simulated data very well-executed and convincing.

3.	“We have moved the online planner to the Supplementary Materials (page 46) following the reviewer’s suggestion.”

Thanks! I also appreciate how the authors clarified the distinction between the experiment planning models.

4.	“...phony-type GEM clusters not only have high MSM percentages, they also have high percentages of doublets identified by DoubletFinder and Scrublet... Overall, this proves that GMM-Demux can be used together with RNA-based doublet finding methods to authenticate putative cell types, just as the reviewer anticipated.”

This analysis is problematic for reasons discussed in Major Comment #3. Moreover, I personally feel like the distinction between a ‘cell type authentication’ and ‘doublet prediction’ tool is quite subtle – i.e., How is ‘authenticating’ a cell type different from determining it is not a doublet? Is predicting which cells are not doublets really that different from predicting which cells are doubles? The authors should make this distinction more clearly before publication.

5.	“We appreciate the suggestion by the reviewer that cell-type annotation could be further refined with RNA data and we think it is a very promising idea. However, to our disappointment, doublets rarely forms isolated clusters in the RNA space.”

The statement runs counter to numerous observations in the literature, and the demonstrated utility of computational doublet detection algorithms – which the authors themselves utilize in this manuscript – requires doublets and singlets to cluster separately.

*Response to summary: We greatly thank the reviewer for the great amount of effort he/she put in. We believe the quality of our work has been greatly improved as a result and we greatly appreciate the comments. Before proceeding to the point-by-point response, we want to further clarify the differences between phony-type GEM identification and cell type authentication.*

*The difference between the two is indeed subtle but important in our opinion. We tried to be as careful as we can to not overclaim the functionality and the contribution of GMM-Demux. Phony-type GEM identification (also known as cross-cell-type multiplet detection) tools attempt to find droplets whose expression profiles (RNA or surface marker profiles) show moderate affinity to multiple distinctive cell types; whereas cell type authentication tries to determine whether a pre¬selected GEM cluster (a putative) represents a genuine cell type or is merely a product of phony-type multiplets.*

*GMM-Demux, as a cell type authentication tool, does not identify phony-type GEMs by itself. It can be used in junction with high-fidelity clustering methods to identify phony-type GEMs but it is not designed to do this alone. Ideally, assuming that there exists a clustering algorithm that not only accurately and reliably separates GEMs of different cell types into distinct clusters, but also isolates phony-type GEMs into separate clusters as well, then one can apply GMM-Demux to the clustering output and authenticate each cluster. The “Joint Analysis with Surface Marker Data” section provides an illustration of this process, except that the “perfect clustering” is performed through manual gating in the surface marker space.*

*As perhaps known by the reviewer, clustering in scRNA-seq is not a satisfactorily resolved problem and is an entire research topic of its own. In a sense, GMM-Demux is orthogonal to the clustering input: given a good clustering input, GMM-Demux will be able to accurately identify phony-type clusters; given a bad clustering input, GMM-Demux would classify many clusters as mixture-type clusters. This reliance on good clustering algorithms is not unique to GMM-Demux, and GMM-Demux is not the only method that requires phony-type GEMs to form distinct clusters in order to generate high fidelity phony-type GEM identification. Both scrublet and DoubletFinder require descent clustering performance to work well, as mentioned in their manuscripts, quote “since DoubletFinder requires putative doublets to cluster separately from singlets in PC space, we did not expect DoubletFinder to robustly detect homotypic doublets” (DoubletFinder[2]) and “varying the number and size of simulated cell clusters, the doublet detector accurately identified up to 99% of doublets that were generated between cells from different clusters (neotypic doublets) with 99% precision, but only if clusters were sufficiently well separated” (Scrublet[3]). By decoupling cell type authentication from clustering and limiting the functionality of GMM-Demux to cell type authentication alone, end users are free to choose their favorite clustering framework which is most suitable to their specific needs to use in junction with GMM-Demux.*

*This also leads to why we perform manual gating as opposed to simply performing clustering based on RNA data. Surface markers are more reliable in cell type calling[4]. While it is possible that after further fine tuning, phony-type GEM clusters could be separated from pure-type GEM clusters, we believe that this is unnecessary for our paper and would be misleading. In our previous manuscript revision, we faithfully reported the analytical results generated by scanpy[5]. In fact, the UMAP plots provided in the previous response are already products with moderate manual intervention: we first performed differential analysis among individual cell types (after manual gating in the surface marker space) to detect the marker genes of each cell type and then performed UMAP transformation only using these differentially expressed genes as features. Otherwise, if we simply follow the default procedure of scanpy (assuming we are agnostic to the cell types and the feature genes in the dataset) and just select the top varying genes, the UMAP plot would look like the following:*

*As shown in the above figure, without manual intervention, both pure-type and phony-type GEM clusters are less separated from each other than in the plots in Figure S16. Furthermore, identifying phony-type GEM clusters through clustering is especially challenging since the number of clusters could be massive ( biological cell types could theoretically create 2 − − 1 phony cell types) and there could be significant population imbalances between clusters (phony-type GEM clusters are very small, compared to pure-type GEM clusters). All of these issues create significant challenges for the identification of phony-type GEM clusters [6], [7] and underscore the importance of the clustering optimizations included in DoubletFinder and scrublet. We do not want to mislead the readers with regards to the challenges in distinguishing phony-type GEM clusters from pure-type GEM clusters in the transcriptomic space.*

*Additionally, it has been observed in previous studies that multiplets often do not form distinct clusters. For instance, in scrublet, the authors found that doublets form distinct clusters only after careful tuning of parameters. Below we supply the plot taken from the srublet manuscript. Given that the focus of GMM-Demux is not multiplet-aware clustering in the transcriptomic space, we leave the investigation of why phony-type GEMs do not readily isolate themselves into separate clusters for our future work.*

*However, we do recognize and appreciate the concern raised by the reviewer. It is a valid point that we should emphasize that GMM-Demux does not identify phony-type GEMs on its own (unlike DoubletFinder and scrublet). We have addressed this by adding the following to the Discussion section:*

Major Comments

Comment (C)1. In the ‘Related Works’ section, the authors do a good job of explaining alternative sample classification workflows. However, they mischaracterize the assumptions and limitations of the MULTI-seq classification algorithm in two key ways.

First, the authors state that “[MULTI-seq] assumes that the HTO antibody solutions across all samples have similar concentrations and GEMs across all samples have HTO counts at similar magnitudes... it heavily relies on the assumption that all samples have similar HTO count profiles...” This is incorrect.

The MULTI-seq classification workflow involves iteratively (i) Modeling HTO count distributions using gaussian kernel density estimation, (ii) Identifying local maxima in each distribution, (iii) Defining on- and off-target barcode peaks as the maximum- and mode-associated local maxima, respectively, and (iv) Identifying the inter-maxima quantile threshold which produces the most singlets. Thresholding based on quantiles instead of absolute numbers of HTO counts helps avoid classification biases if some samples have higher HTO counts than others (e.g., due to different labeling concentrations, membrane association kinetics for different cell types, etc.), which would cause the on- and off-target local maxima to be in different relative positions. Moreover, while it is true that MULTI-seq assumes that “all samples have similar HTO count profiles,” this does not mean it assumes the local maxima are in the same positions, as the authors suggest. Instead, MULTI-seq assumes that the sample HTO distributions are generally bimodal, much as GMM-Demux does. With that being said, it is a fair criticism of MULTI-seq that the quantile threshold used is monolithic and may not be ideal for every barcode (although not for the reasons the authors suggest). For this reason, the GMM-Demux confidence threshold strategy is a good addition for the method and should be highlighted by the authors.

Second, the authors state that “When the profiles of sample-specific HTO antibody solutions diverge, the accuracy of MULTI-seq reduces and could even lead to execution failures (as shown in our simulation) as it fails to derive a cross-sample universal HTO count cutoff.” While it is true that MULTI-seq accuracy is limited when HTO distributions diverge (as is eloquently shown in the data simulations), this is unlikely to be the reason why MULTI-seq threw an error when applied to the 2-sample simulated data. As stated above, MULTI-seq identifies on- and off-target peaks for each HTO count distribution by finding the maximum- and mode-associated local maxima. This necessarily assumes that these local maxima are different – that is, that there are more off-target than on-target cells for every HTO distribution – which is an assumption that breaks down when only 2 samples are multiplexed, or when one multiplexed sample is far in excess of all other samples. The authors need to clarify this section dramatically.

*Response to C1: We thank the reviewer for the correction and providing very detailed information about the implementation of MULTI-seq. We apologize for inaccurately characterizing MULTI-seq in the manuscript. What we meant is that MULTI-seq adjust the quantile thresholds between the on- and off- targets in all sample barcodes in a lock-step fashion[8]. We hypothesized that for this to work as intended, the shape of antibody distributions in all barcodes have to be similar. For example, if two hypothetical barcodes have the following distributions, then we believe that the classification accuracy of MULTI-seq would be reduced, as the two barcodes have different optimal quantile thresholds (highlighted in red circles). However, we do not think this is a huge limitation of MULTI-seq as we do not observe drastic variations in the shape of antibody distributions between sample barcodes.*

*That leaves us really puzzled as to why MULTI-seq does not work as intended when we run it with two samples. By the design of MULTI-seq, this should not have happened. Specifically, MULTI-seq should perform the 4 steps in each iteration as the reviewer suggested, and then remove the negative droplets before the next iteration. It keeps iterating until after an iteration, there are no negative droplets left. What we observe is that when we feed it with two samples, MULTI-seq keeps iterating until there is no droplet left. All droplets are classified as negative droplets and it stops and throws an error that it cannot rescue any droplet because all droplets are negative droplets. It appeared to us that for some reason MULTI-seq cannot converge to a stable quantile threshold for this benchmark which led to our original conclusion.*

*Thanks to the reviewer, now it is clear that the problem is due to a violation of a model assumption. Specifically, the following code in MULTI-seq:*

*As the reviewer suggested, the intention of these two lines (49, 50) is to obtain the on- and off-target maxima, as illustrated by the two red vertical lines shown in the plot below (taken from the MULTI-seq github page (https://github.com/chris-mcginnis-ucsf/MULTI-seq)):*

*However, the above code does not reliably carry out this task. Line 49 finds the x coordinate that generates the largest frequency (largest y), while line 50 finds the local maxima with the largest x coordinate. So instead of finding the on- and off-target peaks, the above code finds the highest peak and the right-most local peak (local maxima), respectively. As the reviewer suggested, if the shape of the distribution is horizontally turned over (as shown in the figure below), then the two maxima might not represent the on- and off-target Gaussian components and both can fall in to the on-target Gaussian component.*

*Following this discovery (and many thanks to the reviewer), we have updated the explanation of MULTI-seq as follows:*

C2. Targeting 5,000 cells recovered from each of 4 samples suggests that cells were loaded into the droplet microfluidics device to target 20,000 total recovered cells. According to the 10x V2 user guide, this would result in a 15.4% doublet formation rate, while the authors note an expected 23.9% doublet rate. If this is a typo, please fix and update all analyses that rely on this number. If the true doublet rate is 23.9% and not

15.4%, please explain why this is the case, as the doublet rate estimates from 10x are experimentally validated. Similarly, the anticipated doublet rate from the 10x V2 user guide when 3,200 cells are loaded is 1.47% not 1.9%.

*Response to C2: We appreciate the keen observation made by the reviewer. This is also a delicate but important finding we discovered in this work and party why we initially incorporated the singlet/MSM rate profiling results in the main text. We observe that the doublet rates included in the 10X user guide seem to be significantly under-estimated. However, due to the delicate nature of dealing with commercial content, we opt to not directly compare against the 10X user guide.*

*10X did not reveal details of their experimental validation process. Based on the information provided by Grace X.Y. Zheng et al., likely this is obtained through cross-species validation (specifically, through human-mouse validation). From the perspective of sample barcoding, a human-mouse 2-species cross-species scRNA-seq experiment is analogous to a 2-sample sample barcoding experiment.*

*We tried to reproduce the aforementioned 2-sample sample barcoding experiment (recovering 20,000 cells) using our experimental planner. With a capture rate of 55% and a cell-solution-dropet count of 80,000, based on our droplet formation model, it requires 48490 cells to recover 20,000 droplets. Among all recovered droplets, 15.04% of them are estimated to be MSMs and 12.22% are estimated as SSMs. In a human-mouse 2-species cross-species experiment, MSMs are analogous to human-mouse multiplets, while SSMs are analogous to human-human and mouse-mouse multiplets. If the definition of multiplet is simply MSM, then the deviation of multiplet rates between GMM-Demux estimation (15.04%) and 10X user guide (15.4%) is not significant.*

*It is very important to note that the above analysis is only based on our speculation of how 10X Genomics experimentally validated the multiplet rates. Detailed information of their multiplet profiling procedure is not made publicly available.*

*Nevertheless, the 10X user guide cannot explain why the MSM rates alone, excluding the SSMs, in our PBMC and T-Memory experiments exceed the 10X-user-guide reported multiplet rates (10X user guide suggested that the doublet rate of an experiment recovering 10k cells would be 7.6% but the observed MSM rate alone is 10.57%). Similarly, we found that approximately 25,000 cells would be required to recover fewer than 10,000 GEMs (including both singlets and multiplets). This is also way beyond the suggested 17,400 cells reported by the 10X user guide. This discrepancy is not isolated to our work. In MULTI-seq, the authors isolated 9,110 non-MSMs (MULTI-seq categorizes all non-MSM droplets as “singlets”) from 10,427 human and mouse cells (sample barcoding with 9 samples), leading to an MSM rate of 12.6%, which is also well beyond the 10X-user-guide reported 7.6% multiplet rate. In the Cell Hashing paper (Marlon Stoeckius et al.), Seurat identified 2974 MSMs out of 16,976 recovered cells, leading to an MSM rate of 17.5%, which is also beyond the suggested 15.4%. (This is a significant deviation. Recovery of ~17k cells has a larger MSM rate than the 10X-estimated total multiplet rate, including both MSMs and SSMs, of 20k cells).*

*To further validate our finding, we have conducted an additional 4-HTO endoscopic biopsy cell hashing experiment. In this experiment, we loaded a total of ~20600 cells and recovered 7113 cells with a 12.71% MSM percentage, which is again much higher than the 10X-user-guide suggested 5.4% total multiplet rate.*

*We also processed the above dataset with MULTI-seq, which reported an MSM rate of 10.7%. This also deviates from the 10X user guide and supports the MSM classification result of GMM-Demux.*

*Additionally, a recent bioRxiv paper also reported experimentally validated, higher multiplet rates than suggested in the 10X user guide[9]. Below are direct quotes from the paper: “We found that the doublet rate was 25% in the C1 96 platform, 33% in the C1 HT platform, 26% in the iCell8 platform, 28% in the Chromium V1 platform and 21% in the Chromium V2 platform. In general, the doublet rates we estimated here are higher than what was previously reported in the different platforms based on mixed species experiment with human and mouse cell lines” and “alternatively, it is possible that the mixed species cell experiment frequently employed to judge doublet rates underestimates the true double rate because mouse and human cell lines are not likely to re-aggregate in suspension”. We hope that this sheds light on why our observed and estimated multiplet rates are higher than the numbers from the 10X user guide.*

*Finally, we could not find information from 10X which states that the doublet formation rate associated with recovery of 20,000 cells is 15.4%. Below is a screenshot of the Reagent Kits*

*User	Guide	V2f	(https://support.10xgenomics.com/single-cell-gene-expression/library-*

*prep/doc/user-guide-chromium-single-cell-3-reagent-kits-user-guide-v2-chemistry). The document does not include doublet information beyond recovering 10,000 cells.*

*Overall, while we are also puzzled by the discrepancy between the observed MSM rates and the 10X-supplied multiplet rate estimations, we do not want to get entangled with comparisons against non-peer-reviewed commercial content. Hence, we opt to not directly comment on the 10X user guide.*

C3. The comparative analyses of GMM-Demux to the computational doublet detection methods DoubletFinder and Scrublet are problematic for two reasons. First, the authors claim that DoubletFinder and Scrublet “only discover fractions of phony-type GEMs.” Looking at the embeddings in Fig. 11, it is perplexing how the authors could have come to this conclusion, as all of the phony-type GEM clusters are detected by both DoubletFinder and Scrublet. In fact, the only difference between the phony-type GEM and DoubletFinder results are the regions highlighted with red circles below, which are enriched with MSMs, suggesting that they are true phony-type GEMs that are being missed. This point must be addressed before publication.

Second, the authors claim that DoubletFinder underestimates the number of doublets in phony-type GEM clusters. However, the number of DoubletFinder predictions is a user-defined parameter, so erroneously setting this parameter value too low could explain this observation. The details of how the authors applied DoubletFinder and Scrublet to their data is also missing from the manuscript (were the methods applied to the RNA or CITE-seq counts? What parameters were selected?), making interpretation of this claim impossible.

*Response to C3: We apologize for the confusion. We did not mean that DoubletFinder (DBF) only recovers a fraction of phony-type GEM clusters when we wrote “only discover fractions of phony-type GEMs”. We were referring to Table 8 (supplied below) where, in most phony type clusters, DoubletFinder only discovers ~50% (except for the CD3+CD4+CD14+CD56+ triple-type cluster, highlighted in blue shade) of phony-type GEMs. The general classification results between DoubletFinder, scrublet and GMM-Demux+manual gating are highly concordant. This not only proves the efficacy of DoubletFinder and scrubet, but also shows that the GMM-Demux classification is highly likely to be correct as well.*

*We want to reemphasize that Table 8 and Figure 10 (the original Figure 11 in the previous revision) do not prove that GMM-Demux “achieves a better phony-type identification performance”. They simply prove that given the surface-marker-facilitated, manually gated putative cell types, GMM-Demux is likely to derive the correct classification of each putative cell type, since all GMM-Demux classified phony cell types have high MSM and high DBF-assigned multiplet percentages, and GMM-Demux classified pure cell types have low MSM and low DBF-assigned multiplet percentages. It is not our intention to directly compare GMM-Demux against DoubletFinder and scrublet in terms of their phony-type GEM detection abilities, as GMM-Demux does not detect phony-type GEMs by itself.*

*It is not our intention to criticize the performance of DoubletFinder and scrublet, which work well in the transcriptomic space in many scRNA-seq datasets. In fact, we intend to use both methods to support GMM-Demux. To clarify, we have removed the statement “DoubletFinder and scrublet can only identify a fraction of the multiplets” and amended the paragraph as the following:*

*We ran both scrublet and DoubletFinder using their default parameters. We believe that the performance of both methods can be improved with parameter tuning, but this would defeat the purpose of a fair comparison, as it requires using the GMM-Demux classification result as a reference. For instance, DoubletFinder requires an estimation of the number of cross-cell-type multiplets in the dataset as an input. This requires the end user to have the following prior knowledge: 1) the number of cell types in the assay; 2) the population size of each cell type and 3) the total multiplet rate of the assay. Given a vector of frequencies of individual cell types, DoubletFinder infers the homotypic multiplet rate by calculating the sum of squares of frequencies of individual cell types. For instance, if there are three cell types with a frequency of [0.1, 0.3, 0.6], then DoubletFinder would assume that the homotypic multiplet percentage among all multiplets would be sum(0.01, 0.09, 0.36) = 0.46. The total count of cross-cell-type multiplets would then be Total_cell_count * doublet_rate * (1 – homotypic_proportion). (There is a typo in the DoubletFinder manuscript. It should be DDR = (1-pHomo) * TDR, not DDR = pHomo * TDR). When knowledge about cell types and their population composition in the assay is unavailable, DoubletFinder would then require the end user to use the clustering result instead, treating each cluster as a separate cell type. We argue that this is a rather crude procedure to infer the number of cross-cell-type multiplets. The homotypic multiplet rate is highly sensitive to the accuracy of cell type composition estimation. For instance, instead of clustering cells into 4 clusters with a frequency of [0.25, 0.25, 0.25, 0.25], if the clustering algorithm increases its resolution and clusters cells into 10 clusters with each cluster having a percentage of 0.1, then the homotypic frequencies would drop from 0.25 to 0.1 (a 60% decrease). Furthermore, DoubletFinder requires accurate knowledge about the total multiplet rate of the dataset, which is also not readily and reliably available (please refer to our response to C2). Below we present a panel of DoubletFinder classification results of the PBMC-1 dataset under different total multiplet rate settings, from 0.075 to 0.3. From an end user’s perspective, without sample-barcoding knowledge, it is difficult to judge which parameter provides the most accurate result. As our purpose is not to compare GMM-Demux to DoubletFinder, but rather to use DoubletFinder and scrublet to support the cell type authentication results of GMM-Demux, we think the current figure is sufficient. However, we are happy to yield the opportunity to the reviewer to help us set the most appropriate parameters upon further request.*

*Screenshots about our scrublet and DoubletFinder executions are provided below: Scrublet:*

***DoubletFinder****:*

*We used pN = 0.25 and pK = 0.01 as suggested in the original DoubletFinder paper. In our manuscript, we set the total multiplet rate to 0.125 to generate the DoubletFinder figure. We have added a section in the Supplementary Materials (S12) that details the parameter choices of DoubletFinder and scrublet*

*Overall, given that GMM-Demux and DoubletFinder serve different purposes and we are not attempting to directly compare their performances (rather, our intent is to use the RNA multiplet identification methods to reinforce GMM-Demux), we do not think that parameter fine-tuning for DoubletFinder and scrublet is required for our work but we are also happy to comply if sufficient rationale is provided.*

C4. In the ‘Related Works’ section, the authors do a good job of explaining the experimental strategies and algorithms which have been utilized previously for doublet detection. However, in this section the authors claim that the benchmarking strategies employed for testing the accuracy of doublet detection algorithms (e.g., cross-species, cross-donor, and cross-cell-type validation) have “limited applicabilities in practice” because they will not work well on scRNA-seq datasets describing transcriptionally-homogeneous cell types from the same donor. Notably, the authors ignore that many doublet detection algorithms have additionally been benchmarked against sample-multiplexing data. Moreover, while doublet detection algorithms certainly will not work well when applied to transcriptionally-homogenous scRNA-seq data, it is unclear if GMM-Demux will fare any better, as the method itself requires MSM enrichment in gene expression clusters which are separate from bona fide singlets. It is possible that GMM-Demux is able to identify doublets that are missed by DoubletFinder and Scrublet, but the authors do not prove this claim satisfactorily.

*Response to C4: We thank the reviewer for the encouraging comments about our explanation. We agree with the reviewer that from the aspect of multiplet detection, cross-sample (sample-barcoding), cross-donor, cross-cell-type and cross-species multiplet identification methods are conceptually similar. They all require some kind of unique identifier that is specific to a subpopulation of cells and they all identify doublets by finding droplets that contain multiple identifiers. With the cross-sample methods, it is the sample barcodes. With the cross-donor methods, it is the donor-specific SNPs. With the cross-cell-type methods, it is the cell type specific expression profiles and with the cross-species methods, it is the species-specific genes. Each method is orthogonal to other methods and neither can distinguish singlet from multiplets that are homotypic in their own respective aspects. The cross-sample method cannot distinguish singlets from the same-sample multiplets. The cross-donor method cannot distinguish singlets from the same-donor multiplets. The cross-cell-type method cannot distinguish singlets from the same-cell-type multiplets. The cross-species method cannot distinguish singlets from the same-species multiplets.*

*What is most special about GMM-Demux, as compared to demuxlet (cross-donor), MULTI-seq (cross-sample), DemuxEM (cross-sample), DoubletFinder (cross-cell-type) or scrublet (cross-cell-type) is that with the droplet formation model, GMM-Demux is able to link the cross-sample modal with other modals. All other doublet finding methods operate within a single modal. Scrublet and DoubletFinder cannot identify same-cell-type multiplets. Demuxlet cannot identify same-donor multiplets. MULTI-seq and DemuxEM cannot identify same-sample multiplets. GMM-Demux, on the other hand, bridges cross-sample multiplet identification with other modals. For example, in our manuscript, we showcased that GMM-Demux is able to identify multi-sample multiplets (cross-sample modal) in the same-cell-type clusters (cross-cell-type modal) and same-sample multiplets in multi-cell-type clusters (with the assistance of high-fidelity manual clustering in the surface marker space). Although GMM-Demux is unable to identify multi-cell-type clusters on its own, it eclipses traditional single-modal multiplet identification methods since it is able to operate beyond just the single realm of cross-sample validation.*

*This does not mean that DoubletFinder and scrublet are not important and GMM-Demux can replace them. Quite to the contrary, they are orthogonal to each other. For example, suppose that DoubletFinder generated a suite of clustering outputs (right now its clustering information is not exposed) while sweeping through a range of parameters. GMM-Demux can then be used to check the output of each set of parameters to assess whether the phony-type GEM clusters engulf too many singlets and is over-inclusive. In this manner, GMM-Demux can help DoubletFinder select the best set of parameters that maximizes phony-type multiplet detection.*

*Because GMM-Demux can operate across modals (with external help), GMM-Demux can potentially be applied to a wider range of experimental settings than DoubletFinder and scrublet. As admitted in the DoubletFinder manuscript, “DoubletFinder requires putative doublets to cluster separately from singlets in PC space, we did not expect DoubletFinder to robustly detect homotypic doublets”. For instance, DoubletFinder and scrublet have poor performance in our T-Memory dataset. This is because this dataset contains purified T-Memory cells from 5 samples. Each sample contains T-memory cells influenced by a sample-specific stimulus (external stimuli do not necessarily guarantee that there will be RNA-level differences between samples). Since there is only a single cell type, there are only homotypic multiplets in this dataset. DoubetFinder or scrublet do not perform well under this setting. While this also negates the pure-type verification function of GMM-Demux, GMM-Demux can still detect multi-sample multiplets through sample-barcoding. GMM-Demux can work in junction with other multiplet detection methods in other modals as well (with the aid of modal specific clustering algorithms), as long as the dataset contains sample-barcoding information. From this perspective, GMM-Demux has a wider applicability, although at the expense of requiring the assistance of external, modal-specific clustering algorithms.*

*To address the reviewer’s concern, we have increased the emphasis that GMM-Demux requires external, modal-specific assistance to fully realize its cell type authentication potential in the Discussion section (please refer to the response to the general comments). We also added further clarifications in the Related Works section:*

C5. As a Methods paper, the authors are limited to 4-6 main text figures. While it is naturally up to the authors how to adhere to this limitation, I suggest the following figure progression (moving other figures to SI, particularly figures referenced only in the Methods section):

I.	Figure 1: Introduction of SSM/MSM/singlet and phony-type/pure-type GEM nomenclature. Can either merge Fig. 1 with Fig. 2 or just move Fig. 1 to SI.

.	Figure 2: PBMC-1 classification results. Keep existing Fig. 3.

II.	Figure 3: Classification method performance comparison. Merge Fig. 6, Fig. 7, and Fig. S5 to make points about classification accuracy, run time, and classification result stability. Should move data simulation embeddings (including all of the simulations, not just the 4-sample simulation) to SI.

III.	Figure 4: SSM estimation verification. Keep existing Fig. 8, and perhaps incorporate Fig. S15.

IV.	Figure 5: Benchmarking phony-type GEM classifications. Visualize low-input scRNA-seq phony-type GEM verification experiment (which was a very smart experiment, by the way), and comparisons of GMM-Demux MSM and phony-type GEM classifications with DoubletFinder/Scrublet predictions.

*Response to C5: We thank the reviewer for helping us sort figures by importance. We have merged Figure 6 and 7 as suggested by the reviewer. We kept the remaining figures unchanged as permitted by the editor.*

C6.	While the comparisons between GMM-Demux and Seurat are well-executed, the authors should de-emphasize this part of their manuscript in favor of the comparisons to MULTI-seq and demuxEM. The poor performance of the Seurat classifier has already been described in numerous publications – hence the development of Solo, demuxEM, and the MULTI-seq classification pipelines. It is fine to make these points yet again in SI, but the key comparisons to focus on are between GMM-Demux and demuxEM/MULTI-seq.

*Response to C6: We thank the reviewer for this suggestion. We appreciate the reviewer for thinking on our behalf. However, given the wide popularity of Seurat and the fact that it is still one of the most dominant analytical tools used by the community including our local collaborators despite its poor performance as the reviewer pointed out, we believe it is best to keep the comparison to Seurat in the main text. We are happy to move it to the Supplementary Materials upon request by the editor.*

C7.	I agree with Reviewer #2 that the manuscript, even in its now revised form, would benefit from significant shortening. For example, using the main text to explain that “each dot in the tSNE plot represents a GEM,” that, “the color of the GEM specifies the class of the GEM,” that, ”GEMs with similar HTO UMI count profiles are more likely to be drawn close to each other,” and finally that, “GEMs with similar HTO UMI count profiles are likely to be grouped together but there is no guarantee” is excessive — especially considering the likely readership for this manuscript. This point also holds true for the subsequent paragraph where the authors note that “GEMs with higher HTO UMI counts of the sample have darker colors,” as well as numerous other points throughout the manuscript. While I appreciate the author’s thoroughness, the deluge of details detracts from the main conclusions of the work, and can be much more simply and effectively explained in the figure legends.

*Response to C7: We appreciate the above suggestions and have removed some of the excessive details throughout the manuscript as suggested. Due to the interdisciplinary nature of our work and the audience, we believe that some details are worth keeping as we think some details are not universally known by the community, such as the fact that “tSNE, as a heuristic, does not guarantee that high-dimensional proximity is always faithfully preserved after the transformation” (we have encountered suspicions about the heuristic nature of tSNE transformation numerous times from some of our wet lab colleagues during the development of GMM-Demux). We believe this could avoid some potential questions from the readers while parsing through the results. We are happy to further reduce the amount of technical details upon request by the editor.*

Minor Comments

MC1.	Pg. 6, Line 39: “Among all three classifiers, GMM-Demux and MULTI-seq adhere to the HTO heat maps the most.” This is a vague statement, either remove or clarify (perhaps with summary statistics).

*Response to MC1: We admit that it is difficult to statistically quantify the adherence to the heatmap. We have revised this sentence upon the reviewer’s request to avoid confusion.*

MC2.	Pg. 4, Line 55: Cells were cultured at 1 x 106 cells/mL, not 1 x 10-6 cells/mL.

*Response to MC2: Thanks for noting this. We have fixed the typo.*

MC3.	“Online Cell Hashing Experiment Planner” should be renamed “Online Sample Multiplexing Experiment Planner” to encompass other sample multiplexing methods beyond cell hashing.

*Response to MC3: This is a great idea. We have adopted the new name of our experiment planner. Many thanks to the reviewer.*

MC4.	The authors may consider using more descriptive Results sub-section headers which convey the main conclusion(s) of the described analyses. For example, instead of “Joint analysis with surface marker data,” the authors could title the sub-section, “GMM-Demux classifications distinguish phony- and pure-type GEM clusters.”

*Response to MC4: This is a very good suggestion. We have updated the subsection titles accordingly.*

MC5.	The authors wrote that, “we refine the selection of both CD16+ GEM groups by clipping out GEMs that disperse into the CD16- GEM cluster.” What do they mean by “clipping out”? The authors should use unsupervised clustering on the scRNA-seq data to perform this “refinement.”

*Response to MC5: We manually removed the droplets that diffused into the CD16- clusters. While we agree that unsupervised clustering in general removes human bias, for the purpose of purifying cell selection, we think it is not required and is a rather personal preference. As we purify cell selection, we are guided by both transcriptomic (with tSNE transformation) and surface marker information. Hence, we purified droplet selections based on domain knowledge and not through a random process (this is not much different than manual gating in the surface marker space). We have revised the statement to the following to emphasize human supervision.*

MC6.	Very generally, this manuscript would benefit greatly from having the tightened and typos fixed.

*Response to MC6: We appreciate the criticism. We have further polished the manuscript and have had it proof-read by a few colleagues.*

*Response to MC7: The purpose of the newly added section S12 is to address the first comment from reviewer #2 in the previous review. In the previous review, reviewer #2 asked: “Does the contamination of phony GEMs have any grave downstream effects? It is not clear why I need to care about them. I expect that emphasizing this point would increase utility of the tool”. We argue this question can also be applied to scrublet and DoubletFinder and we think that the best way to address this comment is to simply show the outcome of a downstream analysis without removing phony-type GEMs. Hence section S12.*

*As reviewer #1 pointed out in this comment, not removing phony-type GEMs would lead to high-MSM-concentration GEM clusters (as opposed to having the clustering algorithm simply distribute phony-type GEMs into pure-type GEM clusters) and we showed that it could lead to decreased clustering fidelity in the RNA space. We hope that this establishes the necessity to consider the phony-type GEMs and remov them preemptively (page 4 line 23: “After removing all the phony-type GEM clusters, GMM-Demux further reduces the multiplet rate to 3.29%”).*

MC8.	Pg. 22, Line 35: “The heuristic classifier currently is the only analytical tool for processing sample barcoding data.” This is untrue, and is an especially confusing statement since the authors state that “there are three analytical methods for processing sample barcoding” in the following sentence. Please remove.

*Response to MC8: Thanks for noting the inconsistency. We apologize for failing to notice this statement in our previous revision. We have subsequently removed this sentence.*

MC9.	Color legend is missing from Fig. S4, Fig. 4.

*Response to MC9: Legends in Figure S4 are removed intentionally as they are not informative. For the PBMC-2 dataset, there are over 80 classes (from 10 HTO samples). Even in the PBMC-1 dataset, there are 16 classes. Keeping labels for every class is not very practical nor very informative for this plot set. Instead, in the caption, we stated that “each sample and each multi-sample combination is given a unique color”. The goal of this plot set is to illustrate that GMM-Demux can assign individual classifications to multi-sample combinations and we think that this figure achieves this goal.*

MC10.	Pg. 3, Line 17: “Existence of such PBMC types defies conventional wisdom, where CD3+ CD19+ cells and CD4+ CD8+ T cells are believed to be extremely rare.” Since CD4+ CD8+ T-cells are known to exist, the authors should consider removing the term “defies conventional wisdom.” Perhaps switch to, “Existence of such PBMC types at the observed frequencies is unlikely, as CD3+ CD19+ cells and CD4+ CD8+ T cells are believed to be extremely rare.”

*Response to MC10: This is great suggestion. We have updated the sentence as suggested.*

MC11.	Pg. 3, Line 44: “Most importantly, they cannot determine whether an alleged novel-cell-type-defining GEM cluster consists of mainly pure-type GEMs. Hence they cannot use MSM classification results to verify the legitimacy of putative novel cell types in scRNA-seq.” This statement is a bit misleading, as the existing sample classification algorithms do not aim to use the classification results to test for cluster legitimacy. This is like saying that unsupervised clustering does not identify differentially-expressed genes between clusters – clustering is a necessary first-step before DGE analysis, but the main goal is not DGE analysis.

*Response to MC11: What we are trying to convey in the above statement, is that beyond sample demultiplexing and MSM identification, GMM-Demux packs a droplet formation model which can further utilize the MSM information to authenticate the existence of putative cell types. MULTI-seq, Seurat (its MSM classifier module) and demuxEM are soley MSM classifiers but GMM-Demux is more than just an MSM classifier.*

*With that being said, the concern raised by the reviewer is legitimate and we acknowledge it. It is not our intention to associate inability to perform cell type authentication with MSM classification method design. To address the reviewer’s concern, we have revised the above statement as follows:*

Reviewer #2:

Summary: GMM-Demux is a computational pipeline which serves three purposes which the authors expand upon in the revised manuscript.

I commend the authors for their efforts to address my concerns.

C1, however, remains partially unsolved. As the authors point out, phony GEMs rarely form isolated clusters. The question remains how large of an impact these phony-type GEMs have on the inferred expression profiles of given clusters. The authors should compare expression profiles derived from clusters before/after removing phony-type GEMs. Does the inclusion of phony-type GEMs lead to ‘wrong’ marker gene lists? How is differential expression analysis impacted?

*Response to Reviewer #2: We greatly thank the reviewer for the commendation of our previous manuscript revision, and we are very grateful to the comments from the reviewer in the previous review. We believe that they have drastically increased the quality of our work.*

*Since the correspondence between the RNA clustering result and surface-marker-facilitated cell type annotation result is too low in the phony-type-GEM inclusive dataset (~50% AMI score), we test the impact of phony-type GEMs on differential expression (DE) analysis under a simplified setting, using only the T cells (including both the CD4+ and the CD8+ T cells). We constructed three DE analysis configurations: DE analysis of surface-marker annotated ground truth. DE analysis of purified pure-type GEMs and DE analysis of the all-inclusive T cell GEMs. The UMAP plots of the pure-type-only and the all-inclusive settings are provided below:*

*The DE analysis output between the surface-marker-annotated CD4+ and the CD8+ GEM groups is provided below. We ranked DE genes by significance and showed the top 15-ranking feature genes in each GEM group. We use this DE analysis output as the ground truth.*

*We then performed unsupervised clustering using scanpy, and then performed DE analysis again between the two clusters. The clustering results of the pure-type-only and the all-inclusive GEM populations are shown below:*

*We then performed DE analyses between the clusters in both figures. The DE analysis output of the pure-type-only GEM population is provided below:*

*Similarly, the DE analysis output of the all-inclusive GEM population is shown below*

*Between the two DE analysis outputs, the DE analysis output of the pure-type-only GEM population is more in concordance with the ground truth. For instance, the CD8B gene (highlighted in red boxes) was the No.1-ranking significant DE gene of the CD8+ cell type in ground truth but was completely excluded from the top 10 DE gene list of the CD8+ cluster in the all-inclusive DE analysis output. In contrast, the DE analysis of the pure-type-only population kept the CD8B gene in the top 10 DE gene list of the CD8+ GEM population, albeit down-ranking it to No.5. We also observe a similar trend for the LTB gene (highlighted in blue boxes) in the CD4+ GEM population.*

*We hope the above analysis sufficiently addresses Reviewer 2’s concern and further supports the removal of phony-type GEMs.*

*Additional comments*

*GMM-Demux takes post-filtering, non-empty droplets as inputs. For completeness, we have made the following updates in the main text:*

*The additional colonscopic biopsy cell hashing dataset is excluded from the main results due to privacy constraints. We have added the following to the Discussion section and the data availability section.*

*References*

*[1]	“Standardizing immunophenotyping for the Human Immunology Project | Nature Reviews Immunology.” https://www.nature.com/articles/nri3158 (accessed May 26, 2020).*

*[2]	C. S. McGinnis, L. M. Murrow, and Z. J. Gartner, “DoubletFinder: Doublet Detection in Single-Cell RNA Sequencing Data Using Artificial Nearest Neighbors,” Cell Syst., vol. 8, no. 4, pp. 329-337.e4, Apr. 2019, doi: 10.1016/j.cels.2019.03.003.*

*[3]	S. L. Wolock, R. Lopez, and A. M. Klein, “Scrublet: Computational Identification of Cell Doublets in Single-Cell Transcriptomic Data,” Cell Syst., vol. 8, no. 4, pp. 281-291.e9, Apr. 2019, doi: 10.1016/j.cels.2018.11.005.*

*[4]	J. Alquicira-Hernandez, A. Sathe, H. P. Ji, Q. Nguyen, and J. E. Powell, “scPred: accurate supervised method for cell-type classification from single-cell RNA-seq data,” Genome Biol., vol. 20, no. 1, p. 264, Dec. 2019, doi: 10.1186/s13059-019-1862-5.*

*[5]	F. A. Wolf, P. Angerer, and F. J. Theis, “SCANPY: large-scale single-cell gene expression data analysis,” Genome Biol., vol. 19, no. 1, p. 15, Feb. 2018, doi: 10.1186/s13059-017-1382-0.*

*[6]	S. E. Finder, E. Treister, and O. Freifeld, “Effective Learning of a GMRF Mixture Model,” ArXiv200509030 Cs Stat, May 2020, Accessed: May 26, 2020. [Online]. Available: http://arxiv.org/abs/2005.09030.*

*[7]	B. Krawczyk, “Learning from imbalanced data: open challenges and future directions,” Prog. Artif. Intell., vol. 5, no. 4, pp. 221–232, Nov. 2016, doi: 10.1007/s13748-016-0094-0.*

*[8]	“MULTI-seq: sample multiplexing for single-cell RNA sequencing using lipid-tagged indices | Nature Methods.” https://www.nature.com/articles/s41592-019-0433-8 (accessed May 26, 2020).*

*[9]	Y. J. Wang et al., “Comparative analysis of commercially available single-cell RNA sequencing platforms for their performance in complex human tissues,” Genomics, preprint, Feb. 2019. doi: 10.1101/541433.*

**Third round of review**

**Reviewer 1**

The authors have done a fantastic job with the revised manuscript. I especially appreciate how receptive the authors were to my comments, which I admit were fairly excessive. I also appreciate how the authors revised their manuscript to make the distinction between cell-type authentication and doublet detection more clear. As the author of DoubletFinder and MULTI-seq, I believe my initial impressions of GMM-Demux were pigeon-holed, and now am convinced that the manner in which GMM-Demux leverages multiple data modalities (e.g., -omic and sample barcoding counts) to contextualize scRNAseq data is creative, well-executed, and has significant interest and utility to the single-cell genomics community.

Chris McGinnis
